# Keeping Pace with Wearables: A Living Umbrella Review of Systematic Reviews Evaluating the Accuracy of Consumer Wearable Technologies in Health Measurement

**DOI:** 10.1007/s40279-024-02077-2

**Published:** 2024-07-30

**Authors:** Cailbhe Doherty, Maximus Baldwin, Alison Keogh, Brian Caulfield, Rob Argent

**Affiliations:** 1 School of Public Health, Physiotherapy and Sports Science, University College Dublin, Dublin, Ireland; 2 Insight SFI Research Centre for Data Analytics, University College Dublin, Dublin, Ireland; 3 Institute for Sport and Health, University College Dublin, Dublin, Ireland; 4 School of Medicine, Trinity College Dublin, Dublin, Ireland; 5 School of Pharmacy and Biomolecular Sciences, Royal College of Surgeons (RCSI), University of Medicine and Health Sciences, Dublin, Ireland

## Abstract

**Background:**

Consumer wearable technologies have become ubiquitous, with clinical and non-clinical populations leveraging a variety of devices to quantify various aspects of health and wellness. However, the accuracy with which these devices measure biometric outcomes such as heart rate, sleep and physical activity remains unclear.

**Objective:**

To conduct a ‘living’ (i.e. ongoing) evaluation of the accuracy of consumer wearable technologies in measuring various physiological outcomes.

**Methods:**

A systematic search of the literature was conducted in the following scientific databases: MEDLINE via PubMed, Embase, Cinahl and SPORTDiscus via EBSCO. The inclusion criteria required systematic reviews or meta-analyses that evaluated the validation of consumer wearable devices against accepted reference standards. In addition to publication details, review protocol, device specifics and a summary of the authors’ results, we extracted data on mean absolute percentage error (MAPE), pooled absolute bias, intraclass correlation coefficients (ICCs) and mean absolute differences.

**Results:**

Of 904 identified studies through the initial search, 24 systematic reviews met our inclusion criteria; these systematic reviews included 249 non-duplicate validation studies of consumer wearable devices involving 430,465 participants (43% female). Of the commercially available wearable devices released to date, approximately 11% have been validated for at least one biometric outcome. However, because a typical device can measure a multitude of biometric outcomes, the number of validation studies conducted represents just 3.5% of the total needed for a comprehensive evaluation of these devices. For heart rate, wearables showed a mean bias of ± 3%. In arrhythmia detection, wearables exhibited a pooled sensitivity and specificity of 100% and 95%, respectively. For aerobic capacity, wearables significantly overestimated VO_2max_ by ± 15.24% during resting tests and ± 9.83% during exercise tests. Physical activity intensity measurements had a mean absolute error ranging from 29 to 80%, depending on the intensity of the activity being undertaken. Wearables mostly underestimated step counts (mean absolute percentage errors ranging from − 9 to  12%) and energy expenditure (mean bias =  − 3 kcal per minute, or − 3%, with error ranging from − 21.27 to 14.76%). For blood oxygen saturation, wearables showed a mean absolute difference of up to 2.0%. Sleep measurement showed a tendency to overestimate total sleep time (mean absolute percentage error typically > 10%).

**Conclusions:**

While consumer wearables show promise in health monitoring, a conclusive assessment of their accuracy is impeded by pervasive heterogeneity in research outcomes and methodologies. There is a need for standardised validation protocols and collaborative industry partnerships to enhance the reliability and practical applicability of wearable technology assessments.

**Prospero ID:**

CRD42023402703.

**Supplementary Information:**

The online version contains supplementary material available at 10.1007/s40279-024-02077-2.

## Key Points

**Table:** 

Wearable technologies are widely used in healthcare and wellness settings, offering continuous monitoring of various biometric parameters such as heart rate, physical activity, sleep patterns and more.
Many studies have investigated the accuracy of consumer wearables against established criterion standards. However, the results are varied due to differences in methodologies, devices, and targeted biometric outcomes, complicating the effort to develop a unified understanding of wearable accuracy.
We estimate that approximately 11% of the 310 consumer wearables that have been released to date have been validated for at least one biometric outcome; approximately 3.5% of biometric outcomes have been validated for these devices.
This research uncovers significant variability in device accuracy and measured outcomes, underscoring inconsistencies. It emphasizes the need for a standardized, rigorous, and adaptable validation protocol in the rapidly evolving field of wearable technologies.
By offering a ‘living’ review, this study aims to maintain an up-to-date representation of the wearable technology validation landscape, facilitating informed decision-making for researchers, clinicians and consumers navigating this domain.

## Introduction

Consumer wearable devices—such as watches, wristbands, pendants, glasses, armbands and other accessories—are rapidly permeating various aspects of daily life, becoming ubiquitous tools for monitoring, assessing and enhancing human behaviour and health [1]. These devices encapsulate a wide array of sensors and software, facilitating the continuous collection of individualised data including physical activity, heart rate, sleep patterns and even mood states [2]. The increasing pervasiveness of wearable technology is demonstrated by its global market size, which is expected to reach US$186.14 billion by 2030, expanding at a compound annual growth rate of 14.6% from 2023 to 2030 [3]. The American College of Sports Medicine (ACSM) also recently identified wearable technologies as the ‘#1 fitness trend’ for 2024 on the basis of a survey of > 4500 health and fitness professionals [4].

Wearables are now heralding a new epoch in several fields of research too; they are generally unobtrusive, cost-effective and comfortable to wear and yield a high level of acceptability among users [5–7]. This is reflected in the proliferation of research incorporating wearable technologies for remote data capture. For instance, the Datenspende study by the Robert Koch Institute deployed wearables to tackle the coronavirus disease 2019 (COVID-19) pandemic through anonymous data donations [8], while Perez et al. 2019 exhibited the capacity of the Apple Watch to detect atrial fibrillation [9], sparking discussions on the potential and limitations of these devices among healthcare providers, researchers and media. Kimura et al. investigated associations between steps and sleep patterns, collected with a wristband, and markers of Alzheimer’s disease in older adults [10], while Shilaih et al. utilised wristbands to correlate heart rate and wrist temperature with menstrual cycle phases [11]. Wearables have also started to be incorporated in clinical trials: at the time of writing, there are currently 58 active trials on clinicaltrials.gov using an Apple Watch, 323 using a Fitbit and 71 using a Garmin device [12].

This research demonstrates how, by allowing for long-term data collection in naturalistic environments, wearables facilitate ecological momentary assessments, and can generate insights into individual health patterns [13, 14]. But despite their potential, the relentless pace of technological advancements and the multifaceted nature of these devices raise pertinent questions about their validity and reliability [15, 16]. For researchers, accurate data are crucial for robust study methodologies, especially when these devices are used to infer health status or predict disease outcomes [16, 17]. For healthcare providers, the validity of data can directly affect clinical decisions, patient care and monitoring, and could ultimately impact health outcomes [18]. For individuals, accurate self-monitoring could potentially help shape health-related behaviours and lifestyle modifications, fostering a sense of empowerment and facilitating personal health management [19].

Numerous factors can impact the validity of data collected by wearable devices. These include variability in the algorithms used by different devices to estimate metrics such as heart rate, sleep or physical activity [20]; user-specific factors such as age, body size or skin tone [21]; and differences in device placement and wear time [22]. Environmental conditions, such as temperature and humidity, can also affect sensor performance [23]. Perhaps most importantly, the dynamic nature of the wearable technology industry—with new devices, software updates and algorithms being continually introduced—necessitates ongoing validation studies [24–27]. As such, ensuring the accuracy and validity of wearable devices is an ongoing endeavour, one that has been taken on in a number of research initiatives. For instance, the International Federation of Sports Medicine (FIMS) has advocated for a global standard for sport and fitness wearables amidst the rising concerns for quality assurance related to the products [28], while the INTERLIVE network is a joint European initiative focused on developing best-practice recommendations for evaluating the validity of consumer wearables to measure direct and derived metrics [25–27].

However, the pace of academic research struggles to keep up with the more agile commercial ecosystem: primary research studies are slowed by the need to secure funding, develop validation protocols, recruit and test participants and navigate the peer review process [16], while systematic reviews and meta-analyses are often out of date by the time they are published [28]. Commercial entities typically release new hardware annually and push software updates multiple times a year, vastly outpacing the academic validation and synthesis cycle [16]. This disparity underlines the need to make the research in this field ‘living’ and continually updated, leveraging methodologies such as living systematic reviews to maintain pace with the rapidly evolving technological landscape [29]. The vision of a ‘living’ body of research in the wearable technology field champions the principles of real-time evidence synthesis, capable of dynamically incorporating new findings as they emerge [30].

The aim of this study is to conduct a systematic review of systematic reviews evaluating the accuracy (i.e. validity and/or reliability) of consumer wearable technologies. This review focuses on consumer wearables rather than research wearables, as these devices have the largest user base and undergo regular updates in both software and hardware. The review is not constrained by setting, reflecting the widespread use of wearables among the general population, athletes and clinical populations. This review will be ‘living’, meaning it will be updated as new systematic reviews are published. Our objectives are as follows: (1) to evaluate what biometric outcomes consumer wearable technologies can measure, including metrics such as heart rate, physical activity, sleep and stress; (2) to synthesise the accuracy (including validity and reliability) of consumer wearable technologies for the outcomes in (1); and (3) to evaluate the methodological quality of the existing systematic reviews on this topic. This approach will allow us to identify potential gaps in the literature and provide recommendations for future research. By doing so, this review will bridge the gap between the rapid pace of wearable technology development and the slower tempo of academic research, ensuring that decision-making in this field is informed by the best available evidence.

## Methods

### Design

This umbrella review has been written according to the Preferred Reporting Items for Systematic Reviews and Meta-analyses (PRISMA) guidelines [31]. The protocol was registered on PROSPERO (CRD42023402703) on 18 March 2023.

### Search Strategy

A literature search was conducted on 5 June 2024 across the following databases: MEDLINE via PubMed, Embase, Cinahl and SPORTDiscus via EBSCO. We sought to identify systematic reviews evaluating the accuracy of consumer wearable devices compared with criterion measures. No filters were applied during the search. Authors C.D. and M.B., with the support of our institution’s librarian, devised and tailored the search strategies for each database. The strategy for MEDLINE was developed initially and later adapted for other databases using database-specific terminologies. The full search strategies are detailed in Supplemental file 1.

### Study Selection Strategy

After the literature search, all identified studies were imported into Endnote 21 and subsequently exported to the Covidence systematic review software. Two authors (CD and MB) independently screened all articles through title/abstract screening, full-text screening and data extraction phases. Disagreements between CD and MB were resolved through discussion, and if consensus could not be reached, the supervising author (RA) provided the decisive opinion.

### Eligibility Criteria

Included studies had to satisfy the following criteria: (1) they must be a systematic review and/or meta-analysis, (2) they must include studies that evaluated the validation of consumer wearable devices, (3) the research synthesis in the review must focus on validity or accuracy of consumer grade wearables specifically and (4) devices’ validation must be against an accepted reference standard. Consumer wearables were defined as any body-worn device available for public purchase, including those previously available but now discontinued. The primary exclusion criteria were: (1) validation of research-grade or non-consumer wearable devices and (2) validation of wearables against other wearables (i.e. convergent validity only). No restrictions were imposed on study populations, device wearing locations or biometric types, as long as the aforementioned criteria were met.

### Risk of Bias

The methodological quality of each included systematic review was assessed by two independent investigators using the Risk of Bias Assessment Tool by Drucker et al. [32]. This tool identifies six bias domains: protocol pre-registration, evidence selection, bias assessment in the studied reviews, competing interests, ‘spin’ (i.e. misleading reporting, interpretation or extrapolation of results) and interpretation of findings. Reviews scoring 6/6 were deemed to be of high quality, providing an accurate and comprehensive summary of the results of the available studies that address the question of interest. Reviews scoring 4–5/6 were considered moderate quality, indicating more than one weakness but no critical flaws. Reviews scoring 2–3/6 were classified as low quality, indicating the presence of one critical flaw with or without non-critical weaknesses. Finally, reviews scoring less than 2/6 were deemed to be of critically low quality, indicating more than one critical flaw with or without non-critical weaknesses.

### Data Extraction

Data extraction from eligible studies was undertaken by authors CD and MB. Extracted data comprised: (1) publication/author details [digital object identifier (DOI), title, authors, corresponding author’s contact, publication year, country of the first author and funding sources]; (2) review protocol [review criteria, target population, targeted outcome, research setting (free-living or controlled), sample size, sex distribution, age and health status]; (3) device-specific information (criterion measure, device brand and data acquisition protocol); and (4) results (summary of validity and authors’ conclusions). Where additional details were required, corresponding authors were approached via email.

### Data Synthesis

To synthesise the results, we extracted and prioritised specific statistics from the included systematic reviews, focusing on metrics that allowed for meaningful comparison across studies. The primary statistics extracted included mean absolute percentage error (MAPE), pooled absolute bias, intraclass correlation coefficients (ICCs) and mean absolute differences. Where available, confidence intervals (CI) were also extracted to provide context to the reported metrics. In cases where multiple reviews covered the same biometric outcome, we prioritised data from reviews on the basis of their methodological quality, as assessed by the Risk of Bias Assessment Tool outlined above. Reviews deemed to be of high quality were given precedence in the synthesis, followed by those of moderate and then low quality. This hierarchical approach ensured that the most reliable and robust data informed our conclusions.

For biometric outcomes with varied reporting metrics, we standardised the data presentation to enhance clarity and comparability. For instance, where multiple forms of error metrics were reported (e.g. bias and ICC), we opted for the statistic most frequently used across reviews to maintain consistency. We cross-checked the primary research studies included in each systematic review to identify and remove duplicates, ensuring that each original study was only counted once in our synthesis. In instances where numerical data were not provided (e.g. descriptive summaries of sleep metrics), we included these qualitative assessments but noted the absence of specific statistical measures.

### Living Review Implementation

Given the dynamism in the wearable technology sector and the associated research [16], we plan to continually update this synthesis. This will be achieved through a regular search update, whereby we will perform systematic searches every 6 months across all predetermined databases. Any new systematic reviews identified will be screened for eligibility and, if suitable, will be incorporated into the living review. Upon identification of new eligible systematic reviews, the same extraction method described above will be employed. Data from new reviews will be synthesised with the existing evidence. We will upload review updates to OSF.io (https://osf.io/fqvms/?view_only=e49e7c42dfd3475db5cf2da3b15e4b3f). Every update will result in a new version of the living review. Each version will have a unique identifier and a changelog to outline the differences from the previous version. All versions of the review will be made accessible to readers, allowing them to trace the evolution of evidence over time. The need for the review to remain ‘living’ will be assessed annually. Factors considered will include the pace of emerging evidence, the evolution of wearable technologies and feedback from the community. If there is a reduction in the pace of new evidence or if the topic reaches a point of saturation, the review might transition from a ‘living’ status to a traditional static review.

## Results

### Literature Search

**Fig. 1:**
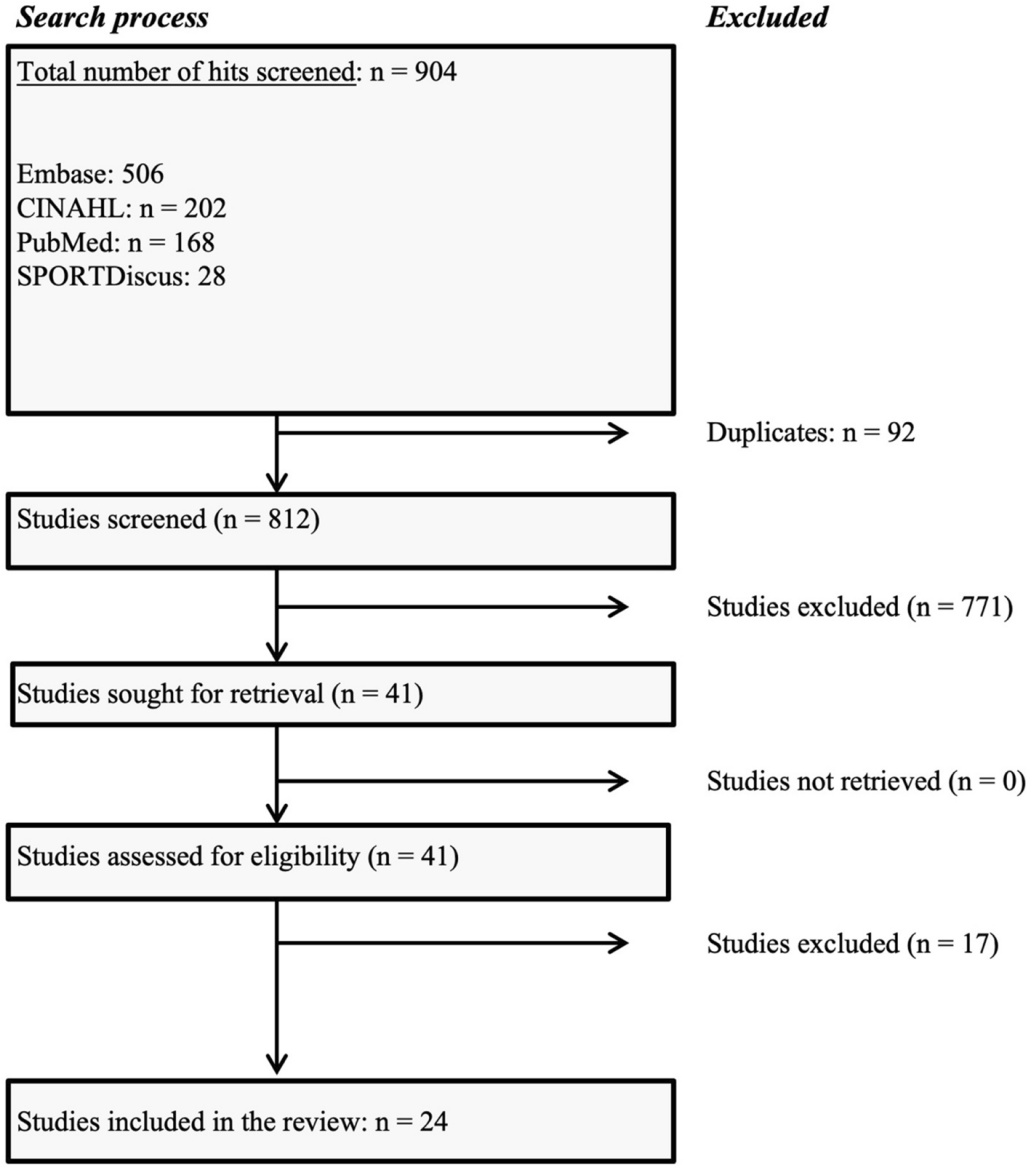
Preferred Reporting Items for Systematic reviews and Meta-Analyses (PRISMA) flow diagram

From an initial pool of 904 studies identified for the review, 92 duplicates were removed, leaving 812 studies to be screened by title and abstract. Of these, 771 did not meet the inclusion criteria and were excluded. This resulted in 41 studies that were assessed for full-text eligibility. Upon full-text assessment, 17 of these studies were further excluded: 11 did not discuss commercially available wearables as distinct entities, 4 had no available full text (for instance, conference submissions), and 2 utilised a dataset duplicated in another review. Consequently, a total of 24 systematic reviews (8 of which performed some kind of meta-analysis) were included in this umbrella review [15, 33–55]. The process of study selection is depicted in the PRISMA flow diagram (Fig. 1).

### Characteristics of the Systematic Reviews Included in the Umbrella Review

The 24 reviews collectively included 653 studies. The eligible systematic reviews (16 out of 24) and meta-analyses (8 out of 24) were published between 2013 and 2024. Due to the overlap of studies included in different reviews, we extracted the following information from the primary research: (1) publication/author details (DOI, title, authors, publication year), (2) population group (target population, age range, number of males/females), (3) biometric outcome studied (e.g. heart rate), (4) wearable device used (including device manufacturer and name), and (5) criterion measure used for comparison and (6) statistical analysis.

After removing duplicated studies across the reviews, 391 primary research studies of 888,033 participants were identified. However, only 249 of these were validation studies of consumer-grade wearable devices, collectively involving 430,465 participants (243,068 males and 181,064 females; 80 studies did not provide sufficient detail to determine the sex distribution of the participants).

The characteristics of the 24 reviews, including the population groups, number of participants, biometric outcomes of interest and criterion measures used, are presented in Table 1. The full characteristics of the dataset, including the narrative synthesis of the results and authors’ conclusions, are available in Supplemental File 2. Article metadata for the individual studies included in the 24 reviews is also available in Supplemental File 2.

**Table 1:** Characteristics of included systematic reviews

Study	Number of studies	Population	Number of participants	Biometric(s)	Criterion measure(s)
Belani et al. (2021) [33]	9	Patients with atrial fibrillation	1629	Atrial fibrillation	12-lead ECG, 7-lead Holter monitor, ICM recording and telemetry
Board et al. (2016) [34]	12	Healthy adults	595	(1) Detecting inter-beat intervals and error associated, and (2) heart rate variability readings	ECG (2-, 3-, 5- and 12-lead)
Byrne et al. (2023) [35]	7	Wheelchair users (able-bodied included)	126	Wheelchair push counts	Direct observation (video recorded or not)
Chevance et al. (2022) [36]	52	Healthy adults; some patients populations (cardiac and/or respiratory conditions, chronic pain and Parkinson disease)	1628	(1) Energy expenditure, (2) heart rate and (3) step count	*Energy expenditure*: Doubly labelled water, indirect and direct calorimetry. *Heart rate*: ECG, pulse oximetry and specific chest worn straps (Polar). *Steps*: direct observation (video recorded or not)
Evenson et al. (2015) [37]	22	Healthy adults	593^*^	(1) Energy expenditure, (2) step count, (3) Distance, (4) sleep, (5) physical activity	*Steps*: Manual counting (controlled settings), accelerometers and pedometers (free-living settings). *Energy expenditure*: indirect and direct calorimetry (controlled), accelerometers (ActiGraph and BodyMedia SenseWear; free-living settings). *Sleep*: PSG. *Distance*: Treadmill distance
Feehan et al. (2018) [38]	67	Healthy adults; some participants with mobility limitations and/or chronic diseases	2441	(1) Step count, (2) energy expenditure, (3) sleep and (4) time in activity	*Step count (controlled setting)*: Direct observations and manual counting. *Energy expenditure (controlled setting)*: Direct and indirect calorimetry (free-living settings), doubly labelled water and accelerometers. *Sleep*: PSG over 1 night in a sleep laboratory
Fuller et al. (2020) [15]	169	Healthy adults; some participants with mobility limitations and/or chronic diseases	5934	(1) Energy expenditure, (2) heart rate and (3) step count	*Step count (controlled setting)*: Manual counting and accelerometry. *Heart rate (controlled setting)*: ECG, Polar chest straps and pulse oximetry. *Energy expenditure (controlled setting)*: Direct and indirect calorimetry. *Step count (free-living settings)*: Accelerometry. *Heart rate (free-living settings)*: Polar chest strap. *Energy expenditure (free-living settings)*: Doubly labelled water (1 study) and accelerometry
Georgiou et al. (2018) [39]	18	Healthy adults	1680	Heart rate variability	ECG and Holter monitors
Germini et al. (2022) [40]	65	Healthy adults and children; patient populations	4791	(1) Step count, (2) heart rate, (3) energy expenditure and (4) time in activity	*Step count*: Manual counting either by direct observation or through video. *Heart rate*: ECG, pulse oximetry and other activity trackers (4 different devices, NR). *Energy expenditure:* Indirect calorimetry. *Time in activity*: Research grade accelerometers including but not limited to Actigraph and GENEActiv
Giebel & Gissel (2019) [41]	22	In-patients	206	Atrial fibrillation	ECG/Holter recordings
Haghayegh et al. (2019) [42]	22	Healthy adults; participants with mobility limitations and/or chronic diseases	438	Sleep	PSG
Henriksen et al. (2020) [43]	14	Healthy adults; participants with mobility limitations and/or chronic diseases	456	(1) Step count, (2) energy expenditure and (3) physical activity intensity levels	*Step count (controlled setting)*: Direct observations and manual counting. *Step count (free-living)*: Accelerometry. *Energy expenditure (controlled setting)*: Direct and indirect calorimetry. *Energy expenditure (free-living settings)*: Accelerometry. *Physical activity intensity levels (free-living conditions):* Accelerometers
Hermans et al. (2021) [44]	46	Patients with atrial fibrillation	NR	Atrial fibrillation	Not specified but included 24-h Holter monitor, 12-lead ECG or single-lead ECG
Irwin & Gary (2022) [45]	8	Healthy adults	256	Heart rate and step count	ECG and polar strap
Kenyon et al. (2013) [46]	7	Adults and children with physical disability	197	Step count	*Step count*: Manual counting either by direct observation or through video
Koerber et al. (2022) [47]	10	Healthy adults	469	Heart rate and cardiac arrhythmias	*Heart rate*: ECG and chest straps (no model provided)
Leung et al. (2021) [48]	29	Healthy adults	927	Energy expenditure	*Energy expenditure*: Indirect calorimetry
Lopez Perales et al. (2021) [49]	43	Healthy adults	10,974	Atrial fibrillation	12-lead ECG, pacemaker and implantable cardioverter-defibrillator electrograms and cardiac telemetry monitoring
Molina-Garcia et al. (2022) [50]	14	Healthy adults	403	Aerobic capacity (VO_2_ max)	Direct or indirect calorimetry (gas analysis)
Nazarian et al. (2021) [51]	18	Participants with cardiac arrhythmia	424,371	Cardiac arrhythmias	12-lead ECG in most cases, a Holter monitor, ECG patch, telemetry or internet-enabled mobile ECG
O’Driscoll et al. (2018) [52]	64	Healthy adults	1946	Energy expenditure	Doubly labelled water, indirect calorimetry systems and metabolic chambers
Schyvens et al. (2024) [53]	8	Healthy adults; participants with Huntington’s disease	141	Sleep	PSG
Windisch et al. (2023) [54]	5	Healthy adults in hypoxic conditions; adults and children with congenital heart disease and lung disease	973	Blood oxygen saturation	Pulse oximetry
Zhang et al. (2020) [55]	44	Healthy adults	1738	Heart rate	ECG/chest strap
NR = Not reported					

*ECG* electrocardiogram, *ICM* implantable cardiac monitor, *PSG* polysomnography, *NR* not reported

### Overview of Consumer Wearable Devices and Validation Status

To determine the number of consumer wearable devices that have been validated to date, we first compiled a list of devices that have been on the market since 2003 (the year the Garmin Forerunner 101 was released). This list includes 310 consumer wearable devices released by the following manufacturers: Amazfit (21), Apple (14), Coros (8), Fitbit (32), Fossil (11), Garmin (72), Google (2), Huawei (24), Jawbone (6), Mi (10), Misfit (2), Oura (3), Polar (24), Redmi (3), Samsung (24), Skagen (2), Suunto (13), TicWatch (15), Whoop (4) and Withings (20).

Of these 310 devices, 34 (11%) were validated for at least one biometric outcome in at least one of the primary research studies included in the reviews identified via our search. The most commonly validated device manufacturers were Fitbit (31 studies), Apple (12 studies) and Polar (6 studies).

However, most consumer wearables can evaluate a multitude of biometric outcomes, with some of the most common being step count, heart rate, sleep, physical activity and energy expenditure. Assuming that each wearable in our list of 310 devices can measure these five biometric outcomes, the potential number of discrete validation studies required to have a complete picture of consumer wearable validity is 1550 (i.e. 310 devices × 5 outcomes). The actual number of validity studies that have been conducted on these wearables for the specified biometric outcomes is 54, representing only 3.5% of the total number of potential validations.

The full details of the devices that have been validated linked in specific studies are available in Supplemental file 2.

### Credibility of Evidence of Measurement Using Wearable Devices

The results of the risk-of-bias (RoB) assessment are presented in Table 2. Only two reviews (8%, both of which included meta-analyses) showed a high quality of evaluation; 10 (42%) evaluations were of moderate quality, and a further 12 (50%) were deemed to be of low quality. No reviews were deemed to be of critically low quality. In the following sections, we mainly describe the results of evaluations that performed a meta-analysis (where available) of the highest available quality (i.e. sequentially, on the basis of the availability of high, moderate and finally low quality).

**Table 2:**
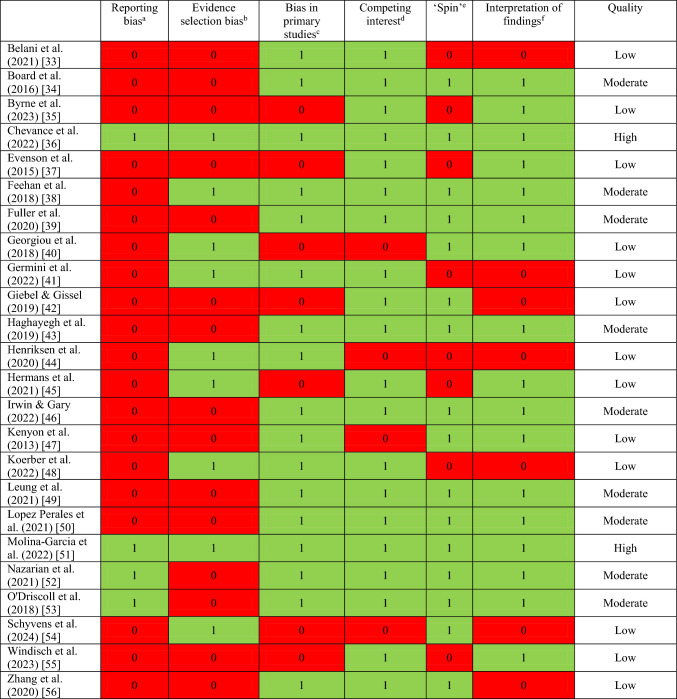
Results of the risk of bias assessment

^a^Reporting bias: Was the protocol for the review pre-registered, and if so, was it adhered to?

^b^Evidence selection bias: Did the author conduct a search of the grey literature?

^c^Bias in primary studies: Was a risk of bias assessment of the primary research studies conducted? Were any sensitivity analyses performed?

^d^Competing interest: Is there a conflict of interest statement? Were funders acknowledged?

^e^‘Spin’: Is there any misleading reporting (e.g. not fully reporting the methods used to collect data), misleading interpretation (e.g. discussing nonsignificant results as if they were significant) or inappropriate extrapolation (e.g. application of the study results to a patient population not actually studied in the systematic review)?

^f^Interpretation of findings: Do the authors discuss the precision or uncertainty of a meta-analysis in terms of the 95% confidence interval of the summary effect estimate/did they evaluate the degree to which the results of primary studies included in the systematic review are consistent with each other?

### Summary of Evaluations

**Fig. 2:**
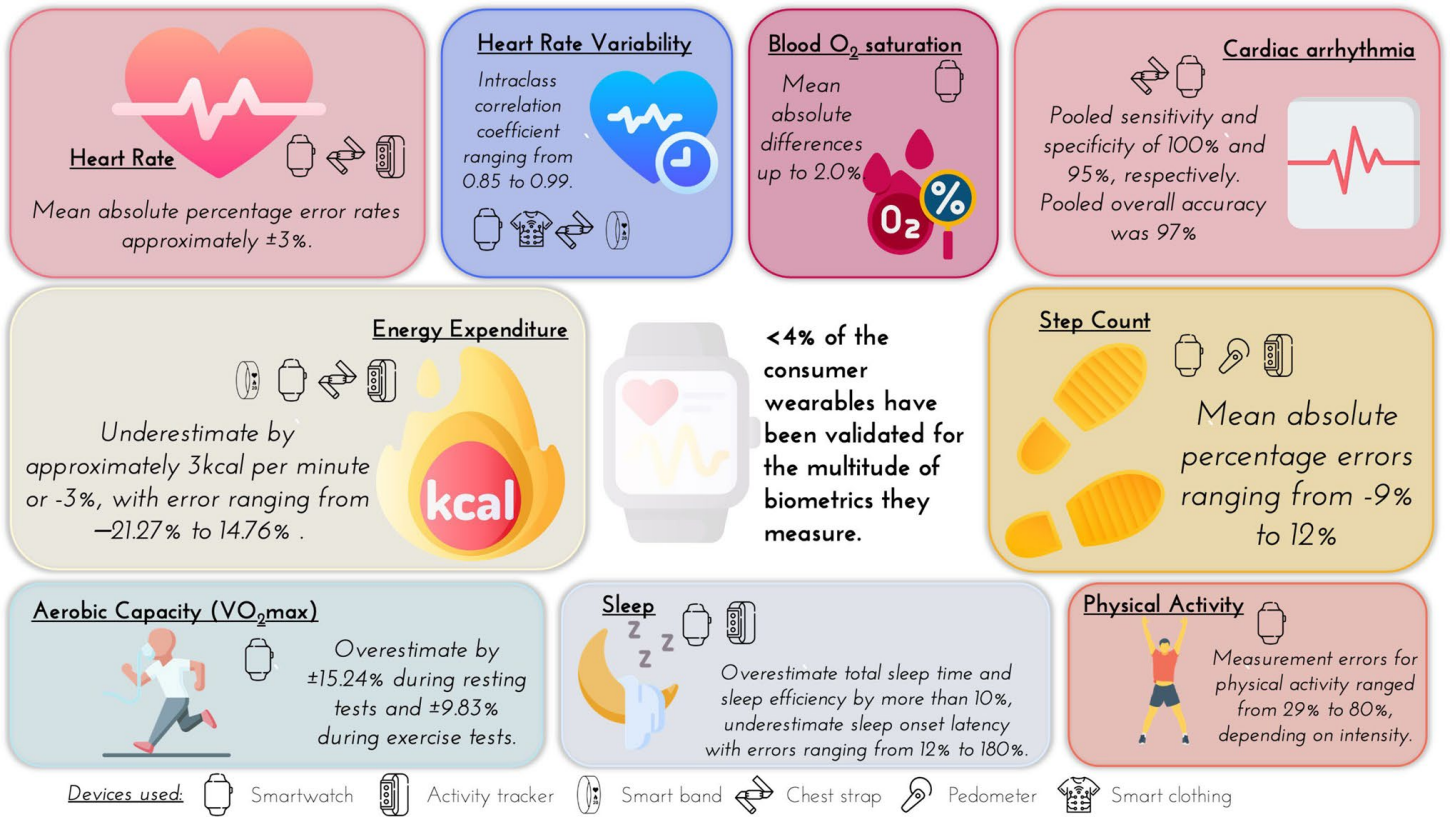
Biometric outcomes and wearable devices evaluated in each review

The 24 systematic reviews evaluated 11 biometric outcomes across three broad domains, i.e. cardiovascular, physical activity and sleep; 15 reviews evaluated a single biometric outcome, with 9 reviews evaluating more than one. The biometric outcomes evaluated included: heart rate (six reviews [15, 36, 40, 45, 47, 55]), heart rate variability (two reviews [34, 39]), cardiac arrhythmia (six reviews [33, 41, 44, 47, 49, 51]), aerobic capacity (one review [50]), blood oxygen saturation (one review [54]), step counting (eight reviews [15, 36–38, 40, 43, 45, 46]), wheelchair push counts (one review [35]), physical activity duration (four reviews [37, 38, 40, 43]), energy expenditure (eight reviews [15, 36–38, 40, 43, 48, 52]) and sleep (four reviews [37, 38, 42, 53]) The specific devices included in the studies identified by each review and the biometric outcome(s) they were validated against are presented in Table 3 and illustrated in Fig. 2.

**Table 3:** Biometric outcomes and wearable devices evaluated in each review

Study	Cardiorespiratory					Physical activity				Sleep		Wearable device(s) evaluated
	HR	HRV	AF	VO_2max_	SpO_2_	Steps/wheelchair pushes	PA	EE	Distance	Time (onset, etc.)	Stages	
Belani et al. (2021) [33]			✓									Apple Watch, Kardiaband (device NR), Samsung (device NR)
Board et al. (2016) [34]		✓										Polar (V800, RS800CX, 810) and Suunto (T6)
Byrne et al. (2023) [35]						✓						Apple Watches of different generations, Garmin VivoFit, Fitbit Flex, Fitbit Flex 2 and Jawbone UP24
Chevance et al. (2022) [36]	✓					✓		✓				Fitbit (Charge HR, Charge 2, Blaze, Surge, Versa, Charge 3 and Ionic)
Evenson et al. (2015) [37]						✓	✓	✓	✓	✓		Fitbit (Ultra, One, Zip, Flex, Force, Charge, Surge and Charge HR) and Jawbone (UP, UP24, UP MOVE, UP2, UP3 and UP4)
Feehan et al. (2018) [38]						✓	✓	✓		✓		Fitbit (Ultra, Classic, Zip, One, the Flex, Charge HR, Force and Surge)
Fuller et al. (2020) [15]	✓					✓		✓				Apple Watch (Series 1 and 2), Fitbit (Alta, Blaze, Charge, Charge 2, Charge HR, Classic, Flex 1 and 2, Force, One, Surge, Ultra and Zip) and Garmin (Fenix 3HR, Forerunner 225, Forerunner235, Forerunner 405CX, Forerunner 735XT, Forerunner 920XT, Vivoactive, Vivofit 1, 2 and 3, Vivosmart and HR and HR +), Mio (Alpha, Fuse, Flash and Shine), Polar (A300, A360, Active, Loop, M600 and V800), Samsung (Gear 2, Gear S, Gear S2 and Gear S3), Withings (Pulse, Pulse 02 and Pulse Ox), and Xiaomi (Mi Brand and Mi Brand 2)
Georgiou et al. (2018) [39]		✓										Garmin (Forerunner 935, Premium HRM, Vivoactive HR + , 920XT, 910XT and HRM Tri,), Polar (H10 and H7), Wahoo Tickr HR Monitor, Whoop Strap (Strap 2.0), 4IIII (VIIIIVA), 60beat (HR Monitor), BlueLeza (HRM Blue), Cardiosport (TP3), Carre Technologies (Hexoskin), Empatica (E4 Wristband), Cositea (R2 Smart Fitness HRM Wristband), Mad Apparel (Athos), Medronic (HxM Smart HR), Mio (Alpha 2), Qardio (QardioCore), Sony (SmartBand 2), Sunnto (Smart sensor, sport), Wahoo Fitness (Tickr HRM and Tickr X Workout Tracker)
Germini et al. (2022) [40]	✓					✓	✓	✓				Apple Watch (series not specified), Garmin (Vivofit, Forerunner 225, Vivoactive HR, Vivofit 2, Forerunner 235, Vivosmart HR and Forerunner 920XT), Polar (Loop, V800 and A300), Fitbit (Flex, Alta, Charge HR, Charge, Ultra, One, Zip and Blaze), Jawbone (UP24 and UP2), Huawei B1, Xiaomi Mi Brand 1 and 2, Withhings Pulse, Basis Peak and Peak K
Giebel & Gissel (2019) [41]			✓									Apple Watch (Series 1), Fitbit (Blaze) and Polar (H7)
Haghayegh et al. (2019) [42]										✓	✓	Fitbit (Surge, Charge HR, Flex, Alta HR, Charge 2, One, Ultra, Classic and Versa)
Henriksen et al. (2020) [43]						✓	✓	✓				Polar (V800, A360, A300, M600, M430 and Loop)
Hermans et al. (2021) [44]			✓									Fitbit (Charge HR), Apple Watch (Series 3, A1554, Watch 4,) Xiaomi (Amazfit Health Band 1S), Empatica (E4), Wavelet Health, Samsung (Gear Fit 2), Honor (Band 4, Watch), Huawei (Watch GT) and Polar (H7)
Irwin and Gary (2022) [45]	✓					✓						Fitbit Charge 2
Kenyon et al. (2013) [46]						✓						Yamax Dig-Walker SW (200, 700 and 401)
Koerber et al. (2022) [47]	✓		✓									Apple, Fitbit, Mio Alpha and Garmin (models not provided and therefore grouped together on the basis of manufacturer)
Leung et al. (2021) [48]								✓				Fitbit (One, Zip, Flex, Blaze, Charge HR, Classic, Charge 2, Ultra, Alta, Alta HR and Surge)
Lopez Perales et al. (2021) [49]			✓									Huawei (Band 2 and Watch GT), Empatica (E4), Honor (Band 4, Watch) and Wavelet Health (Amiigo),
Molina Garcia et al. (2022) [50]				✓								Garmin (Fenix 5X, Fenix 3 + chest HR strap, Forerunner 920XT, GF5), Polar (A300, S410, F11, FT40, F6, RS300X and two V800) and Fitbit (Charge 2)
Nazarian et al. (2021) [51]			✓									Apple (NR), Huawei (Watch GT, The Honor Watch and The Honor Band), Huami (Amazfit Health Band IS), Samsung (Simband 2, GearFit and Gear S3), Empatica (E4) and Wavelet wristband
O’Driscoll et al. (2018) [52]								✓				Apple (Watch and Watch 2), Basis (B1 and Peak), Beurer (AS80), Epson (Pulsense), ePulse (Personal Fitness Assistant), Fitbit (Blaze, Charge, Charge 2, Charge HR, Flex and Surge), Garmin (Forerunner 225, Forerunner 920XT, Vivoactive, vivofit, vivosmart and vivosmart HR), Jawbone (UP and UP24), LifeCheck (Calorie sensor), Microsoft (Band), Mio (Alpha), Misfit (Shine), Nike (Fuel Band), Polar (Loop, AW200 and AW360), Samsung (Gear S), SenseWear (Armband, Mini, Pro 2 and Pro 3), TomTom (Touch), Vivago (Vivago) and Withings (Pulse and Pulse 02)
Schyvens et al. (2024) [53]										✓	✓	Fitbit Charge 4, Garmin Vivosmart 4 and WHOOP
Windisch et al. (2023) [54]					✓							Apple Series 6
Zhang et al. (2020) [55]	✓											Empatica, Fitbit, Apple, Garmin, Mio, TomTom, Basis Peak, Wavelet, PulseOn, Polar, Samsung, Tempo, Philips, Omron and Microsoft (models not reported)

*EE* energy expenditure, *HR* heart rate, *HRV* heart rate variability, *PA* physical activity, *SpO*_*2*_ blood oxygen saturation, *VO*_*2max*_, maximal aerobic capacity

### Validity of Wearables for Cardiovascular Health Biometrics

#### Heart Rate

Six systematic reviews (including two meta-analyses) assessed the validity of wearables for heart rate measurements [15, 36, 40, 45, 47, 55]. These reviews included 165 non-duplicate studies of 5816 participants (~ 50% female; Supplemental file 3). Only one review was deemed to be of high quality, with two reviews deemed to be of moderate quality and three of low quality. However the high-quality review by Chevance et al. focused specifically on the accuracy of one device manufacturer—Fitbit—in comparison to the reference standard, so the findings of the moderate quality reviews are also summarised below. The reference standard used for the measurement of heart rate included electrocardiograms (ECG), polar chest straps or pulse oximetry.

In the 52 studies included in the review by Chevance et al. [36], the pooled estimate of the meta-analysis showed a mean bias of − 3.39 bpm, indicating an underestimation of Fitbit devices for measuring heart rate compared with criterion measures. Subgroup analyses by population characteristics, intensities and types of activities and device models were consistent with this; however, the results of the subgroup meta-analyses for different intensities and types of activities revealed an underestimation of heart rate for cycling activities compared with daily living and treadmill activities as well as overground walking [36]. Measurement accuracy was also better for treadmills than for overground walking at moderate to vigorous intensity activities compared with light-intensity activities [36].

Neither of the reviews deemed to be of moderate quality performed a meta-analysis [15, 45]. In a narrative synthesis of 29 studies, Fuller et al. found that wrist-worn wearables typically exhibit measurement errors of approximately ± 3% regardless of the device (including those manufactured by Apple, Fitbit and Garmin) or criterion used [15]. The review by Irwin and Gary focused specifically on the Fitbit Charge 2 devices; five of the eight studies included in this review reported mean absolute percent error values of < 10% [45].

#### Heart Rate Variability

Two systematic reviews, one deemed to be of moderate quality and one of low quality, assessed the validity of wearables for measuring heart rate variability [34, 39]. Collectively, these reviews included 22 non-duplicate studies of 714 participants (~ 32% female; Supplemental File 3). Neither undertook a meta-analysis. Both reviews identified 2-lead, 3-lead, 5-lead and 12-lead ECG recordings as suitable criterion measures for the measurement of heart rate variability.

The review by Board et al. was deemed to be of moderate quality and included 13 studies, solely of Polar devices, finding “near perfect validity” (ICC 0.98–1.00) for temporal and spectral power heart rate variability (HRV) measures computed from inter-beat interval data when measurements were taken at rest [34]. In contrast, the review deemed to be of low quality by Georgiou et al. [39] evaluated a range of devices during both rest and exercise across 18 studies. Again, agreement between the indices of HRV was very good to excellent (ICCs ranging from 0.85 to 0.99) when measurements were taken at rest, however, this decreased to 0.85 as the level of exercise and/or motion increased.

#### Arrhythmia Detection

Six systematic reviews (two meta-analyses), two deemed to be of moderate quality and four of low quality, assessed the validity of wearables for measuring cardiac arrhythmia (including atrial fibrillation) [33, 41, 44, 47, 49, 51]. Collectively, these reviews included 85 non-duplicate studies of 877,127 participants (~ 42% female; Supplemental File 3). The reference standard for arrhythmia detection in these reviews included a 12-lead ECG, a Holter monitor, an ECG patch, telemetry, or an internet-enabled mobile ECG.

The meta-analysis by Nazarian et al. [51] measured diagnostic accuracy using smartwatches in 424,371 subjects across 18 studies. The Apple watch was used in seven studies, Samsung smartwatches were used in five studies and the remaining studies used a Huawei, Huami or Empatica smartwatch. Wearables demonstrated a pooled sensitivity of 100% (95% CI 0.99–1.00) and a pooled specificity of 95% (95% CI 0.93–0.97) for detecting cardiac arrhythmias in the sample populations. The pooled accuracy for arrhythmia detection was 97% (95% CI 0.96–0.99).

The other moderate quality systematic review by Lopez Perales et al. [49] evaluated several kinds of mobile health applications for detecting atrial fibrillation. Of the 11 included studies that evaluated a wearable device (including smartwatches and smart bands), diagnostic accuracy varied depending on the different algorithms utilised, the populations studied and the testing conditions. Smartwatches showed a sensitivity of 67.7–100% and a specificity of 67.6–98%, while smart bands showed a sensitivity of 75.4–97% and a specificity of 94–100% [49].

#### Aerobic Capacity

One high-quality systematic review and meta-analysis assessed the validity of wearables for measuring aerobic capacity (or VO_2max_) [50], the criterion measure for which was a graded exercise test to exhaustion with direct or indirect calorimetry. This review included 14 studies of 403 participants (45% female).

The results of the meta-analysis by Molina-Garcia et al. [50] showed that wearables using a resting test significantly overestimated VO_2max_ (bias = 2.17 ml kg^−1^ min^−1^; 95% CI 0.28–4.07). Conversely, wearables estimating VO_2max_ through exercise tests showed a bias close to nil compared with the reference standard (bias =  − 0.09 ml kg^−1^ min^−1^; 95% CI − 1.66 to 1.48). The limits of agreements in the resting test spanned from − 13.07 to 17.41 ml kg^−1^ min^−1^ (i.e. ± 15.24; 95% CI − 22.18 to 26.53), while limits were narrower in exercise testing conditions, spanning from − 9.92 to 9.74 ml kg^−1^ min^−1^ (i.e. ± 9.83; 95% CI − 16.79 to 16.61).

#### Blood Oxygen Saturation

One low-quality systematic review and meta-analysis assessed the validity of wearables for measuring blood oxygen saturation [54]. This review did not stipulate a criterion ‘ground truth’ criterion measure; however, of the five studies of 973 participants (44% female) that were included, the criterion measures were typically a pulse oximeter. None of the included studies reported the sex distribution in their respective cohorts. Each of the five included studies only evaluated the accuracy of the Apple Watch (series 6) against their chosen criterion.

The results showed that the Apple Watch Series 6 generally had a moderate to strong correlation with conventional pulse oximeters in measuring blood oxygen saturation, with Pearson correlation coefficients ranging from 0.76 to 0.89 in various studies. The mean absolute differences in oxygen saturation (SpO_2_) measurements between the Apple Watch and conventional oximeters ranged up to 2.0%. However, limits of agreement varied, with some studies showing ranges of − 5.8% to + 5.9%.

### Validity of Wearables for Measuring Physical Activity

#### Step Counting

Eight systematic reviews (including one meta-analysis) assessed the validity of wearables for counting steps [15, 36–38, 40, 43, 45, 46]. Collectively, these reviews included 184 non-duplicate studies of 6197 participants (~ 53% female; Supplemental File 3). Only one review was deemed to be of high quality, with three reviews deemed to be of moderate quality and four of low quality. As previously mentioned, the high-quality review by Chevance et al. [36] focused specifically on the accuracy of Fitbit devices, so the findings of the moderate quality reviews are also summarised below. The criterion measure for counting steps included manual counting (either in controlled settings or through video recording) or using accelerometers and pedometers (in free-living settings).

Chevance et al. [36] found in their analysis of 15 studies on Fitbit devices that most either reported underestimations or were inconclusive; the mean bias, when excluding inferior quality studies, was − 3.11 steps per minute (range − 13 to 7).

None of the reviews deemed to be of moderate quality performed a meta-analysis [15, 38, 45]. The review by Irwin and Gary focused specifically on the Fitbit Charge 2 device; they observed mean absolute percent error values of 12% [45]. Feehan et al. [38] also focused on Fitbit devices, which tended to underestimated counts by − 9% (median error =  − 3%). Fuller et al. found [15] that wrist-worn wearables typically underestimated step count (mean: − 9%, median: − 2%), with Withings and Misfit wearables consistently underestimating step count, and Apple and Samsung demonstrating less measurement variability than other brands.

#### Wheelchair Push Counts

One systematic review by Byrne et al. [35] that was deemed to be of low quality investigated the accuracy of wheelchair push counts measured by various fitness watches. This review included seven studies involving 131 wheelchair users (both able-bodied and those with disabilities; 40% female) aged over 18 years. The criterion measure used was direct observation in controlled laboratory settings. This method involved directly counting the wheelchair pushes and rotations to validate the accuracy of the wearable devices’ measurements.

The devices evaluated included various generations of the Apple Watch (Series 1 through 4), Garmin VivoFit, Fitbit Flex, Fitbit Flex 2, Jawbone UP24, and the Activ8 activity monitor. Among these, the Apple Watch Series 4 demonstrated the highest accuracy, with a mean absolute percentage error (MAPE) of 9.20%, compared with the Apple Watch Series 1 with a MAPE of 20.62%. The calibrated Apple Watch had a MAPE of 13.9%, whereas the uncalibrated version showed a higher MAPE of 22.8%. The Fitbit Flex 2 had the highest MAPE at 148.4%.

#### Physical Activity Intensity

Four systematic reviews (none of which included meta-analysis), one deemed to be of moderate quality and three of low quality, assessed the validity of wearables for measuring physical activity intensity [37, 38, 40, 43]. However the moderate quality review by Feehan et al. [38] focused specifically on the accuracy of one device manufacturer—Fitbit—in comparison to the reference standard so the findings of the low quality reviews are also summarised below.

Collectively, these reviews included 126 non-duplicate studies of 4087 participants (~ 55% female; Supplemental File 3). Their validation, primarily against the reference standard of research-grade accelerometers (e.g. Actigraph), revealed mixed results. Specifically, correlations with accelerometers varied considerably, and were influenced by elements such as activity intensity and duration. For Fitbit devices, the measurement error was less than − 10% for sedentary time, but over 80% of comparisons showed a measurement error greater than 10% for time spent in light to vigorous activity. In some cases, Fitbit devices tended to overcount minutes of moderate-to-vigorous physical activity, with mean absolute differences reaching up to 89.8 min per day. Other devices (e.g. Polar) showed varied performance, with some models offering strong correlations for light to vigorous physical activity intensities, but generally presenting poor agreement with reference devices, and mean absolute percentage errors ranging from 29 to 80% [37].

#### Energy Expenditure

Eight systematic reviews (three meta-analyses), one deemed to be of high quality, four of moderate quality and three of low quality, assessed the validity of wearables for measuring energy expenditure [15, 36–38, 40, 43, 48, 52]. Collectively, these reviews included 218 non-duplicate studies of 7734 participants (~ 54% female; Supplemental File 3). The reference standard for energy expenditure in these reviews included the doubly labelled water method, indirect and direct calorimetry in controlled (laboratory) environments and accelerometry in free-living settings. Again, the single high-quality review by Chevance et al. focused specifically on the accuracy of Fitbit devices [36], but so too did the moderate quality reviews by Feehan et al. [38] and Leung et al. [48].

Collectively, these reviews showed that wearables (Fitbit devices specifically) tended to underestimate energy expenditure by approximately 3 kcal per min (limits of agreement − 13 to 7 kcal per min) [36] or by − 3% [38].

In the systematic review and meta-analysis of 64 studies by O’Driscoll et al. [52], findings for a more diverse collection of devices showed that, again, wearables tended to underestimate energy expenditure [effect size (ES): − 0.23, 95% CI − 0.44 to − 0.03; *n* = 104; *p* = 0.03], with error in primary research studies ranging from − 21.27 to 14.76%. However, sensitivity analysis revealed that, upon the removal of specific devices from the analysis, the comparison to the criterion measured became non-significant (i.e. select wearables were highly accurate). Study heterogeneity was significant though, and the authors urged caution when interpreting their results: “while it is initially encouraging that the effect size for many devices was not significantly different from criterion, the 95% CI observed in many cases indicates the potential for these devices to produce erroneous estimates of mean energy expenditure and as such we would be hesitant to consider any device sufficiently accurate.”

### Validity of Wearables for Measuring Sleep

Four systematic reviews (including one meta-analysis), two deemed to be of moderate quality [38, 42] and two of low quality, assessed the validity of wearables for measuring various aspects of sleep [37, 53]. Collectively, these reviews included 95 non-duplicate studies of 2985 participants (~ 54% female; Supplemental File 3). The criterion measure used in these reviews for sleep was polysomnography.

Evenson et al. [37] in their review of four studies involving 180 participants evaluating the accuracy with which wearables could measure total sleep time (TST) or wakefulness, noted that consumer wearables tended to overestimate TST, while concurrently underestimating the time of wakefulness after sleep onset (mean absolute difference of approximately 22.0 min/day and ICC 0.85). Haghayegh et al. [42] derived their conclusions from 22 studies of 438 participants, which comprised both regular sleepers and individuals with diagnosed sleep disorders. The collective output from these studies was that wearables predominantly overestimated TST [42]. Eight studies within this review reported significant overestimations, and two posited non-significant overestimations compared with various aspects of sleep [42]. In contrast, these devices had a consistent trend of underestimating wakefulness after sleep onset. Five studies underscored significant underestimations, with another suggesting a non-significant underestimation. However, there were instances (based on three studies), where the readings between wearables and polysomnography (PSG) for wake time after sleep onset did not exhibit substantial disparities.

For sleep efficiency and onset latency, Haghayegh et al. [42] further reported on sleep efficiency, with a study indicating wearables significantly overestimating it against PSG. Sleep onset latency, the duration taken to transition from full wakefulness to sleep, was uniformly underestimated by wearables. Their review found three studies supporting this observation, with one highlighting a significant difference. Feehan et al. [38] specifically evaluated Fitbit devices under controlled settings. Their conclusions, derived from three studies, reiterated the patterns seen elsewhere: Fitbits typically overestimated TST and sleep efficiency, often by more than 10%. Their exploration into sleep-onset latency and wakefulness after sleep onset underscored vast inconsistencies, with measurement errors swinging between 12 and 180% [38].

### Meta-analysis

A meta-analysis of the individual studies was not undertaken as part of this umbrella review due to the heterogeneity in protocols, criterion measures, devices (including firmware versions) and outcomes evaluated. Specifically, of the 249 studies that validated at least one consumer wearable device for assessing at least one biometric outcome, only 166 used an accepted gold standard criterion. Of the studies that used an accepted gold standard, only 75 used appropriate statistical analysis [27] to determine device accuracy. Consequently, in all cases, for each biometric outcome there were fewer than five studies evaluating the same device for the same biometric outcome that used an accepted reference standard and appropriate statistical analysis. This precluded meta-analysis.

Going forward, subject to a sufficiently homogeneous body of literature emerging for specific devices and outcomes following recommended protocols [25–27], we will endeavour to undertake a meta-analysis of individual research studies as part of our ‘living’ research synthesis.

## Discussion

This umbrella review aimed to synthesise evidence from systematic reviews evaluating the validity of consumer wearable technologies for measuring biometric outcomes such as heart rate, aerobic capacity, energy expenditure, sleep and surrogate measures of physical activity. A total of 24 systematic reviews were deemed eligible for inclusion. Upon removal of duplicated studies within reviews, the net unique studies was adjusted to 391. These studies collectively included 888,033 participants (approximately 42% female).

For biometrics related to the cardiovascular system, our review revealed a subfield of research where accuracy varied on the basis of the specific measure assessed and the context in which the wearables were utilised. Heart rate measurements for example were associated with error rates of approximately − 3.39 bpm [36], or ± 3% [15], contingent upon user characteristics (e.g. skin tone), intensities of exercise and types of activities and device models [15, 36]. In contrast, the available research evaluating heart rate variability showed a high level of accuracy in rest measurements, with strong agreement with criterion measures, but this deteriorated during periods of physical exercise or motion [34, 39]. For arrhythmia detection, research primarily on devices manufactured by Apple and Samsung demonstrated good sensitivity and specificity [51], although it is important to note that the participants included in these studies were generally derived from clinical populations who had a prior diagnosis of cardiac arrhythmias such as atrial fibrillation. For aerobic capacity, a powerful measure of overall mortality risk [56, 57], the review conducted by the INTERLIVE consortium noted a tendency for wearables to overestimate VO_2max_ during resting tests but found them to be more accurate during exercise tests [50]. Finally, when measuring blood oxygen saturation, the mean absolute differences in SpO_2_ measurements taken from a wearable device (specifically, the Apple Watch series 6) and the criterion of pulse oximetry were up to 2.0% [54].

In the realm of physical activity, there was a trend for wearables to underestimate step counts, with disparities observed across various brands and models [15, 36]. Regarding the estimation of time spent in different physical activity intensity zones, there was marked inconsistency, with high measurement errors influenced by the type and intensity of activities [37]. Energy expenditure assessments by wearables seem to veer towards underestimation, with a significant range of error margins and device-specific accuracy nuances [36, 38, 48, 52].

Finally, in relation to sleep, wearable devices showed a consistent trend of overestimating total sleep time (TST) and sleep efficiency. For example, wearables typically overestimated TST by more than 10%, and sleep efficiency similarly showed overestimations in multiple studies. Additionally, wearables tended to underestimate sleep onset latency and wakefulness after sleep onset, with errors ranging from 12 to 180% [37, 38, 42]. These findings indicate significant disparities when benchmarked against polysomnography standards, highlighting the need for further refinement and validation of sleep tracking features in consumer wearables.

Taken at face value, our results would suggest that consumer wearables appear moderately proficient in capturing various health outcomes such as heart rate, heart rate variability, aerobic capacity and others. However, this does not capture the nuance of the current state of wearable technology research. To explore the current research landscape, we collated a list of 310 consumer wearables spanning various manufacturers. Of these devices, only 34 (11%) have been validated for at least one biometric outcome in the primary research studies included in our review. Given that most consumer wearables can measure multiple biometric outcomes, the potential number of discrete validation studies required is substantial. Specifically, if each device were validated for five outcomes (step count, heart rate, sleep, physical activity and energy expenditure), this list would necessitate 1550 individual validation studies. However, only 54 validation studies (3.5% of the potential total) have been conducted to date, highlighting a significant gap in the existing evidence landscape. This highlights the disparity between the number of wearable devices on the market and those that have undergone validation—an issue that is widely acknowledged among researchers [16]. This gap underscores the challenges of conducting validation research that keeps pace with the extremely agile commercial ecosystem. The field has attempted to stay current with a multitude of devices, which often have annual release cycles and use diverse methodologies, leading to significant heterogeneity in research outcomes [16].

Thus, our findings do not conclusively indicate that wearables consistently underestimate heart rate, overestimate sleep time or fail to accurately measure energy expenditure (for example). Instead, this umbrella review reveals the intricate variability across devices, outcomes, user contexts and reference standards, making a definitive assessment of wearables’ accuracy challenging. The pervasive heterogeneity in research methodologies and findings limits the practical applicability of these technologies, highlighting the urgent need for standardised validation protocols that are both rigorous and adaptable to the rapidly evolving wearable technology landscape. This need for standardisation is evident from the fact that only 71% of the primary research studies included in the reviews utilised an accepted gold standard criterion, and only 40% of these studies followed best practice statistical analysis [27] to determine device accuracy. Developing robust frameworks and fostering collaborative partnerships with industry are essential to enhance the reliability, consistency, and scientific integrity of wearable technology assessments [16]. Therefore, the question of wearables’ accuracy remains inherently indeterminate, influenced by device-specific, outcome-centric, user-related and contextual variables, making a decisive answer elusive based on the currently available literature.

One of the cardinal challenges encountered in reviewing the literature in this field is the swift and continuous evolution of the commercial landscape for wearable technologies. Given this rapid advancement, the research captured within our review inevitably serves as a historical snapshot, reflecting the accuracy and validity of devices as they existed approximately 2 years prior to the date the search was conducted. This temporal disconnect is exemplified by the chronology of our reference materials—only one of the included reviews was published in 2024 [53], with most being published in 2022 [36, 40, 44, 45, 47, 48, 50]—and the most recent primary study included therein was published in August 2022 [58]. Illustrative of the ongoing output of the commercial engine, consider that, as at the time of writing (June 2024), the market has seen the release of three new iterations of Apple Watch alone since the most recent primary research study included in our results. A universal observation across the wearables reviewed in this research synthesis is their transient market presence; each device analysed has since either been retired or superseded by a more recent model. Despite a semblance of hardware continuity in newer models, the frequent deployment of updated firmware and algorithms can profoundly impact device performance and measurement accuracy. This illustrates a fundamental tension: the measured and methodical pace of rigorous academic inquiry versus the agile, ever-fluxing dynamism inherent in the commercial technology ecosystem. Consequently, our findings, while insightful, may not fully mirror the current state of wearable technology capabilities.

This underscores the importance of making this a ‘living’ research synthesis that evolves concurrently with ongoing technological advancements and refinements. In the realm of wearable technology validation, there exists a kind of ‘validation economy’ marked by diverse, simultaneous efforts aimed at assessing and ensuring device accuracy and reliability. At one end of the spectrum, the popular media landscape is populated by vloggers and influencers who wield substantial reach and influence. Their ‘reviews’—often reactionary, anecdotal and based on single-subject analyses—command vast audiences, albeit with intrinsic methodological limitations. For instance, a review by Marques Brownlee amassed 3 million views within the first 3 weeks of the latest Apple Watch release, reflecting the potent sway of such platforms despite their often informal and experiential evaluation methods [59]. In parallel, more structured and formalised efforts are underway within academic and professional circles to cultivate rigorous validation frameworks. Initiatives such as INTERLIVE spearhead these endeavours by advocating for standardised protocols and best practices in evaluating wearable technologies [25–27]. Through its collaborative network, INTERLIVE strives to formalise and standardise many aspects of validity assessment for consumer-grade wearables, towards the development of recommendations and guidelines that bolster the utility and reliability of wearable-derived data in capturing physical activity indicators. In addition, organisations such as FIMS have instituted quality assurance standards to scrutinise and verify the marketing claims of wearable device companies [24]. Their approach envisions a proactive validation process, wherein manufacturers engage in a pre-market validation exercise, submitting their devices for rigorous evaluation against established research benchmarks or appropriate proxies [24]. Meanwhile, vast data repositories are being cultivated through national initiatives such as the All of Us program from the National Institutes of Health (NIH) [60] and the UK Biobank [61]. These databanks harvest extensive, longitudinal health data from wearable devices, fostering a richer, more nuanced understanding of various health conditions and the role of wearable technologies in managing them. Private enterprises, too, are making significant strides, specialising in harnessing and analysing wearable-derived data to bolster both individual and corporate wellness endeavours [62–65].

In this context, we hope that this living umbrella review will serve to synthesise and harmonise the disparate threads of research and evaluation emanating from various quarters. While its primary focus is to provide researchers with a consolidated and continuously updated synthesis of the latest evidence, we recognise that our readership will likely extend beyond the traditional academic audience. This includes healthcare professionals, policy makers, technology developers and an informed public interested in the accuracy and reliability of wearable technologies. Given the broad and diverse interest in wearable technology, our dissemination strategy aims to maximise the impact of our findings across multiple stakeholder groups. Our public engagement activities will include dissemination of these findings via personal and institutional social media, on our YouTube channel [66] and in specific undergraduate and postgraduate modules in digital health and medical devices. We will involve patient and public representatives in creating a plain language summary of findings to be distributed to the general population, informing policy makers across different countries with written communications [16]. By adopting this approach, our review seeks to become a resource and ally for a multitude of stakeholders. For the vloggers and influencers navigating the vibrant but volatile landscape of new device releases and software updates, it will offer a repository of up-to-date research findings. For formal validation bodies and research consortiums such as INTERLIVE and FIMS, it will provide a cohesive and continuous synthesis of global research efforts, bolstering their frameworks and recommendations with a broader perspective and the latest insights. End-users, too, stand to gain from this living umbrella review. With wearables becoming more ingrained in society [67], and as their user base expands and diversifies, the review will be a valuable resource to help users determine the accuracy and reliability of various devices. It is anticipated that the value of the review will grow as the pace of research accelerates and wearable devices permeate deeper into everyday health management and lifestyle practices.

Yet, despite its strengths and potential value, this review is not without limitations. As previously mentioned, an intrinsic challenge lies in the temporal incongruence between the fast-paced evolution of consumer wearable technologies and the more deliberate and methodical pace of academic research and publishing. Due to the procedural necessities of academic rigor—protocol development, study implementation, data analysis and the subsequent publication processes—our synthesis inherently lags behind the latest commercial innovations and releases [16]. Second, our review operates primarily as a tertiary research synthesis, grounding its insights in secondary research—systematic reviews of primary studies. This methodology, while offering a powerful and practical way of collating and synthesising large amounts of data, introduces vulnerability to potential biases or errors that might pervade the secondary research layers—conclusions drawn herein are reflections of the interpretations, methodologies and potential biases of the underlying reviews and their authors. Indeed, our effort to mitigate these limitations manifested in our risk of bias assessment, which showed that the 24 systematic reviews deemed eligible for inclusion rarely met all of the established criteria. This underscores the need for cautious interpretation and application of our findings. Ultimately, this umbrella review should first be considered a directory to research in the field and, second, a high-level overview of results. The variability that pervades various dimensions of the incorporated studies—including their chosen protocols, selected devices, criterion measures, demographic considerations and statistical methodologies—and the nested structure of conclusions necessitate a cautious approach in extracting coherent and reliable signals amidst the noise of disparate findings [68].

## Conclusion

In conclusion, this umbrella review illuminates the varied landscape in consumer wearable technology research, showcasing the potential and complexity inherent in their validation and utilisation. Our findings also highlight the need to formalise and standardise device- and biometric-specific validation protocols, and the potential benefit of an agile research model according to which new devices can be evaluated. Furthermore, fostering collaborative synergies between formal certification bodies, academic research consortia, popular media influencers and industry could augment the depth, reach and inclusivity of wearable technology evaluations, enabling a richer, multifaceted dialogue that resonates with a broad spectrum of stakeholders. Opportunities also lie in the expansion of validity assessments into diverse and unexplored terrains of wearable utility, such as stress, training readiness and ‘body battery’ scores; wearable technology companies such as Fitbit, Garmin, Oura and Whoop have each created variants of these ‘bespoke biometrics’ which collate multiple biometric signals to give a user an idea of their current health status; however, none have undergone formal validation. Crucially, as wearable technologies burgeon, penetrating various facets of health and lifestyle, a continued commitment to ethical considerations, data privacy and user autonomy remains imperative. The pursuit of enhancing accuracy and reliability should unfold alongside endeavours to nurture an ecosystem of ethical technology use, marked by transparency, user empowerment and a conscientious alignment with overarching health and societal objectives.

## Supplementary Information

Below is the link to the electronic supplementary material.

Supplementary file1 (DOCX 27 KB)

Supplementary file2 (DOCX 494 KB)

## Declarations

### Transparency Statement

The authors declare the manuscript is an honest, accurate and transparent account of the study being reported; no important aspects of the study have been omitted; and any discrepancies from the study as originally planned have been explained in published protocol and supplementary methods.

### Data Availability

Data for this living systematic review will be made available on Open Science Framework (OSF; https://osf.io/fqvms/?view_only=e49e7c42dfd3475db5cf2da3b15e4b3f).

## References

[1] Lupton D. The quantified self: a sociology of self-tracking. Sociol Health Illn. 2016;39:1557–71. 10.1111/1467-9566.12617 29071731

[2] Piwek L, Ellis DA, Andrews S, Joinson A. The rise of consumer health wearables: promises and barriers. PLoS Med. 2016;13(2): e1001953. 26836780 10.1371/journal.pmed.1001953 PMC4737495

[3] GVR. Wearable technology market size, share & trends analysis report by product (head & eyewear, wristwear), by application (consumer electronics, healthcare), by region (Asia Pacific, Europe), And Segment Forecasts, 2023–2030: Grand View Research; 2023. Report No.: 978-1-68038-165-8.

[4] ACSM. Wearable technology named top fitness trend for 2024. 2024 [cited; https://www.acsm.org/education-resources/trending-topics-resources/acsm-fitness-trends.

[5] Carpenter A, Frontera A. Smart-watches: a potential challenger to the implantable loop recorder? Europace. 2016;18(6):791–3. 26847074 10.1093/europace/euv427

[6] Jia Y, Wang W, Wen D, Liang L, Gao L, Lei J. Perceived user preferences and usability evaluation of mainstream wearable devices for health monitoring. PeerJ. 2018;6: e5350. 30065893 10.7717/peerj.5350 PMC6064199

[7] Hickey AM, Freedson PS. Utility of consumer physical activity trackers as an intervention tool in cardiovascular disease prevention and treatment. Prog Cardiovasc Dis. 2016;58(6):613–9. 26943981 10.1016/j.pcad.2016.02.006

[8] Radin JM, Wineinger NE, Topol EJ, Steinhubl SR. Harnessing wearable device data to improve state-level real-time surveillance of influenza-like illness in the USA: a population-based study. Lancet Digit Health. 2020;2(2):e85–93. 33334565 10.1016/S2589-7500(19)30222-5 PMC8048388

[9] Perez MV, Mahaffey KW, Hedlin H, Rumsfeld JS, Garcia A, Ferris T, et al. Large-scale assessment of a smartwatch to identify atrial fibrillation. N Engl J Med. 2019;381(20):1909–17. 31722151 10.1056/NEJMoa1901183 PMC8112605

[10] Kimura N, Aso Y, Yabuuchi K, Ishibashi M, Hori D, Sasaki Y, et al. Association of modifiable lifestyle factors with cortical amyloid burden and cerebral glucose metabolism in older adults with mild cognitive impairment. JAMA Netw Open. 2020;3(6): e205719. 32515796 10.1001/jamanetworkopen.2020.5719 PMC7284299

[11] Shilaih M, Goodale BM, Falco L, Kübler F, De Clerck V, Leeners B. Modern fertility awareness methods: wrist wearables capture the changes in temperature associated with the menstrual cycle. Biosci Rep. 2018;38(6): BSR20171279. 29175999 10.1042/BSR20171279 PMC6265623

[12] clinicaltrials.gov. Clinicaltrials.gov search results. 2023 [cited; https://clinicaltrials.gov/search?term=fitbit&aggFilters=status:rec.

[13] Shiffman S, Stone AA, Hufford MR. Ecological momentary assessment. Annu Rev Clin Psychol. 2008;4:1–32. 18509902 10.1146/annurev.clinpsy.3.022806.091415

[14] Zapata-Lamana R, Lalanza JF, Losilla JM, Parrado E, Capdevila L. mHealth technology for ecological momentary assessment in physical activity research: a systematic review. PeerJ. 2020;8: e8848. 32257648 10.7717/peerj.8848 PMC7103204

[15] Fuller D, Colwell E, Low J, Orychock K, Tobin MA, Simango B, et al. Reliability and validity of commercially available wearable devices for measuring steps, energy expenditure, and heart rate: systematic review. JMIR Mhealth Uhealth. 2020;8(9): e18694. 32897239 10.2196/18694 PMC7509623

[16] Keogh A, Argent R, Doherty C, Duignan C, Fennelly O, Purcell C, et al. Breaking down the digital fortress: the unseen challenges in healthcare technology—lessons learned from 10 years of research. Sensors. 2024;24(12):3780. 38931564 10.3390/s24123780 PMC11207951

[17] Huhn S, Axt M, Gunga H-C, Maggioni MA, Munga S, Obor D, et al. The impact of wearable technologies in health research: scoping review. JMIR Mhealth Uhealth. 2022;10(1): e34384. 35076409 10.2196/34384 PMC8826148

[18] Dunn J, Runge R, Snyder M. Wearables and the medical revolution. Per Med. 2018;15(5):429–48. 30259801 10.2217/pme-2018-0044 PMC12294383

[19] Gualtieri L, Rosenbluth S, Phillips J. Can a free wearable activity tracker change behavior? The impact of trackers on adults in a physician-led wellness group. JMIR Res Protoc. 2016;5(4): e237. 27903490 10.2196/resprot.6534 PMC5156822

[20] Montoye AHK, Mitrzyk JR, Molesky MJ. Comparative accuracy of a wrist-worn activity tracker and a smart shirt for physical activity assessment. Meas Phys Educ Exerc Sci. 2017;21(4):201–11.

[21] Evenson KR, Goto MM, Furberg RD. Systematic review of the validity and reliability of consumer-wearable activity trackers. Int J Behav Nutr Phys Activity. 2015;12(1):159. 10.1186/s12966-015-0314-1 PMC4683756 26684758

[22] Toth LP, Park S, Springer CM, Feyerabend MD, Steeves JA, Bassett DR. Video-recorded validation of wearable step counters under free-living conditions. Med Sci Sports Exerc. 2018;50(6):1315–22. 29381649 10.1249/MSS.0000000000001569

[23] Kim J, Campbell AS, de Ávila BE-F, Wang J. Wearable biosensors for healthcare monitoring. Nat Biotechnol. 2019;37(4):389–406. 30804534 10.1038/s41587-019-0045-y PMC8183422

[24] Ash GI, Stults-Kolehmainen M, Busa MA, Gaffey AE, Angeloudis K, Muniz-Pardos B, et al. Establishing a global standard for wearable devices in sport and exercise medicine: perspectives from academic and industry stakeholders. Sports Med. 2021;51(11):2237–50. 34468950 10.1007/s40279-021-01543-5 PMC8666971

[25] Argent R, Hetherington-Rauth M, Stang J, Tarp J, Ortega FB, Molina-Garcia P, et al. Recommendations for determining the validity of consumer wearables and smartphones for the estimation of energy expenditure: expert statement and checklist of the INTERLIVE network. Sports Med. 2022;52(8):1817–32. 35260991 10.1007/s40279-022-01665-4 PMC9325806

[26] Johnston W, Judice PB, Molina García P, Mühlen JM, Lykke Skovgaard E, Stang J, et al. Recommendations for determining the validity of consumer wearable and smartphone step count: expert statement and checklist of the INTERLIVE network. Br J Sports Med. 2021;55(14):780–93. 33361276 10.1136/bjsports-2020-103147 PMC8273687

[27] Mühlen JM, Stang J, Lykke Skovgaard E, Judice PB, Molina-Garcia P, Johnston W, et al. Recommendations for determining the validity of consumer wearable heart rate devices: expert statement and checklist of the INTERLIVE Network. Br J Sports Med. 2021;55(14):767–79. 33397674 10.1136/bjsports-2020-103148 PMC8273688

[28] Düking P, Stammel C, Sperlich B, Sutehall S, Muniz-Pardos B, Lima G, et al. Necessary steps to accelerate the integration of wearable sensors into recreation and competitive sports. Curr Sports Med Rep. 2018;17(6):178–82. 29889145 10.1249/JSR.0000000000000495

[29] Simmonds M, Elliott JH, Synnot A, Turner T. Living systematic reviews. Methods Mol Biol. 2022;2345:121–34. 34550587 10.1007/978-1-0716-1566-9_7

[30] Cochrane. Living systematic reviews. 2023 [cited; https://community.cochrane.org/review-development/resources/living-systematic-reviews#:~:text=Living%20Evidence%20Network-,What%20is%20a%20living%20systematic%20review%3F,evidence%20as%20it%20becomes%20available.

[31] Moher D, Liberati A, Tetzlaff J, Altman DG. Preferred reporting items for systematic reviews and meta-analyses: the PRISMA statement. Br Med J (Online). 2009;339(7716):332–6. PMC3090117 21603045

[32] Drucker AM, Fleming P, Chan AW. Research techniques made simple: assessing risk of bias in systematic reviews. J Invest Dermatol. 2016;136(11):e109–14. 27772550 10.1016/j.jid.2016.08.021

[33] Belani S, Wahood W, Hardigan P, Placzek AN, Ely S. Accuracy of detecting atrial fibrillation: a systematic review and meta-analysis of wrist-worn wearable technology. Cureus. 2021;13(12): e20362. 35036196 10.7759/cureus.20362 PMC8752409

[34] Board EM, Ispoglou T, Ingle L. Validity of telemetric-derived measures of heart rate variability: a systematic review. J Exerc Physiol Online. 2016;19(6):64–84.

[35] Byrne J, Lynch S, Shipp A, Tran B, Mohan S, Reindel K. Investigating the accuracy of wheelchair push counts measured by fitness watches: a systematic review. Cureus. 2023;15(9): e45322. 37849605 10.7759/cureus.45322 PMC10577091

[36] Chevance G, Golaszewski NM, Tipton E, Hekler EB, Buman M, Welk GJ, et al. Accuracy and precision of energy expenditure, heart rate, and steps measured by combined-sensing fitbits against reference measures: systematic review and meta-analysis. JMIR Mhealth Uhealth. 2022;10(4): e35626. 35416777 10.2196/35626 PMC9047731

[37] Evenson KR, Goto MM, Furberg RD. Systematic review of the validity and reliability of consumer-wearable activity trackers. Int J Behav Nutr Phys Act. 2015;12:1–22. 26684758 10.1186/s12966-015-0314-1 PMC4683756

[38] Feehan LM, Geldman J, Sayre EC, Park C, Ezzat AM, Yoo JY, et al. Accuracy of fitbit devices: systematic review and narrative syntheses of quantitative data. JMIR Mhealth Uhealth. 2018;6(8): e10527. 30093371 10.2196/10527 PMC6107736

[39] Georgiou K, Larentzakis AV, Khamis NN, Alsuhaibani GI, Alaska YA, Giallafos EJ. Can wearable devices accurately measure heart rate variability? A systematic review. Folia Med. 2018;60(1):7–20. 10.2478/folmed-2018-0012 29668452

[40] Germini F, Noronha N, Debono VB, Philip BA, Pete D, Navarro T, et al. Accuracy and acceptability of wrist-wearable activity-tracking devices: systematic review of the literature. J Med Internet Res. 2022;24(1): e30791. 35060915 10.2196/30791 PMC8817215

[41] Giebel GD, Gissel C. Accuracy of mHealth devices for atrial fibrillation screening: systematic review. JMIR Mhealth Uhealth. 2019;7(6): e13641. 31199337 10.2196/13641 PMC6598422

[42] Haghayegh S, Khoshnevis S, Smolensky MH, Diller KR, Castriotta RJ. Accuracy of wristband fitbit models in assessing sleep: systematic review and meta-analysis. J Med Internet Res. 2019;21(11): e16273. 31778122 10.2196/16273 PMC6908975

[43] Henriksen A, Johansson J, Hartvigsen G, Grimsgaard S, Hopstock L. Measuring physical activity using triaxial wrist worn polar activity trackers: a systematic review. Int J Exerc Sci. 2020;13(4):438–54. 32509122 10.70252/RPTD5267 PMC7241625

[44] Hermans ANL, Gawalko M, Dohmen L, van der Velden RMJ, Betz K, Duncker D, et al. Mobile health solutions for atrial fibrillation detection and management: a systematic review. Clin Res Cardiol. 2022;111(5):479–91. 34549333 10.1007/s00392-021-01941-9 PMC8454991

[45] Irwin C, Gary R. Systematic review of Fitbit Charge 2 validation studies for exercise tracking. Transl J Am Coll Sports Med. 2022;7(4):1–7. 36711436 10.1249/tjx.0000000000000215 PMC9881599

[46] Kenyon A, McEvoy M, Sprod J, Maher C. Validity of pedometers in people with physical disabilities: a systematic review. Arch Phys Med Rehabil. 2013;94(6):1161–70. 23201318 10.1016/j.apmr.2012.11.030

[47] Koerber D, Khan S, Shamsheri T, Kirubarajan A, Mehta S. Accuracy of heart rate measurement with wrist-worn wearable devices in various skin tones: a systematic review. J Racial Ethnic Health Disparities. 2022;10:2676–84. 10.1007/s40615-022-01446-9 PMC9662769 36376641

[48] Leung W, Case L, Sung MC, Jung J. A meta-analysis of Fitbit devices: same company, different models, different validity evidence. J Med Eng Technol. 2022;46(2):102–15. 34881682 10.1080/03091902.2021.2006350

[49] Lopez Perales CR, Van Spall HGC, Maeda S, Jimenez A, Laţcu DG, Milman A, et al. Mobile health applications for the detection of atrial fibrillation: a systematic review. Europace. 2021;23(1):11–28. 33043358 10.1093/europace/euaa139 PMC7842109

[50] Molina-Garcia P, Notbohm HL, Schumann M, Argent R, Hetherington-Rauth M, Stang J, et al. Validity of estimating the maximal oxygen consumption by consumer wearables: a systematic review with meta-analysis and expert statement of the INTERLIVE network. Sports Med. 2022;52(7):1577–97. 35072942 10.1007/s40279-021-01639-y PMC9213394

[51] Nazarian S, Lam K, Darzi A, Ashrafian H. Diagnostic accuracy of smartwatches for the detection of cardiac arrhythmia: systematic review and meta-analysis. J Med Internet Res. 2021;23(8): e28974. 34448706 10.2196/28974 PMC8433941

[52] O’Driscoll R, Turicchi J, Beaulieu K, Scott S, Matu J, Deighton K, et al. How well do activity monitors estimate energy expenditure? A systematic review and meta-analysis of the validity of current technologies. Br J Sports Med. 2020;54(6):332–40. 30194221 10.1136/bjsports-2018-099643

[53] Schyvens AM, Van Oost NC, Aerts JM, Masci F, Peters B, Neven A, et al. Accuracy of Fitbit Charge 4, Garmin Vivosmart 4, and WHOOP versus polysomnography: systematic review. JMIR Mhealth Uhealth. 2024;27(12): e52192. 10.2196/52192 PMC11004611 38557808

[54] Windisch P, Schröder C, Förster R, Cihoric N, Zwahlen DR. Accuracy of the Apple Watch oxygen saturation measurement in adults: a systematic review. Cureus. 2023;15(2): e35355. 36974257 10.7759/cureus.35355 PMC10039641

[55] Zhang Y, Weaver RG, Armstrong B, Burkart S, Zhang S, Beets MW. Validity of wrist-worn photoplethysmography devices to measure heart rate: a systematic review and meta-analysis. J Sports Sci. 2020;38(17):2021–34. 32552580 10.1080/02640414.2020.1767348

[56] Kokkinos P, Faselis C, Samuel IBH, Pittaras A, Doumas M, Murphy R, et al. Cardiorespiratory fitness and mortality risk across the spectra of age, race, and sex. J Am Coll Cardiol. 2022;80(6):598–609. 35926933 10.1016/j.jacc.2022.05.031

[57] Mandsager K, Harb S, Cremer P, Phelan D, Nissen SE, Jaber W. Association of cardiorespiratory fitness with long-term mortality among adults undergoing exercise treadmill testing. JAMA Netw Open. 2018;1(6): e183605. 30646252 10.1001/jamanetworkopen.2018.3605 PMC6324439

[58] Spaccarotella C, Polimeni A, Mancuso C, Pelaia G, Esposito G, Indolfi C. Assessment of non-invasive measurements of oxygen saturation and heart rate with an Apple Smartwatch: comparison with a standard pulse oximeter. J Clin Med. 2022;11(6):1467. 35329793 10.3390/jcm11061467 PMC8951323

[59] Brownlee M. Apple Watch Series 9 & Ultra 2: What Are We Waiting For?! 2023 [cited 13/10/23]; https://www.youtube.com/watch?v=oNCs4C2SMjo.

[60] Denny JC, Rutter JL, Goldstein DB, Philippakis A, Smoller JW, Jenkins G, et al. The “all of us” research program. N Engl J Med. 2019;381(7):668–76. 31412182 10.1056/NEJMsr1809937 PMC8291101

[61] Stamatakis E, Ahmadi MN, Gill JMR, Thøgersen-Ntoumani C, Gibala MJ, Doherty A, et al. Association of wearable device-measured vigorous intermittent lifestyle physical activity with mortality. Nat Med. 2022;28(12):2521–9. 36482104 10.1038/s41591-022-02100-x PMC9800274

[62] Thryve. 2023 [cited; Available from: https://thryve.health.

[63] Fitrockr. fitrockr health solutions. 2023.

[64] Fitabase. 2023.

[65] Labfront. 2023 [cited; Available from: https://www.labfront.com.

[66] Doherty C. YouTube Channel: @CailbheDoherty. 2024. https://www.youtube.com/channel/UCxet-B3eDU6naEo68SbnMMA.

[67] Statista. Statistics report on wearables; 2023.

[68] Belbasis L, Bellou V, Ioannidis JPA. Conducting umbrella reviews. BMJ Med. 2022;1(1): e000071. 36936579 10.1136/bmjmed-2021-000071 PMC9951359

