# Supplementary material for: Keeping Pace with Wearables: A Living Umbrella Review of Systematic Reviews Evaluating the Accuracy of Consumer Wearable Technologies in Health Measurement
Source: Sports Med. 2024 Jul 30;54(11):2907–26. doi: 10.1007/s40279-024-02077-2 (PMC11560992; doi:10.1007/s40279-024-02077-2)
Supplement: Supplementary file 2 — Supplementary file2 (DOCX 494 KB) [file 40279_2024_2077_MOESM2_ESM.docx]

**Supplemental File 2. The full characteristics of the dataset (including results and the authors’ conclusions).**

**Keeping Pace with Wearables: A Living Umbrella Review of Systematic Reviews Evaluating the Accuracy of Commercial Wearable Technologies in Health Measurement**

Cailbhe Doherty^1,2^, Maximus Baldwin^1,3^, Alison Keogh^2,4^, Brian Caulfield^1,2^, Rob Argent^2,4^

Affiliations:

1. School of Public Health, Physiotherapy and Sports Science, University College Dublin, Dublin, Ireland.
2. Insight Centre for Data Analytics, University College Dublin, Dublin, Ireland.
3. Institute for Sport and Health, University College Dublin, Dublin, Ireland
4. School of Medicine, Trinity College Dublin, Dublin, Ireland.
5. School of Pharmacy and Biomolecular Sciences, Royal College of Surgeons (RCSI), University of Medicine and Health Sciences, Dublin, Ireland.

**ORCiD**

Cailbhe Doherty: <https://orcid.org/0000-0002-5284-856X>

Maximus Baldwin: <https://orcid.org/0000-0002-0390-3956>

Alison Keogh: <https://orcid.org/0000-0001-5917-6308>

Brian Caulfield: <https://orcid.org/0000-0003-0290-9587>

Rob Argent: <https://orcid.org/0000-0002-3522-9409>

**Correspondence to:** C Doherty [cailbhe.doherty@ucd.ie](mailto:cailbhe.doherty@ucd.ie)

Supplemental Table 2. Table of characteristics of included systematic reviews and meta-analyses.

| DOI | Authors (year) | Health status | N subjects | Age mean or range, years (SD) | Biometric investigated | Criterion measure | Lab/controlled or free-living environment | Wearable brand (model) |
| --- | --- | --- | --- | --- | --- | --- | --- | --- |
| 10.7759/cureus.20362 | Belani et al (2021) | Patients that have been diagnosed with atrial fibrillation | 1561 | Mean age of 70 | Atrial fibrillation. | 12-lead ECG, 7-lead Holter monitor, ICM recording, Telemetry. | Lab/controlled | Apple (NR), Kardiaband (NR), Samsung (NR) |
| None | Board et al (2016) | Healthy | 595 | Mean age of participants was 33 (the range 21 to 57 years) | 1) Detecting inter-beat intervals and error associated, and 2) Heart rate variability readings | ECG (2, 3, 5, and 12-lead) | Lab/controlled | Polar (V800, RS800CX, 810) Suunto (T6) |
| 10.7759/cureus.45322 | Byrne et a (2023) | Wheelchair users (able bodied included) | 126 | NR | Wheelchair push counts | Direct observation | Lab/controlled and free-living | Apple Watches of different generations, Garmin VivoFit, Fitbit Flex, Fitbit Flex 2, and Jawbone UP24. |
| 10.2196/35626 | Chevance et al (2022) | Mostly healthy, some patients with some patient populations (cardiac and/or respiratory conditions, chronic pain, and Parkinson disease). | 1628 | Mean ages ranged from 7.2 to 64 | 1) Energy expenditure, 2) Heart rate, and 3) Step count | ***Energy expenditure***: Doubly labelled water, indirect and direct calorimetry, ***Heart rate***: ECG, pulse oximetry, and specific chest worn straps (Polar). ***Steps***: Direct observation (video recorded or not). | Lab/controlled and free-living | Fitbit (Charge HR, Charge 2, Blaze, Surge, Versa, Charge 3, Ionic) |
| 10.1186/s12966-015-0314-1 | Evenson et al (2015) | Younger and middle aged adults (with one study looking at elderly >60 years of age, and two studies looking at "youth") | 593* | Mean ages ranged from 9.7 (4.3) to 73 (6.9) | 1) Energy expenditure, 2) Step count, 3) Distance, 4) Sleep, 5)Physical activity | ***Steps***: Manual counting (controlled settings), accelerometers and pedometers (free-living settings). ***Energy expenditure***: indirect and direct calorimetry (controlled), accelerometers (ActiGraph and BodyMedia SenseWear) (free-living settings). ***Sleep***: polysomnography (PSG). **Distance:** Treadmill distance | Lab/controlled/free-living | Fitbit (Ultra, One, Zip, Flex, Force, Charge, Surge, Charge HR), and Jawbone ( UP, UP24, UP MOVE, UP2, UP3, UP4) |
| 10.2196/10527 | Feehan et al (2018) | Mostly health participants in the general population, but also included studies of participants with mobility limitations and/or chronic diseases. | 2441 | Mean age 37 (18) with a range of 21 to 84 years | 1) Step count, 2) Energy expenditure, 3) Sleep, 4)Time in activity | ***Step count (controlled setting)***: Direct observations and manual counting. ***Energy expenditure (controlled setting)***: Direct and indirect calorimetry, (free-living settings) doubly labelled water, and accelerometers. ***Sleep***: PSG over a 1 night sleep in a sleep laboratory. | Lab/controlled and free-living | Fitbit (Ultra, Classic, Zip, One, the Flex, Charge HR, Force, and, Surge) |
| 10.2196/18694 | Fuller et al (2020) | Mostly health participants in the general population, but also included studies of participants with mobility limitations and/or chronic diseases. | 5934 | Mean age of 36.8 (18.3), range 3.7-87 years | 1) Energy expenditure, 2) Heart rate, and 3) Step count | ***Step count (controlled setting)***: Manual counting and accelerometery. ***Heart rate (controlled setting)***: ECG, Polar chest straps, and pulse oximetry. ***Energy expenditure (controlled setting)***: Direct and indirect calorimetry. ***Step count( free-living settings)***: Accelerometery, ***Heart rate ( free-living settings)***: Polar chest strap. ***Energy expenditure ( free-living settings)***: Doubly labelled water (1 study) and accelerometery. | Lab/controlled and free-living | Apple Watch (Series 1 and 2), Fitbit (Alta, Blaze, Charge, Charge 2, Charge HR, Classic, Flex 1 and 2, Force, One, Surge, Ultra, Zip), Garmin (Fenix 3HR, Forerunner 225, Forerunner235, Forerunner 405CX, Forerunner 735XT, Forerunner 920XT, Vivoactive, Vivofit 1, 2 and 3, Vivosmart and HR and HR+) Mio (Alpha, Fuse, Flash, Shine), Polar (A300, A360, Active, Loop, M600, V800), Samsung (Gear 2, Gear S, Gear S2, Gear S3), Withings (Pulse, Pulse 02, Pulse Ox), Xiaomi (Mi Brand and Mi Brand 2). |
| <https://doi.org/10.2478/folmed-2018-0012> | Georgiou et al (2018) | Health adults, one study investigated obese adolescents and one study investigated children. | 1680 | Mean age ranged from 9 to 74 | Heart rate variability (HRV) | ECG and Holter monitors. | Lab/controlled | Garmin (Forerunner 935, Premium HRM, Vivoactive HR+, 920XT, 910XT, HRM Tri,), Polar (H10, H7), Wahoo Tickr HR Monitor, Whoop Strap (Strap 2.0), 4IIII (VIIIIVA), 60beat (HR Monitor), BlueLeza (HRM Blue), Cardiosport (TP3), Carre Technologies (Hexoskin), Empatica (E4 Wristband), Cositea (R2 Smart Fitness HRM Wristband), Mad Apparel (Athos), Medronic (HxM Smart HR), Mio (Alpha 2), Qardio (QardioCore), Sony (SmartBand 2), Sunnto (Smart sensor, sport), Wahoo Fitness (Tickr HRM, Tickr X Workout Tracker) |
| 10.2196/30791 | Germini et al (2022) | Not constrained | 4791 | NR | 1)Step count, 2)Heart rate, 3)Energy expenditure | ***Step count***: manual counting either by direct observation or through video. ***Heart rate***: ECG, pulse oximetry, and other activity trackers (4 different devices, NR) ***Energy expenditure:*** Indirect calorimetry | Lab/controlled | Commercial grade primarily included Apple (not specified), Garmin (Vivofit, Forerunner 225, Vivoactive HR, Vivofit 2, Forerunner 235, Vivosmart HR, Forerunner 920XT, ), Polar, (Loop, V800, A300), Fitbit (Flex, Alta, Charge HR, Charge, Ultra, One, Zip, Blaze) , Jawbone (UP24, UP2), Huawei B1, Xiaomi Mi Brand 1 and 2, Withing’s Pulse, Basis Peak and Peak K, |
| <https://doi.org/10.2196/13641> | Giebel & Gissel (2019) | General population | 686 | NR | Atrial fibrillation | ECG/Holter recordings | Lab/controlled | Apple Watch (Series 1), Fitbit (Blaze), Polar (H7) |
| 10.2196/16273 | Haghayegh et al (2019) | Normal sleepers, patients diagnosed with sleep disorders like (periodic limb movement, obstructive sleep apnoea, sleep-disordered breathing, central disorders of hypersomnolence, insomnia,) depression, and Huntington disease. | 438 | Mean ages ranged from 7 to 54 | Sleep | ***Sleep***: PSG | Lab/controlled | Fitbit( Surge, Charge HR, Flex, Alta HR, Charge 2, One, Ultra, Classic, Versa) |
| Unavailable | Henriksen et al (2020) | Largely healthy participants apart from 1 study that examine patients with chronic obstructive pulmonary disease and 1 study that included patients with lower-limb prosthesis. | 456 | Age range from 18 to 90 | 1) Step count and 2) Energy expenditure 3) Physical activity intensity levels: | ***Step count*** (controlled setting): Direct observations and manual counting. ***Step count (free-living)***: accelerometery. ***Energy expenditure (controlled setting)***: Direct and indirect calorimetry. ***Energy expenditure (free-living settings)***: accelerometery. ***Physical Activity intensity levels (free-living conditions):*** Accelerometers. | Lab/controlled and free-living | Polar (V800, A360, A300, M600, M430, Loop) |
| <https://doi.org/10.1007/s00392-021-01941-9> | Hermans et al (2021) | Patients with Atrial Fibrillation | NR | NR | Atrial fibrillation | Not specified but included 24-h Holter monitor, 12-lead ECG or single-lead ECG | Lab/controlled | Fitbit (Charge HR), Apple Watch (Series 3, A1554, Watch 4,) Xiaomi (Amazfit Health Band 1S), Empatica (E4), Wavelet Health, Samsung (Gear Fit 2), Honor (Band 4, Watch), Huawei (Watch GT), Polar (H7) |
| 10.1249/tjx.0000000000000215 | Irwin & Gary (2022) | Adult ambulatory population | 256 | Mean age ranged from 23 to 70 | Heart rate and step count | ECG and polar strap | Lab/controlled | Fitbit Charge 2 |
| 10.1016/j.apmr.2012.11.030 | Kenyon et al (2013) | Adults and children with physical disability | 197 | Mean age ranged from 10 to 67 | Step count | Step count: Manual counting either by direct observation or through video. | Lab/controlled | Yamax Dig-Walker SW (200, 700, 401). |
| 10.1007/s40615-022-01446-9 | Koerber et al (2022) | Adult participants of many skin tones ranging on the Fitzpatrick scale. | 469 | Mean age of 34, range of 20 to 62 | Heart rate | Heart rate: ECG and chest straps (no model provided) | Lab/controlled | Apple, Fitbit, Mio Alpha, and Garmin. (Models were not provided and therefore grouped together based on manufacturer). |
| 10.1080/03091902.2021.2006350 | Leung et al (2021) | Healthy adults of both genders | 927 | Mean age range from 19.8 (1.4) to 49.2 (19.2) | Energy expenditure | Energy expenditure: Indirect calorimetry | Lab/controlled | Fitbit (One, Zip, Flex, Blaze, Charge HR, Classic, Charge 2, Ultra, Alta, Alta HR, Surge) |
| <http://dx.doi.org/10.1093/europace/euaa139> | Lopez Perales et al (2021) | Adults | 15073 | NR | Atrial fibrillation | 12-lead ECG, pacemaker and implantable cardioverter-defibrillator electrograms and cardiac telemetry monitoring | Lab/controlled | Huawei (Band 2, Watch GT), Empatica (E4), Honor (Band 4, Watch), Wavelet Health (Amiigo), |
| [10.1007/s40279-021-01639-y](https://dx.doi.org/10.1007/s40279-021-01639-y) | Molina-Garcia et al (2022) | Physically active/healthy, recreational runners, or soccer players (men and women) | 403 | 24.6 (5.7) | V02 max | Direct or indirect calorimetry (gas analysis) | Lab/controlled | Garmin (Fenix 5X, Fenix 3 + chest HR strap, Forerunner 920XT, GF5) Polar (A300, S410, F11, FT40, F6, RS300X, and two V800), Fitbit (Charge 2) |
| 10.2196/28974 | Nazarian et al (2021) | Individuals with cardiac arrhythmias | 424371 | NR | Cardiac arrhythmias | 12-lead ECG is most cases, a Holter monitor, ECG patch, telemetry or internet-enabled mobile ECG. | Lab/controlled | Apple (NR), Huawei (Watch GT, The Honor Watch, The Honor Band), Huami (Amazfit Health Band IS), Samsung (Simband 2, GearFit, Gear S3), Empatica (E4), Wavelet wristband. |
| <https://doi.org/10.1136/bjsports-2018-099643> | O'Driscoll et al (2018) | Healthy Adults | 1946 | Mean age of 35 years, range 20–86 | Energy expenditure | For criterion validation, we considered DLW, indirect calorimetry systems and metabolic chambers.6 | Both | Apple (Watch, Watch 2), Basis (B1, Peak), Beurer (AS80), Epson (Pulsense), ePulse (Personal Fitness Assistant), Fitbit (Blaze, Charge, Charge 2, Charge HR, Flex, Surge), Garmin (Forerunner 225, Forerunner 920XT, Vivoactive, vivofit, vivosmart, vivosmart HR), Jawbone (UP, UP24), LifeCheck( Calorie sensor), Microsoft (Band), Mio (Alpha), Misfit (Shine), Nike (Fuel Band), Polar (Loop, AW200, AW360), Samsung (Gear S), SenseWear (Armband, Mini, Pro 2, Pro 3), TomTom (Touch), Vivago (Vivago), Withings (Pulse, Pulse 02) |
| 10.2196%2F52192 | Schyvens et al (2024) | Healthy adults; participants with Huntington’s disease | 141 | Mean age: 34 | Sleep | PSG or an ambulatory EEG monitor | Lab/controlled | Fitbit Charge 4, Garmin Vivosmart 4, and WHOOP |
| 10.7759/cureus.35355 | Windisch et al (2023) | Healthy adults in hypoxic conditions, adults and children with congenital heart disease, patients under the age of 23, adults with heart disease, lung disease, and adults with chronic obstructive pulmonary disease and interstitial lung disease. | NR but manually calculated 973 | Mean age ranged from 7.2 to 64 | Blood oxygen saturation (Sp02) | Pulse oximetry | Lab/controlled | Apple Series 6 |
| 10.1080/02640414.2020.1767348 | Zhang et al (2020) | Largely healthy adults | 1738 | Mean age ranged from 8 to 74 | Heart rate | ECG/chest strap | Unclear | Empatica, Fitbit, Apple, Garmin, Mio, TomTom, Basis Peak, Wavelet, PulseOn, Polar, Samsung, Tempo, Philips, Omron, Microsoft, (models not reported) |

NR = Not reported

* Population from participants involved in validation studies only.

**Full narrative synthesis of results of individual reviews.**

A total of 24 studies (8 of which performed some kind of meta-analysis) were included in this umbrella review [1-24].

**Heart rate:**

Of the 24 studies included in this review, six of them investigated the validity of wearables to measure heart rate either at rest or during exercise. In addition, these six systematic reviews contained a total of 5816 participants involved in heart rate validation. The criterion measures included ECG, validated chest straps (namely the Polar chest straps), Holter monitors, telemetry, ICM recordings, and pulse oximetry.

Chevance et al (2022) investigated the validity of a range of Fitbit devices (Charge HR, Charge 2, Blaze, Surge, Versa, Charge 3, Ionic) in both controlled and free-living settings spanning 32 studies, 1022 participants, and 117 comparisons. Fitbit devices underestimated heart rate in comparison to the criterion measures of ECG, pulse oximetry, and Polar chest straps in 56% (18/32) of studies. One study reported an overestimation of heart rate, whereas the remaining 13 studies did not conclude any under or over estimations.

Fuller et al (2020) investigated a large range of wearable devices including smart watches, activity trackers, and devices worn on many locations including the wrist, hip, waist, ankle, shin, and thigh. This review included 29 studies (1023 participants) that investigated heart rate validity in controlled settings with 266 comparisons noted, and three studies (66 participants) in free-living settings with 5 comparisons. For controlled settings, devices were compared to ECG, Polar chest straps, and pulse oximetry. Of the 266 comparisons, 177 measurement error was reported or calculable and of these 177 comparisons 56.5% lay within the acceptable ±3% measurement error threshold for controlled settings, 24.9% fell below the -3% threshold, and 18.6% were above the +3% threshold. The free-living settings which all investigated the Fitbit Charge HR found that all MAPE values fell within the ±10% measurement error threshold for free-living comparisons to Polar chest straps in adults (four studies) and children (one study). Although, all comparisons showed non-statistically significant underestimations that varied depending on activity type.

Germini et al (2022) included 9 studies with in total 309 participants that investigated the validation of wearables for measuring heart rate. The studies compared wearable devices to reference standards such as ECG and pulse oximetry, however they also included comparisons to other activity trackers. Apple watches performed superiorly to other brands with MAPE scores of less than 10% across two studies. The Fitbit devices’ validity varied; the Fitbit Charge showed mean bias ranging from -6 to -9bpm. Whereas the Fitbit Charge HR or Charge HR2 had MAPE scores ranging from 2.4% to 17% across three studies. Lastly the Fitbit Blaze had MAPE scores from 6 to 16% in one study. However, the mixing of criterion measures limits our ability to draw definitive conclusions from this paper and as such is excluded from our synthesis.

Irwin & Gary (2022) solely investigated the validity of the Fitbit Charge 2 for measuring heart rate. This review contained six studies with a total of 161 participants involved in heart rate validation. The Fitbit Charge 2 was more accurate at low-to-moderate intensity levels, with decreases in accuracy at higher intensity levels. Of the studies that reported MAPE (3/6) values were 9.21%, 10.79%, and 69%.

Koerber et al (2022) investigated the impact of darker skin tones on heart rate validity for wearable devices. In total 10 studies with a sample of 469 participants were included in this review. Four studies reported significant reductions in heart rate accuracy, four found no significant differences, and the final two studies had mixed results. No study reported increased accuracy in darker skin tone individuals. It is important to note that only three of the ten studies included participants with Fitzpatrick 6 skin tones (dark skin tone), with the average skin tone in six of the studies being 3.5 (293 participants, white).

Zhang et al (2020) analysed 49 studies with 1738 participants with regards to the validity of heart rate measures of smart watch wearable devices. The criterion measures used as comparisons included ECG and chest straps. Through multilevel random effects meta-analysis, the authors found no statistically significant mean difference between the wrist worn wearables and reference standards during sleep (-0.40 bpm, 95% CI -1.64 to 0.83 bpm), rest (−0.01 bpm 96% CI −0.02 to 0.00 bpm), treadmill activities (walking to running) (−0.51 bpm 95% CI −1.60 to 0.58 bpm), post-exercise (1.30 bpm 95% CI −1.21 to 3.81 bpm), and everyday activities (−1.30 bpm 95% CI −3.76 to 1.16 bpm). Significant differences were found between criterion measures and wearables during resistance training and cycling. Mean differences during resistance training were -7.26 bpm (95% CI -10.46 to -4.07 bpm), and cycling -4.55 bpm (95% CI -7.24 to -1.87 bpm).

**Heart rate variability:**

Board et al (2016) (4/6), investigated the validity of Polar wrist worn devices (V800, RS800CX, and S810) and the Suunto T6 for detecting heart rate variability and the error associated compared to the reference standard of ECG (2, 3, 5, and 12-lead). Twelve studies with a total sample size of 595 total participants investigated several aspects of heart rate variability validity including inter-beat interval detection, aberrant beat detection, inter-beat interval time period, and validity of computed HRV indices in temporal and power spectral domains. The results of this review indicate that some Polar devices namely the Polar S810 and Polar RS800CX are valid for inter-beat interval detection compared to ECG derived data in the seat and standing position. In addition, the Polar RS800CX and the Polar V800 performed well on temporal and power spectral domains namely RMSSD and HF indices. With regards to the inter-beat interval time period validation the Polar RS800CX, Polar V800, and Polar S810 all performed well by demonstrating high intra-class correlation coefficients in both standing and seated positions, whilst also showing low mean bias and narrow limits of agreement. It is however important to note that studies assessing error detection rates had mixed findings. The differing results can be attributed to the different methods for quantifying error rates. As such, error rates in the supine position ranged from 0.082% in the Polar V800 to 6.93% in the Polar S810i. Importantly, the one study that quantified error rates as the number of error beats incorrectly detected by the heart rate monitor saw that the Polar RS800CX failed to detect 85.7% of errant beats that were identified by the ECG recordings. For the standing position, only one study reported error detections rates and this was 0.089% in the Polar V800. Lastly, one study investigating HRV validity during sub-maximal exercise identified error rates of 0.10% for the Polar 810.

The review by Georgiou et al (2018) included 18 studies with a total sample size of 686 participants. The wearables included chest straps, smart watches, smart clothing, and straps worn on the thigh, torso, and legs. Most of the studies bar two investigated healthy adults, one included obese adolescents, and one investigated children. Heart rate variability was investigated in two conditions: rest and exercise. In the rested condition the wearable devices performed well with RR interval correlation ranging from 0.91 to 0.99. Two studies investigated error rate in detection of R waves, and identified error rates of 0.28 and 0.4%. Lastly, wearable devices performed well in comparison to reference standards of Holter monitors in both time and frequency domains, with correlations of 0.98 to 0.99 and 0.85 to 0.94 respectively. With respect to exercise conditions, the validity of these wearable devices decreased as the level of exercise/motion increased. Overall the RR agreement was moderate to excellent with scores ranging from 0.786 to 1. This was also identified in time domain HRV parameters, and a similar pattern occurred in the frequency domain parameters with correlations ranging from 0.8 to 1.

**Cardiac arrhythmia/ Atrial fibrillation:**

Belani et al (2021)

This review included nine studies that investigated the sensitivity and specificity of wearable device brands Apple, Kardiaband, and Samsung. The total number of participants was 1629 individuals, and three studies for each brand were included. The criterion measures included 12-lead ECG, 7-lead Holter monitor, ICM recording, and telemetry, and were conducted in lab controlled settings. Apple devices had average sensitivity values of 97.9% ( 95% CI: 96.1% to 99.7%), and specificity values of 99.61% (95% CI: 98.9% to 100.32%). Kardiaband devices had average sensitivity of 96.9% ( 95% CI = 94.7% to 99.2%), and specificity values of 81.13% (95% CI: 75.19% to 87.08%). Lastly, Samsung devices demonstrated sensitivity values of 95.5% (95% CI = 95.8% to 98.2%), and specificity values of 97.96% (95% CI: 96.71% to 99.22%). Across all devices and brands, the specificity and sensitivity were 97% (95% CI: 95.8% to 98.2%), and 99.02% (95% CI: 98.41% to 99.63%) respectively.

Giebel & Gissel (2019)

This review included 22 studies, however, only two studies investigated commercially available wearable devices validity for detecting atrial fibrillation. The two studies had a combined sample size of 206 hospital patients. The criterion measures used were ECG and Holter monitors. In one study that investigated the Apple Watch Series 1 and the Fitbit Blaze reported high correlation to ECG with regards to detecting atrial fibrillation in patients with sinus rhythm or atrial flutter. The second study investigating the HUAWEI band 2 showed high accuracy compared to 12-lead ECG for detecting atrial fibrillation in hospital patients.

Hermans et al (2021):

Twenty one clinical validation studies investigating the validity of commercial wearable devices for detecting atrial fibrillation were included.Overall sensitivity and specificity for wrist worn wearables and armbands were high and ranged from 67.7% to 100%. Honor Band 4, Huawei Watch GT, and Honor watch scored the highest with 100% sensitivity. Accuracy scores compared to Holter monitors and 12-lead and single lead ECG monitors were 84.9% to 100% and 67.6% to 99% and 60.7 - 100% respectively, highlighting the slightly higher ranges for wearables that were compared to Holter monitors, as opposed to the gold standard of ECG.

Koerber et al (2022)

Apple was the most accurate of the commercial wearable brands. In relation to the effect of darker skin tones on device accuracy, the results were mixed. 4 of the 10 studies reported significant reductions in accuracy, 4 found no significant findings, and 2 studies had mixed results. Important to note, no study reported an increase in accuracy in darker skin tone individuals. Mixed results means we cannot draw definitive conclusions on device accuracy for detecting heart rate and cardiac arrhythmia in people of darker skin tones.

Lopez Perales et al (2021):

This review included 46 studies, however, only 10 of these were relevant to our research question. These 10 studies had a combined 10974 participants ranging from patients to health controls. For detection of atrial fibrillation the sensitivity ranged between 75.4% and 97% and specificity 94% to 100% for smart bands. Smartwatches demonstrated moderate to high sensitivity values of 67.7 to 100% and moderate to high specificity values of 67.6 to 98%.

Nazarian et al (2021):

Eighteen research studies that assessed the validity of cardiac arrhythmia detection in commercially available wearable devices. The most common brands were Apple (seven studies), and Samsung (five studies), with the remaining being spread out across a number of brands like Huawei and Empatica for example. This review had a total sample size of 424371 individuals, and the criterion measure for comparison was most commonly 12-lead ECG, followed by Holter monitors, ECG patches, telemetry, or internet-enabled mobile ECG. All devices were worn on the wrist. The pooled sensitivity for detecting cardiac arrhythmias was 100% (95% CI 0.99 to 1.00) across 17 studies, sensitivity did range from 25% to 100%. A pooled specificity for wearables for detecting cardiac arrhythmias was 95% (95% CI 0.93 to 0.97), this ranged from 68% to 100%. Of the seven studies (1769 participants) that reported on the accuracy of these devices, the pooled accuracy values for detecting cardiac arrhythmias was 97% (95% CI 0.96-0.99).

**Aerobic capacity (V02 max)**

Molina-Garcia et al (2022)

Validation of aerobic capacity (V02 max) was investigated by only one review team. This review included 14 studies with a total of 403 participants. Wearables predict V02 max is numerous ways but it was found that devices that used exercise test to predict V02 max showed very little bias (-0.09 ml.kg-1.min-1, 95% CI -1.66 to 1.48) compared to the gold standard of indirect calorimetry. In addition, it was identified that devices that predicted V02 max whilst the user is at rest significantly over-estimated V02 max with bias of 2.17ml.kg-1.min-1 95% CI 0.28 to 4.07. Importantly to note, both methods of predicting V02 demonstrated large random error with limits of agreement ranging from ±15.24 (95% CI −22.18 to 26.53) for resting predictions and±9.83 (95% CI −16.79 to 16.61) ml·kg−1·min−1 for predictions made using exercise tests.

**Blood 02 saturation:**

Windisch et al (2023)

Only one study included, investigated the validation of blood oxygen saturation measurements (Windisch et al., 2023). This paper examined the Apple Watch Series 6 and compared its measurements to the gold standard pulse oximetry. A total of 973 participants across five studies were included in this review, most were adult patients with history of lung disease, cardiovascular disease, or other cardiac histories. The remaining were children, patients with congenital heart disease and healthy adults. In healthy and patient samples the Apple Watch Series 6 demonstrated moderate to strong Pearson correlation coefficients compared to conventional oximeter readings. Pearson correlation coefficient (LOA) values included 0.81 (-2.7% and 4.1%), 0.89 (-3.5% and 3.0%), 0.76 (-7.0% and 5.0%), 0.813 (NR). The final study included healthy adults and exposed them to hypoxic conditions, these authors identified the Apple Watch Series 6 reported limits of agreement of -5.8% and 5.9%, and that the Apple Watch measured on average 1% higher than the criterion standard.

**Step Count/Wheelchair Push counts**

The criterion measures accepted for step/wheelchair push count were manual counting, either ‘live’ or via video recording.

Byrne et al (2023):

The systematic review by Byrne et al. evaluated the accuracy of wearable physical activity monitors (WPAMs), particularly smartwatches, in measuring wheelchair push counts. The Apple Watch was found to be the most accurate for wheelchair push count detection, with each successive generation showing improved accuracy. Specifically, the Apple Watch Series 4 had the lowest mean absolute percentage error (MAPE) compared to earlier versions. Other devices studied included the Garmin VivoFit, Fitbit Flex, Jawbone UP24, and the Activ8 Professional Activity Monitor. Among these, the Apple Watch consistently outperformed others in accuracy. The findings highlight that the accuracy of WPAMs improves with higher push frequencies and that device calibration enhances performance. However, lower-frequency activities and certain wheelchair manoeuvres, such as turns, posed challenges for accurate detection.

Chevance et al (2022):

Of the 15 studies investigating step count validation of Fitbit devices compared to manual counting, the majority of studies (six) reported underestimations, one reported overestimation, and the remaining six studies did not provide conclusions regarding over-or underestimations. The mean bias upon removal of low quality studies were -3.11 steps per minute.

Evenson et al (2015):

This review included 12 studies that investigated step count validity of commercial wearable devices. Three studies that used manual counting as the criterion measure reported the wearable devices demonstrated high correlation coefficients >=0.80 for the Fitbit Ultra, for treadmill walking and elliptical. However, as the speed of the exercise increased such as running or agility drills correlations dropped. In addition other studies reported that various Fitbit devices (One, Flex, and Ultra) and the Jawbone UP24 to underestimate steps during walking and running on a treadmill. In studies that used accelerometers as the criterion measure, correlations between commercial wearable devices and accelerometers were high (>=0.80) for multiple Fitbit devices (Classic, Ultra, Zip, and One) along with the Jawbone UP. However, studies found that at slower walking speeds several devices like the Fitbit One and Flex, and the Jawbone UP and UP24 underestimated step count during treadmill walking and running.

Feehan et al (2018):

This review included 27 studies (191 accuracy comparisons) that investigated step count validity of Fitbit devices across in controlled settings. Across all comparisons made, 46% (n=88) were within the acceptable measurement error range of ±3%, 51% (n=97) were below -3% measurement error, and 3% (n=6) were above the 3% error. Overall there was a tendency for Fitbit devices to underestimate step count with an estimated mean difference of -9%.

In addition, this review investigated the effect of different speeds of ambulation on the validity of these devices. It was reported that more than 50% of the time for jogging and normal walking speeds the measurement error was within the ±3%. For self-paced, slow, and very slow walking speeds the measurement error was below -3% threshold more than 50% of the time.

Body placement of the device was also reported in this review. For devices worn on the torso or ankle the measurement error was within the ±3% threshold more than 50% of the time. However, for wrist worn devices 70% of the time the measurement error fell below the -3% threshold.

Body motion during activity was also investigated in this review. For normal body motion the measurement error was within the ±3% threshold more than 50% of the time. However, for activities that involved constrained or variable body motion the measurement error fell below the -3% threshold 90% of the time. This ultimately led authors to conclude that Fitbit devices tended to underestimate step counts during these types of activities.

Additionally, this review included 13 studies (20 comparisons) that validated step count in free-living conditions. The criterion measures used here were accelerometers included ActiGraph, activPAL, or Actical and pedometers like Omron or Shimmer. In total 55% of comparisons (n=11) were within the ±10% measurement error range, 30% (n=6) were below the -10% range, and 15% were above the 10% range. Feehan and colleagues do note that measurement error in free-living conditions varied depending on the reference criterion used, body placement of device, and the age and mobility capabilities of participants.

Fuller et al (2020):

This review investigated validation studies in both controlled and free-living settings. Ninety studies employing controlled settings with a total of 979 comparisons to manual counting and accelerometery were included. Of these comparisons 805 had reported or calculable group measurement error. From this analysis 45.2% of comparisons (n=364) were within the ±3% acceptable range, 42.7% (n=344) were below -3%, and 12.1% were above 3%. Indicating an overall tendency to underestimate step count. With regards to free-living studies, 42 were included (84 comparisons) and the criterion standard was accelerometery. Of the 84 comparisons, 69 had reported or calculable group measurement errors, and 42% (n=29) were within the ±10% range, 17% (n=12) were below -10%, and 41% (n=28) were above 10%. Overall tendency to overestimate step count by a mean of 5%. In addition, the remaining 15 comparisons, 11 reporte MAPE scores. Of these MAPE scores 40% were below -10% measurement error and 60% were above 10% error.

Germini et al (2022):

Of the 31 studies included, 29 were relevant to this umbrella review. Several brands and devices were included in this particular review, and as such reporting will be limited to the brands with most research. The Fitbit devices had the largest amount of research performed on them with the majority being targeted at the Fitbit Charge HR and the Fitbit Flex. The reference standards included accelerometry or direct observation. Compared to direct observation the Fitbit Charge HR ( 9 of the 29 studies) had MAPE ranging from -12.7% to 24.1%. The Fitbit Flex which was investigated in 11 of the 29 studies and compared to both manual counting and accelerometry had mean percentage error of -23% to 13%.

The most studied Garmin devices was the Vivofit with five of the 29 studies reporting on this wearable. It demonstrated MAPE ranging between -41% to 18%, all other Garmin devices only had one study. Of the remaining brands and devices, the Polar Loop was the only device included in more than one study. The Polar Loop had MAPE values that ranged from -13% to 27%.

Henriksen et al (2020):

Eleven studies were included in this review that investigated step count validity under both controlled and free-living settings. The Polar Loop was the most investigated in both lab and free-living settings. Overall, agreement varied from poor to good but mixed reporting existed, some stating overestimation and some reporting underestimate of steps. This is likely due to which criterion measure was used (lab based manual counting vs free living accelerometer) which the authors did not distinguish. The other devices which were conducted in free-living settings again saw mixed results. The Polar A300 tended to underestimate steps, the Polar V800 tended to overestimate. The Polar M600 showed moderate agreement to accelerometry and Polar M430 showed strong correlations but high MAPE to accelerometers also.

Irwin & Gary (2022):

This review only included studies that investigated the Fitbit Charge 2, two studies with a total sample size of 35 participants were included that investigated step count validity. The comparisons made between the Fitbit Charge 2 and accelerometry yield MAPE scores of 12.36%, 46%, and 12%, all of which are above the acceptable measurement error of ±10%.

Keyon et al (2013):

Pedometer validity as moderate to excellent in physically disabled adults and children. The validity coefficients varied, but was not clear if this was due to device accuracy or due to variations in physical disabilities. Optimal placement on the trunk was inconsistent. Pedometer step counts compared to manual counting was moderate to excellent but had a wide range of errors.

**Physical Activity**

Evenson et al (2015):

Similar to distance, not many studies were included that investigated physical activity. Only two studies were analysed in this review with a sample of 63 participants. Validation relied on comparisons to accelerometers such as ActiGraph and BodyMedia SenseWear devices, which is the criterion standard for free-living measures of physical activity. Fitbit devices worn on the hip and pocket had mixed results. The Fitbit Zip in a one week long study showed strong correlation to accelerometers during moderate to vigorous activity (Spearman CC 0.86). The second study which involved a two day validation of the Fitbit Zip, One and, Up found all devices tended to overestimate physical activity minutes (89.8, 58.6, 18.0 min/day) respectively, whilst also demonstrating a wide range of intraclass correlation coefficient values of 0.36, 0.46, and, 0.70 respectively.

Feehan et al 2018

Fitbit devices had high MAPE scores of greater than 30%, and measurement errors fall below the acceptable range compared to accelerometers.

Henriksen et al (2020)

This review paper included four studies investigating the validation of physical activity intensity levels. Each study investigated a Polar device (V800, A300, M600, and, M430). These studies were conducted in free-living settings and as such the criterion standard is accelerometers such as ActiGraph and Bodymedia SenseWear. The wearable devices showed mixed results in comparison to criteria standards with Pearson’s correlation coefficients ranging from 0.25 to 0.93 depending on the activity intensity, time spent in specific activities, and all active time, amongst other variables.

Germini et al (2022)

This review included 13 studies that reported time spent in moderate to vigorous physical activity. Eleven of these studies reported this in minutes per day. The reference standard were research grade accelerometers such as Actigraph, and GENEActiv. The Fitbit Flex was the most studied device (three studies) and demonstrated MAPE for time spent in moderate to vigorous activity ranging from 7% to 74%, and mean percentage error ranging from -65% to 10%. Other devices only had one study each and were not included in the main body of Germini’s review.

**Energy expenditure:**

Chevance et al (2022):

In this review the findings were mixed for energy expenditure validation of Fitbit devices. Six studies reported underestimations compared to indirect calorimetry, whilst five studies reported overestimations. One study reported mixed findings that were dependent on the intensity of activity, and the final study did not provide an explicit conclusion about under-or overestimations.

Evenson et al (2015):

This review included eight studies that compared the validity of wearable devices for measuring energy expenditure to the gold standard of indirect (seven studies) and direct (one study) calorimetry. Across these studies it was found that commercial devices under estimated energy expenditure, however, some studies reported over-estimations in energy expenditure compared to indirect calorimetry.

Feehan et al (2018):

Across 88 comparisons the measurement error of Fitbit devices compared to direct and indirect calorimetry fell within the ±3% measurement error only 4% of the time. Fitbit devices underestimated energy expenditure (below -3%) 47% of the time (n=41), and overestimated 49% of the time (n=43).

Fuller et al (2020):

This review included studies employing both controlled and free-settings.Thirty six studies (312 comparisons) involved controlled settings where the criterion measure was direct and indirect calorimetry. Of the 312 comparisons, 305 had reported or calculable group measurement errors. Only 9.2% (n=28) fell within the ±3% range, 54.1% (n=165) fell below -3%, and 36.7% (n=112) were above 3%, indicating a trend to underestimate energy expenditure. With regards to free-living settings, nine studies (22 comparisons) were included. Accelerometry was used as the criterion standard for these free-living settings in addition to one study using doubly labelled water. Seventeen of the 22 comparisons reported or had calculable group measurement error. Only 18% (n=3) were within the ±10% error, 53% (n=9) were below -10%, and 29% (n=5) were above the 10% threshold.

Germini et al (2022):

This review include 24 studies investigating the validity of energy expenditure estimates in wearable devices, however, only 20 were focused on commercially available devices. The Apple watch that consisted of two studies demonstrated MAPE of 15% to 211%. Several Fitbit devices were investigated (Charge, Charge HR, Flex, One, Ultra). The Charge and Charge HR had MAPE for energy expenditure ranging from -4.5% to 75%, -12 to 89% respectively. The Fitbit Flex demonstrated a mean percentage bias of -13% in one study, and the Fitbit One had a mean bias of 2.91 (SD 4.35) kcal/minute in one study also. Finally, the Ultra in one study had Pearson correlation coefficient ranging from 0.24 to 0.67 for different activities. Several Garmin devices were also included, namely the Vivofit, Vivosmart, Vivoactive, and Forerunner 920XT and 225. For the Vivo models the demonstrated MAPE values ranging from -21% to 45%. Whereas the Forerunner 920XT and 225 models demonstrated MAPE values running from -27% to 45% and 31% to 155% respectively. In addition, multiple Polar devices were reported on. For the Polar V800 model, it demonstrated MAPE values between 10% to 40% in one study. The Polar Loop in one study had MAPE values running from 6% to 56%. Lastly the Polar A300 demonstrated Pearson correlation coefficient of 0.74 (P<0.01). Lastly the Withings Pulse had MAPE for EE ranging from -39% to 64% across two studies. Across all brands mentioned the MAPE was greater than 30%.

Henriksen et al (2020):

This review included seven studies that investigated energy expenditure validation in wearable devices across both control and free-living settings. In free-living conditions findings were mixed across Polar devices. The Polar Loop while having a strong correlation to a Bodymedia SenseWear armband mini but had a lower MAPE score in free-living settings. However, the Polar V800 significantly overestimated energy expenditure compared to ActiGraph and was low to moderately accurate. The Polar A300 showed good agreement and the M430 had a strong correlation and excellent agreement to criterion in free-living settings. In lab based settings the Polar Loop and Polar A360 showed poor agreement and overestimated energy expenditure compared to indirect calorimetry. However, the Polar V800 showed strong correlations to indirect calorimetry but significantly underestimated energy expenditure.

Leung et al (2021):

This review included 29 studies all investigating Fitbit devices validity for predicting energy expenditure. The total sample size was 927 individuals. Through the use of a random effects model, the summarised validity coefficient between the Fitbit devices and indirect calorimetry was r = 0.64 (k = 29, 95% CI [0.59, 0.69], P<0.001). The validity of the various Fitbit models were significantly impacted by heart rate measuring capability, activity type, and sedentary activity.

O’Driscoll et al (2018):

This review included 64 devices, and 40 different devices were tested in these included studies. One device was worn on the forearm, six were worn on the upper arm, and 33 were worn on the wrist. Over all activities the Garmin Vivofit, Jawbone UP24, and Sense Wear Armband Pro3 significantly under-estimated energy expenditure relative to the criterion measures. The Apple Watch, Bodymedia CORE armband, Fitbit (Charge HR, Flex), Jawbone UP, Nike FuelBand, SenseWear (Armband, Armband Mini, and Armband Pro2) did not differ significantly from the criteria measures.

For measuring activity energy expenditure the pooled estimates showed a non-significant tendency to underestimate compared to the criterion measure ( ES: -0.34, 95% CI -0.71 to 0.04; n =35, p =0.08) with significant heterogeneity between devices.

During walking and stair climbing, pooled estimates of all devices showed that the wearables did not differ from the criterion measures ((ES: −0.09, 95%CI −0.45 to 0.27; n=55; p=0.62), although significant heterogeneity existed between devices.

For cycling energy expenditure the pooled estimate from devices was significantly lower than criterion measures. There was no significant difference between wearables and criterion measures for measuring energy expenditure during running, sedentary and household tasks. However, the pooled effect showed a trend of significant underestimation of total energy expenditure.

**Distance**

Evenson et al (2015):

Distance was only investigated by one review (Evenson et al., 2015), however, only one study aimed at validating distance on wearables were included in the review by Evenson and colleagues (2015). A study of 30 participants found that at slower speeds hip and pocket worn devices tended to overestimate distance, whereas at faster speeds these devices underestimated distance.

**Sleep**

Three systematic review studies investigating the validity of sleep tracker capabilities of wearable devices met the inclusion criteria of this review. Commercial wearable devices were compared to the criterion measure of polysomnography (PSG).

Evenson et al (2015):

This review as mentioned previously investigated several metrics, including sleep. The authors included five studies, however one involved the validation of research grade devices and as such will be excluded from this synthesis. The total number of participants was 180, and all four studies validated Fitbit devices namely the Fitbit Class, Ultra, and UP (this device was used in two studies). All devices over-estimated total sleep time, whilst under-estimating wake after sleep onset.

Haghayegh et al (2019):

This review included 438 participants across 22 studies. Included participants ranged from normal sleepers as well as patients diagnosed with several sleep disorders. Ten of the included studies investigated old Fitbit models (Flex, Charge HR, One, Ultra, and Classic), all devices overestimated total sleep time compared to PSG (eight studies reported significant and two studies reported non-significant overestimations). All devices underestimated wake after sleep onset time with five reporting significant underestimations and one reporting non-significant underestimations.

Three studies analysed newer sleep staging Fitbit models (Alta HR, Surge, and Charge 2) and reported overestimations of total sleep time (two significant and one non-significant). One study investigated sleep efficiency and reported significant overestimations in comparison to PSG. Three studies reported no significant difference in wake after sleep onset time between devices and PSG. All studies reported underestimations of sleep onset latency (one being significant and two being non-significant).

Feehan et al (2018)

This review included three studies (12 comparisons) that validated Fitbit wrist worn devices for sleep measurement in controlled settings. The free-living portion of this review is excluded from this synthesis as comparisons were made to non-acceptable criterion measures. All three studies found the Fitbit devices overestimated total sleep time, and sleep efficiency by more than 10%. One device when used in the sensitive sleep mode underestimated total sleep time and sleep efficiency by more than 15%. In the one study that investigated sleep-onset latency and time awake after sleep onset found measurement errors ranging from 12 to 180%.

Schyvens et al (2024)

The systematic review by Schyevens et al evaluated the accuracy of the Fitbit Charge 4, Garmin Vivosmart 4, and WHOOP wearables in measuring sleep parameters compared to polysomnography (PSG). The review included eight studies, finding that WHOOP showed the least disagreement with PSG for total sleep time, light sleep, and deep sleep but struggled with REM sleep. The Fitbit Charge 4 demonstrated higher sensitivities for deep and REM sleep stages compared to Garmin Vivosmart 4 and WHOOP. Overall, Fitbit Charge 4 and WHOOP were found to be more appropriate for estimating sleep parameters.

Supplemental Table 3. Table of characteristics of studies included in the 24 systematic reviews and meta-analyses.[25-212][213-414]

| DOI | Device(s) |
| --- | --- |
| 10.1136/heartjnl-2019-316004 | Kardiaband + Apple Watch 6 paired to iPhone 6 |
| 10.1249/01.mss.0000218135.79476.9c | Polar S810 |
| 10.1055/s-2004-817878 | Polar 810s |
| 10.1097/MBP.0000000000000143 | Polar RS800cx |
| 10.1016/j.gaitpost.2017.06.012 | Fitbit One, Fitbit Flex, Fitbit Charge HR, and Jawbone UP24 |
| 10.23736/S0022-4707.20.11151-4 | Fitbit Versa, Polar Ignite, Polar TeamPro Sensor |
| 10.1016/j.gaitpost.2018.04.034 | Fitbit Charge HR and Garmin vivosmart HR |
| 10.1080/1091367X.2017.1331166 | Fitbit Charge HR and Hexoskin |
| 10.2196/25313 | Polar OH1 and Fitbit Charge 3 |
| 10.21037/cdt.2019.06.05 | Apple Watch 3, Fitbit Iconic, Garmin Vivosmart HR, Tom Tom Spark 3 |
| 10.1001/jamacardio.2016.3340 | Fitbit Charge HR, Apple Watch, Mip Alpha. Basis Peak |
| 10.3109/07420528.2015.1054395 | Jawbone UP |
| 10.1016/j.ijcard.2015.03.038 | Fitbit One, Fitbit Flex |
| 10.2174/1874387001408010011 | |
| 10.14288/hfjc.v5i4.144 | Fitbit |
| 10.2340/16501977-1993 | Fitbit One |
| 10.1186/1756-0500-7-952 | Fitbit Zip |
| 10.1053/j.sart.2017.07.006 | Fitbit One, Omron HJ-321. Sportline 340 Strider, Fitbit Force, Nike Fuelband SE, StepWatch Activity Monitor |
| 10.1371/journal.pone.0171720 | Mircosoft Band, Apple Watch, Fitbit Charge HR, and Jawbone UP24 |
| 10.1016/j.jad.2017.04.030 | Fitbit Flex |
| 10.1186/s13104-016-2020-8 | Fitbit One, and Fitbit Flex |
| 10.2196/mhealth.6281 | Fitbit Flex |
| 10.1123/japa.2016-0344 | Misfit Shine and Fitbit Charge HR |
| 10.3138/ptc.2016-40.ep | Fitbit One |
| 10.1136/bjsports-2016-096990 | Fitbit Zip, Fitbit Flex, Jawbone UP24 |
| 10.2522%2Fptj.20140611 | |
| 10.2196%2Fmhealth.7870 | |
| 10.1001/jamainternmed.2016.0152 | JAWBONE UP24, Fitbit Flex, Misfit Shine, EPSON PULSENCE PS-100, Garmin Vivofit, TANITA AM-160, OMRON CaloriScan HJA-403C, and Withings Pulse O2, OMRON Active style Pro HJA-350IT, Panasonic Actimarker EW4800, SUZUKEN Lifecorder EX, and ActiGraph GT3X |
| 10.1016/j.jesf.2014.01.003 | Fitbit Ultra, Lifesource XI-25ant, Omron HJ-324U, Virgin HealthMiles GoZone. |
| 10.1136%2Fbmjsem-2015-000013 | |
| 10.1249/mss.0000000000000778 | Actigraph GT3X+, activPAL, Fitbit One, GENEactiv, Jawbone Up, LUMOback, Nike Fuelband, Omron pedometer, and Z-Machine |
| 2201 - 5655 | Jawbone UP, Nike Fuelband, Fitbit Ultra, NL-2000i, Adidas MiCoach, Body Media FIT Core |
| 10.1016/j.gaitpost.2017.10.010 | Fitbit Ultra |
| 10.24985/ijass.2018.30.1.80 | |
| 10.1016/j.mayocp.2018.09.003 | Fitbit Charge 2 |
| 10.1371/journal.pone.0216891 | Fitbit Charge2, Garmin VivoSmart HR+, Philips Health Watch, Withings Pulse Ox, ActiGraph GT9X-BT, Omron HJ-72OITC |
| 10.1080/1091367X.2019.1577737 | Fitbit Flex and Fitbit Flex 2 |
| 10.15326/jcopdf.3.3.2015.0164 | Fitbit Zip,Fitbit Force, and ActiGraph wGT3X-BT |
| 10.1123/jmpb.2018-0010 | Fitbit Surge 2, Garmin Vivosmart HR+, Leaf Health Tracker, Polar A360, Samsung Gear 2, Spire Activity Tracker. |
| 10.1097/CPT.0000000000000107 | StepWatch Activity Monitor, Fitbit Zip |
| 10.1016/j.gaitpost.2018.01.035 | Fitbit Zip and ActiGraph GT3X+. |
| 10.5507/ag.2016.014 | Garmin Vivofit and Polar Loop |
| 10.1123/pes.2018-0019 | Garmin Vivofit 1, Garmin Vivofit 3 |
| 10.3389%2Ffphys.2016.00391 | |
| 10.2478/hukin-2013-0071 | |
| 10.1016/j.ijpsycho.2013.05.017 | |
| 10.1519/JSC.0000000000001662. | Polar RS800 |
| 10.1089/tmj.2008.0071 | Bioshirt |
| 10.1123/ijspp.2016-0668 | Polar H7 |
| 10.1590%2Fbjpt-rbf.2014.0059 | |
| 10.1016/j.physbeh.2016.03.006 | Fitbit Charge HR |
| 10.23736/s2724-5276.18.05333-1 | Fitbit Charge |
| 10.3233/jhd-170251 | Actiwatch Spectrum Pro, Jawbone UP2 and Fitbit One |
| 10.14302/issn.2379-8572.jog-15-891 | Fitbit Flex |
| 10.21037%2Fatm.2016.07.16 | |
| 10.1007/s12553-019-00392-7 | Fitbit Charge 2 and Sensewear armband mini |
| 10.3138/ptc.59.3.208 | Yamax SW-200 |
| 10.1016/j.hlc.2019.06.221 | Apple (NR), Huawei (Watch GT, The Honor Watch, The Honor Band), Huami (Amazfit Health Band IS), Samsung (Simband 2, GearFit, Gear S3), Empatica (E4), Wavelet wristband. |
| 10.1161/circulationaha.119.044126 | Apple Watch |
| 10.1016/j.cmpb.2020.105753 | Wavelet wristband |
| 10.3390/s20195683 | Samsung Simband 2 |
| 10.1080/02640414.2016.1140220 | Sensewear Armband |
| 10.1055/s-0031-1279768 | Sensewear Armband |
| 10.1055/s-0034-1371837 | Sensewear Armband and Actical |
| 10.1371/journal.pone.0073651 | Sensewear Armband |
| 10.1016/j.jsams.2017.07.022 | Actigraph GT3X+ and BodyMedia Sensewear armband |
| 10.1159/000322109 | Sensewear Armband |
| 10.1249/01.mss.0000126805.32659.43 | SenseWear Pro armband |
| 10.1123/jpah.2014-0136 | Sensewear Armband |
| 10.1080/17461391.2014.949309 | Actiheart and Armband |
| 10.1249/mss.0b013e31825ce76f | Sensewear Armband |
| 10.1093/ajcn/85.3.742 | HealthWear Bodymedia armband |
| 10.1123/jpah.2013-0437 | Sensewear Armband |
| 10.1249/mss.0000000000001388 | Polar M600 |
| 10.12691/jpar-2-2-8 | Apple Watch Series 1, Fitbit Charge, TomTom Touch, and Mio Fuse |
| 10.1109/EMBC.2019.8856375 | Apple Watch Series 3, Samsung Gear S3, Polar M600, and Fitbit Charge 2 |
| 10.1378/chest.1989979 | ActiGraph GT3X+ |
| 10.1097/HCR.0b013e3181d0c191 | NA |
| 10.2478/humo-2013-0019 | S-610 Polar Sport, ActiGraph GT1M |
| 10.1016/s0735-1097(17)33725-7 | |
| 10.1016/j.jsams.2017.04.017 | GENEActiv and ActiGraph GT3X+ |
| 10.2196/jmir.6360 | Movband, Sqord, Zamzee |
| 10.1177/1474515118766037 | Fitbit Charge HR |
| 10.1249/MSS.0000000000001588 | |
| 10.1016/j.biopsych.2017.02.340 | |
| 10.22489/CinC.2017.050-177 | Philips Cardio CM3 Generation-3 |
| 10.1016/j.jelectrocard.2018.10.096 | CardiacSense |
| 10.1016/S0735-1097(20)34190-5 | Apple Watch |
| 10.3390/s20195517 | Everion |
| 10.1109/jtehm.2019.2950397 | Apple Watch 4 |
| 10.1155/2019/4861951 | |
| 10.1001/jama.2018.8102 | |
| 10.1253/circj.71.294 | |
| 10.1109/titb.2010.2047401 | |
| 10.1161/circulationaha.116.024439 | |
| 10.1007/s12325-011-0087-0 | Mircolife BP A200 Plus and OMRON M6 |
| 10.1016/j.amjcard.2013.01.331 | |
| 10.1123/jmpb.2018-0003 | |
| 10.1093/europace/euy176 | |
| 10.1136/heartjnl-2018-313147 | |
| https://www.worldcat.org/title/7793582320 | |
| https://www.worldcat.org/search?q=ti:Preliminary%20evaluation%20of%20a%20wrist%20wearable%20heart%20rate%20sensor%20for%20the%20detection%20of%20undiagnosed%20atrial%20fibrillation%20in%20a%20realworld%20setting&qt=advanced&dblist=638 | |
| https://www.worldcat.org/search?q=ti:Monitoring%20and%20detecting%20atrial%20fibrillation%20using%20wearable%20technology&qt=advanced&dblist=638 | |
| 10.1016/j.hrthm.2018.06.037 | |
| 10.1016/j.ijcard.2013.01.220 | |
| 10.1109/jbhi.2017.2688473 | |
| 10.1161/circulationaha.117.032804 | |
| 10.31686/ijier.vol7.iss1.1293 | Garmin Fenix 5 |
| 10.1123/jmpb.2019-0066 | Garmin Fenix 3 |
| https://www.asep.org/asep/asep/JEPonlineOctober2011Esco.pdf | Polar F11 |
| 10.1080/02640414.2013.879672 | Polar FT40 |
| 10.21037/mhealth.2019.09.07 | Fitbit Charge 2 |
| 10.1249/mss.0000000000002041 | Fitbit Charge 2 |
| https://ijier.net/ijier/article/view/619 | |
| https://ijier.net/ijier/article/view/893 | |
| 10.3390/ijerph16173037 | Polar V800 and Garmin Forerunner 920XT, Garmin vivosmart HR, TomTom Touch, and Withings Pulse Ox |
| 10.1519/jsc.0000000000002931 | Polar V800 and Garmin Forerunner 230 |
| 10.1007/s12662-020-00682-7 | Garmin Fenix 5 |
| 10.1038/s41598-021-98453-3 | Apple Watch Series 6 |
| https://www.apple.com/healthcare/docs/site/Apple_Watch_Arrhythmia_Detection.pdf | |
| 10.1109/EMBC.2019.8856928 | "Simband", which is a multi sensor smart wristwatch developed by Samsung. It consists of different sensors including 8 PPG sensors, a tri axis accelerometer, a single channel ECG lead, a temperature sensor and others |
| 10.1016/j.jacc.2018.03.003 | KardiaBand |
| 10.2196/13850 | KardiaBand |
| 10.1016/j.jacep.2018.10.006 | - |
| https://oce.ovid.com/article/00003017-202002250-00012/HTML | - |
| 10.1001/jamacardio.2018.0136 | AliveCor Kardia |
| 10.1161/CIRCEP.118.006834 | KardiaBand |
| 10.1016/j.jshs.2014.12.007 | Polar S810i |
| 10.1007/s00421-015-3303-9 | Polar's HRMs |
| 10.1111/cpf.12320 | Polar RS800CX |
| 10.1007/s00421-008-0742-6 | Polar S810 |
| 10.1249/MSS.0b013e318184a4b1 | Polar S810 heart rate monitor |
| 10.1111/j.1540-8159.2009.02175.x | Polar S810 |
| 10.1590/S0100-879X2008005000039 | Polar S810i rate meter |
| 10.1007/s00421-011-2079-9 | Polar RS800 |
| 10.1007/s00421-010-1415-9 | Polar S810i, Suunto t6, and an ambulatory ECG system |
| 10.1016/j.ijcard.2019.11.120 | Fitbit (FB) and Apple Watch (AW) |
| 10.1002/pmrj.12424 | Polar H7 and Fitbit Charge 2 |
| 10.1080/02640414.2017.1412235 | Apple Watch 1 and Fitbit Charge HR |
| 10.1371/journal.pone.0251975 | Fitbit Alta, Fitbit Charge 2, Apple Watch 2, Yamax, ActiGraph, and Polar H7 |
| 10.1371/journal.pone.0192691 | Fitbit Charge 2 |
| 10.1249/MSS.0000000000001471 | Apple Watch Series 2, Fitbit Blaze, Fitbit Charge 2, Polar H7, Polar A360, Garmin Vivosmart HR, TomTom Touch, and Bose SoundSport Pulse (BSP) headphones |
| 10.1080/02640414.2018.1491941 | Apple Watch Series 1, Fitbit Surge, Garmin 235, Moto 360, Polar A360, Suunto Spartan Sport, Suunto Spartan Trainer, and TomTom Spark 3 |
| 10.1186/s12877-018-0793-4 | Fitbit Flex and the ChargeHR |
| 10.7326/L16-0353 | Fitbit Surge, Basis Peak, Fitbit Charge, and Mio Fuse |
| 10.2196/16716 | Metamax 3B |
| 10.2196/mhealth.7043 | Apple Watch (model not provided), Fitbit Charge HR, and Garmin Forerunner 225 |
| 10.21037/cdt.2019.04.08 | Fitbit Blaze, Apple Watch, Garmin Forerunner 235, and TomTom Spark Cardio |
| 10.1249/MSS.0000000000001146 | Garmin Vivosmart, Fitbit Charge HR, Polar Loop, Misfit Flash, Jawbone Up Move, and Flyfit, along with 3 smartwatches (Apple Watch Sport, Pebble Smartwatch, and Samsung Gear S) and a smartphone application (Moves) |
| 10.1183/23120541.00006-2019 | The Fitbit Charge HR ™ The Polar ® H7 and Masimo SET ® Rad-5v pulse oximeter were found to provide accurate measurements of heart rate in this population |
| 10.1249/MSS.0000000000001284 | The wrist worn HR monitors assessed were Fitbit Blaze (Fitbit), Apple Watch (Apple), Garmin Forerunner 235 (Garmin), and TomTom Spark Cardio (TomTom) |
| 10.2196/mhealth.8233 | The heart rate monitors used in the study were Fitbit Charge HR and Polar H6 |
| 10.2196/14059 | The names of the 3 consumer grade motion sensors were Garmin Vivosmart 3, Fitbit 1, and Fitbit Charge 2 HR |
| 10.2196/mhealth.7870 | The paper tested the accuracy of 5 wrist worn devices (Apple Watch, Samsung Gear S2, Garmin 735XT, Garmin Vivofit, Fitbit Surge), 2 smartphones (iPhone 6s Plus, Nexus 6P) and the research grade ActiGraph wGT3X BT |
| 10.1080/02701367.2019.1603989 | The text mentions several commercially available activity trackers, including Nike Fuelband, Fitbit devices, and Jawbone devices |
| 10.2196/10828 | - |
| 10.5993/AJHB.43.3.5 | The device names are Apple Watch (1) and Fitbit Charge HR 2 |
| 10.1016/j.pmrj.2017.03.012 | - |
| 10.1080/03091902.2018.1472823 | GV (Garmin Ltd, KS), FBS (Fitbit Inc) |
| 10.2196/10338 | Fitbit Charge 2, and Garmin vivosmart HR+ |
| 10.3390/jpm7020003 | Apple Watch, Basis Peak, Fitbit Surge, Microsoft Band, Mio Alpha 2, PulseOn, and Samsung Gear S2 |
| 10.1111/sms.13488 | GENEActiv (Original) |
| 10.2196/24806 | The device name of the relative criterion standard was ActiGraph GT3X BL |
| 10.1136/bmjsem-2015-000106 | The text mentions 6 wrist worn wearable activity trackers, but does not provide the names of the devices |
| 10.1089/tmj.2017.0263 | The 2 device names are Fitbit Charge HR and Mi Band 2 |
| 10.2196/13084 | Fitbit Charge 2, and Garmin vivosmart HR+ |
| 10.1177/2055207618770322 | The device names were Fitbit Surges, Microsoft Bands, and TomTom Cardios |
| 10.1080/02640414.2018.1560644 | The study assessed the accuracy of 2 wearable devices, the Apple Watch and the Fitbit Charge HR 2, to measure heart rates during treadmill exercise |
| 10.1089/tmj.2017.0264 | Fitbit Surge (FS), Fitbit Charge HR (FC), Microsoft Band 2 (MB), and A&D 101NFC Activity Monitor (A&D) |
| 10.3389/fphys.2017.00725 | Bodymedia Sensewear MF, Polar Loop, Beurer AS80, Fitbit Charge, Fitbit Charge HR, Garmin Vivofit, Garmin Vivosmart, Garmin Vivoactive, Garmin Forerunner 920XT, Withings Pulse O x, and Xiaomi MiBand |
| 10.1371/journal.pone.0154420 | Apple Watch, Fitbit Charge HR, Samsung Gear S, and Mio Alpha |
| 10.2196/mhealth.9754 | Apple Watch 2, Samsung Gear S3, Fitbit Surge, Jawbone Up3, Xiaomi Mi Band 2, and Huawei Talk Band B3 |
| 10.3390/ijerph16203812 | Apple Series 1 Watch, LifeTrak Core C200, and Fitbit Charge HR |
| 10.3109/03091902.2013.831135 | The 6 devices used in the study were 2 Actical and 4 Fitbit devices |
| 10.1249/MSS.0000000000000727 | BMC (BodyMedia Inc., Pittsburgh, PA) |
| 10.1001/jama.2014.17841 | Digi Walker SW-200 pedometer, Zip, 1, Flex, UP24, Fuelband, iPhone 5s, and Galaxy S4 |
| 10.1249/mss.0b013e318299d2eb | The devices used in the study were Actical, Actigraph GT3X, and IDEEA |
| 10.5665/sleep.4990 | Jawbone UP |
| 10.3109/03091902.2015.1050125 | The device name was Fitbit Ultra |
| 10.1186/s12966-015-0201-9 | The names of the devices were Fitbit 1, Fitbit Zip, Jawbone UP, Misfit Shine, Nike Fuelband, Striiv Smart Pedometer, and Withings Pulse |
| 10.3233/978-1-61499-289-9-759 | - |
| 10.1249/mss.0000000000000287 | DL (DirectLife), Fitbit One, Fitbit Zip, Bodymedia FIT armband, Jawbone UP, and Basis B1 Band |
| 10.5665/sleep.4918 | Fitbit Ultra, AMI Motionlogger Sleep Watch, PRMM Actiwatch Spectrum, and IM Systems ActiTrac. However, ActiTrac was not included in the study as it has not been validated for use in pediatrics |
| 10.1007/s11325-011-0585-y | Fitbit |
| 10.1123/jpah.2012-0495 | Fitbit wireless activity tracker |
| 10.1177/1359105313490771 | Fitbit |
| 10.1371/journal.pone.0118723 | Movemonitor, Fibit One, ActivPAL, Nike+ Fuelband, Sensewear Armband Mini, Tractivity, and Up |
| 10.1016/j.jsams.2013.10.241 | Fitbit One |
| 10.1177/2047487316634883 | Fitbit Flex |
| 10.2196/mhealth.6321 | The text mentions Jawbone, Fitbit, Misfit, and Garmin as accelerometer based devices developed for PA monitoring |
| 10.1080/17461391.2016.1255261 | Jawbone UP24 (JU), Basis B1 Band (BB), Withings Pulse (WP), Misfit Shine (MS), Fitbit Flex (FF), Fitbit Zip (FZ), Nike + FuelBand SE (NF), Polar Loop (PL) |
| 10.1177/2055217316634754 | Digi-Walker SW-200 pedometer (Yamax), the UP2 and UP Move (Jawbone), and the Flex and One (Fitbit) |
| 10.1089/tmj.2015.0120 | Omron |
| 10.1136/bmjsem-2017-000254 | The research grade accelerometer mentioned in the text is the GT3X+ produced by ActiGraph. The Fitbit devices are mentioned but not named specifically |
| 10.1519/jsc.0000000000001571 | Jawbone UP 24 |
| 10.1249/mss.0000000000000984 | Jawbone UP |
| 10.1371/journal.pone.0172535 | Fitbit Flex |
| 10.1177/2055102916679012 | The 2 devices used in the study were Actiwatch Spectrum Plus and Fitbit Charge HR |
| 10.1080/03091902.2016.1197978 | The 2 physical activity monitoring devices examined in the study were Fitbit Charge and Jabra Sport Pulse Wireless Earbuds |
| 10.1093/gerona/glw098 | The names of the devices were StepWatch, Fitbit 1, Omron HJ-112, Fitbit Flex, and Jawbone UP |
| 10.2522/ptj.20120525 | The 2 activity monitors examined in this study were the Fitbit Ultra and Nikeϩ |
| 10.2196/jmir.5531 | Jawbone UP |
| 10.1080/03091902.2016.1271046 | The devices mentioned in the text are: Fitbit Charge (FC), Fitbit 1 (FO), and ActiGraph GT3x þ (AG) |
| 10.1016/j.gaitpost.2016.04.025 | The names of the 8 wearable activity monitors assessed in the study were Nike+ FuelBand SE, Jawbone UP 24, Fitbit 1, Fitbit Flex, Fitbit Zip, Garmin Vivofit, Yamax CW-701, and Omron HJ-321 |
| 10.1186/s13102-015-0018-5 | Withings Pulse™ |
| 10.4306/pi.2017.14.2.179 | Actiwatch 2 |
| 10.1177/1460458217720399 | The names of the fitness bands were Garmin Vivofit2, Fitbit Flex, Up3, and Microsoft Band |
| 10.3390/s16050646 | The names of the devices are Basis Health Tracker and Fitbit Flex |
| 10.1249/mss.0000000000000933 | Fitbits One, Zip, and Flex and Jawbone UP24 |
| 10.1371/journal.pone.0154956 | The devices tested in this study were the Withings Smart Activity Monitor Tracker (Pulse O 2 ) ™, NL-2000 pedometer ™, Garmin Vivofit ™, and Fitbit 1 ™ |
| 10.3928/19404921-20150429-03 | Fitbit, ActiGraph GT1M |
| 10.1080/03091902.2016.1253795 | Fitbit 1, Garmin Vivofit, and Jawbone UP |
| 10.1016/j.jsams.2016.10.015 | ActiGraph GT3X+ |
| 10.1080/09593985.2017.1354412 | Fitbit Zip and Garmin Vivofit |
| 10.1371/journal.pone.0161224 | Fitbit Flex |
| 10.1136/bmjopen-2016-011742 | Fitbit Zip |
| 10.1093/ptj/pzx010 | Garmin Vivofit and Fitbit Charge |
| 10.2196/cancer.6935 | ActiGraph GT3+, Fitbit 1 |
| 10.1016/j.ijcard.2018.02.073 | Apple Watch Series 1 and Fitbit Blaze |
| 10.1089/g4h.2018.0087 | - |
| 10.1249/MSS.0000000000001146 | Garmin Vivosmart, Fitbit Charge HR, Polar Loop, Apple Watch Sport, Pebble Smartwatch, Samsung Gear S, Misfit Flash, Jawbone, Flyfit |
| 10.1177/0890117118763273 | Garmin Vivofit2, Jawbone UP2, Fitbit One, Apple Watch, Fitbit Charge |
| 10.4070/kcj.2018.0323 | Apple Watch Series 2, Samsung Galaxy Gear S3, and Fitbit Charge 2 |
| 10.1123/japa.2018-0036 | The study used 7 different models of physical activity monitors: Fitbit Charge, Garmin Vivofit, Fitbit Zip, Yamax SW-200 Digiwalker Pedometer, Accusplit AX2710 Accelerometer Pedometer, Yamax EX-510 Pedometer, and Omron HJ-321 Pedometer |
| 10.1080/09638288.2019.1587792 | Polar Loop, Fitbit Flex, MOVEBAND, Garmin Vivofit, and a Fitbit Charge |
| 10.1249/MSS.0000000000001569 | Fitbit Zip, Yamax Digiwalker, Actigraph GT9X, New Lifestyle NL-2000, Fitbit Charge, ActivPAL, Step Watch |
| 10.2196/10972 | The devices tested were Fitbit Charge, Polar M600, Huawei Watch, and Asus Zenwatch 3 |
| 10.2196/cardio.8199 | The devices used in the study were: wGT3X+ sensor (Actigraph, Pensacola, Florida, USA); Model X6-2mini ("X6") accelerometer (Gulf Coast Data Concepts, LLC, Waveland, Mississippi, USA); FBT (Fitbit Inc., San Francisco, California, USA) and GAR (Garmin Ltd., Schaffhausen, Switzerland), which were worn on 2 separate days within 1 week |
| 10.1111/dmcn.14207 | The hip based tracker is called Fitbit 1 and the wrist based tracker is called Fitbit Flex |
| 10.1155/2018/3521271 | Misfit Shine, Axivity AX3, Fitbit Flex |
| 10.2196/mhealth.8524 | The names of the 9 selected activity trackers were Accupedo, Activ8, Digi Walker CW-700, Fitbit Flex, Lumoback, Moves, Fitbit 1, UP24, and Walking Style X |
| 10.1080/03091902.2018.1472822 | The 2 devices mentioned in the text are Fitbit Zip and Fitbit Flex |
| 10.1371/journal.pone.0169616 | The study assessed 5 activity monitors: the Jawbone UP™, Withings Smart Activity Monitor Tracker (Pulse O 2 )™, NL-2000 pedometer™, ActivPAL micro™ and Fitbit 1™ |
| 10.3390/s17010211 | The devices used in the study were Garmin Vivofit 2, Fitbit Charge HR, and 3 other activity trackers |
| 10.1123/apaq.2016-0055 | Fitbit Zip |
| 10.1080/02640414.2018.1535563 | The 2 devices used in the study were the Garmin Forerunner 235 and the Polar RS400 |
| 10.1249/MSS.0000000000001090 | Withings Pulse |
| 10.2196/jmir.5916 | The names of the devices used in the study were iHealth activity monitor (IH), Withings Pulse O2 (WI), Misfit Shine (MF), and Garmin vívofit (GA) |
| 10.1111/sms.13074 | The device name was ActiGraph wGTX+ |
| 10.1371/journal.pone.0157229 | The device names are Polar A300™ and SenseWear™ |
| 10.21037/atm.2016.07.16 | Polar V800 |
| 10.1016/j.ijmedinf.2017.03.008 | The device used for in home, unattended polysomnography was Dream ® by Medatec, Belgium |
| 10.2196/11437 | The devices used in the study were HUAWEI Mate 9 and HUAWEI Honor 7X mobile phones, HUAWEI Band 2 smart bands, and a 12-lead ECG |
| 10.1519/jsc.0000000000001519 | The pulse finger sensor device used in the study was ithlete |
| 10.1055/s-2007-964995 | The 2 devices used were the Polar S810 HRM and the Physiotrace Estaris ECG recorder |
| 10.1007/s13246-014-0271-z | The device used in the study is called the GOW system |
| 10.1088/1361-6579/aa9047 | The device used in the study was Embletta MPR with ST+ Proxy. The participants also wore 2 commercially available personal trackers called Fitbit Surge |
| 10.1016/j.sleep.2019.04.006 | Beddit 3 Sleep Monitor © |
| 10.1111/jsr.12789 | Fitbit Alta HR |
| 10.1080/07420528.2017.1413578 | Fitbit Charge 2™ |
| 10.1016/j.jpsychores.2017.03.009 | Embletta X100 |
| 10.1080/15402002.2019.1651316 | The name of the device is AW Spectrum Classic |
| 10.3390/ijerph15061265 | Jawbone UP3 |
| 10.1007/s41666-018-0013-1 | The wearable devices mentioned in the text are Neuroon and Sleep Shepherd |
| 10.1089/tmj.2018.0202 | The device used in the study was Fitbit Alta |
| 10.1080/07420528.2018.1466800 | The device name was FitBit HR Charge |
| 10.1016/j.jpsychores.2019.109822 | SleepScope |
| 10.1186/s13102-017-0089-6 | Suunto Ambit 2, Garmin Forerunner 920XT, Polar V800﻿ |
| 10.1109/ACCESS.2017.2721098 | The text does not answer the question |
| 10.2196/14438 | Polar M430 |
| 10.1249%2FMSS.0000000000002041 | The text does not answer the question |
| 10.1371/journal.pone.0222569 | The names of the 6 activity monitors evaluated were Withings Go (WGO), Fitbit Charge 2 (FC2), Garmin vı ´vofit (GV1), Garmin vı ´vofit 3 (GV3), Omron HJ-322U E (OMR), and SmartLAB walk+ (SLW) |
| 10.1177/0269215508098895 | the Yamax digiwalker (Model SW-200, Yamax Corporation, Tokyo, Japan) |
| 10.1093/ageing/afn097 | Yamax Digi Walker SW-200 |
| 10.1682/JRRD.2007.05.0069 | Össur patient activity monitor (PAM) |
| 10.1123/apaq.24.3.228 | The name of the pedometer used in the study was Walk4Life (WL) |
| 10.1191/1352458505ms1161oa | The device name was Yamax SW-200 and SW-401 pedometers |
| 10.1097/00005768-200203000-00002 | The devices used were SAM (Stridalyzer Activity Monitor) and a pedometer |
| 10.1038/s41746-020-0226-6 | The wearable devices tested were Apple Watch 4, Fitbit Charge 2, Garmin Vivosmart 3, Xiaomi Miband 3, Empatica E4, and Biovotion Everion |
| 10.1055/a-0875-4033 | Polar OH1 |
| 10.1016/j.jelectrocard.2020.04.017 | CardiacSense |
| 10.1109/PerComWorkshops51409.2021.9431120 | Moto 360, LG Urbane, Polar 600M, Huawei Watch 2, Ticewatch Pro, Fossil Carlyle HR, Misfit Vapor X |
| 10.1007/s10916-019-1325-2 | Apple Watch |
| 10.3109/03091902.2015.1047536 | Omron HR500U and Mio Alpha |
| 10.1088/1361-6579/aa5dd7 | Empatica E4 |
| 10.1016/j.jacc.2018.03.003 | Kardia Band (KB) + Apple watch |
| 10.1056/nejmoa1901183 | Apple Watch |
| 10.2196/14909 | Huawei WatchGT, Honor Watch, and the Honor Band4 |
| 10.1038/s41598-019-49092-2 | Samsung Simband |
| 10.1093/europace/euz060 | The ECG device used was Faros 360. The PPG device used was Empatica E4 wrist band |
| 10.1016/j.jacc.2019.08.019 | Honor Band 4, Huawei Watch GT, Honor Watch |
| 10.1016/j.hrthm.2020.01.034 | Amazfit Health Band 1S |
| 10.1161/circep.120.009260 | Apple Watch Series 4 and 5 |
| 10.1080/03091902.2016.1193238 | Garmin vivofit |
| 10.1007/s00421-011-2269-5 | The device name was SenseWear armband (SWA) |
| 10.1136/bjsm.2008.048868 | SenseWear Pro 2 Armband (BodyMedia) |
| 10.1111/j.1600-0412.2011.01172.x | SenseWear Pro 2 Armband |
| 10.1016/j.jsams.2014.11.001 | Actical |
| 10.1136/bjsm.2007.045575 | Polar AW200 |
| 10.1186/s12966-014-0119-7 | Actiheart Monitor (AH) |
| 10.1038/ejcn.2014.241 | SenseWear Mini Armband |
| 10.1249%2FMSS.0b013e3181fc7162 | The text does not answer the question |
| 10.1139/apnm-2015-0461 | Device name: Actical Physical Activity Monitor |
| 10.1249/01.mss.0000128144.91337.38 | SenseWear™ Armband |
| 10.1016/j.apmr.2009.10.024 | Yamax Digiwalker SW701, SenseWear armband |
| 10.1249/mss.0b013e3181e0b3ff | SenseWear Pro3 and SenseWear Mini |
| 10.1249/mss.0000000000000691 | ActiGraph, activPAL, Core Armband |
| 10.1249/01.mss.0000132379.09364.f8 | The physical activity monitors used for this study were CSA (Actigraph), TriTrac R3D, RT3, BioTrainer Pro, and SenseWear Armband |
| 10.1249/mss.0b013e31820750f5 | SenseWear Pro3 Armband |
| 10.1080/02640414.2011.609899 | ePulse Personal Fitness Assistant |
| 10.1111/sms.12920 | SenseWear Armband Mini |
| 10.1093/gerona/glr101 | SenseWear Pro armband |
| 10.1249/mss.0000000000000529 | The device mentioned in the text is SenseWear Mini |
| 10.1016/j.ypmed.2012.02.013 | Actigraph GT1M |
| 10.1109/iembs.2009.5333124 | LifeChek® calorie sensor |
| 10.1038/oby.2006.260 | The device mentioned in the text is called SenseWear Pro 2 Armband |
| 10.1016/j.jsams.2013.08.007 | The 2 devices mentioned in the text are the SenseWear Armband Mini (SWAM) and the BodyMedia FIT (FIT) |
| 10.1002/oby.20363 | The device used in the study was the SenseWear (SWA) Pro 2 armband |
| 10.1519/jsc.0b013e3182a1f836 | SWA (BodyMedia, Inc., Pittsburgh, PA, USA) |
| 10.1080/02640414.2014.981846 | SenseWear Armband |
| 10.1080/02640414.2017.1397282 | Apple Watch™ |
| 10.1145/3329189.3329215 | Empatica E4, Everion, Fitbit Charge HR, Polar OH1, Wahoo Ticker Fit |
| 10.1080/03091902.2017.1333166 | Garmin Forerunner 225 |
| 10.2196/11040 | Wavelet Health Wristband |
| 10.2196/11889 | Apple Watch Sport 42 mm (1st generation) |
| 10.1080/07420528.2019.1596947 | Fitbit Charge 2™ and SOMNOtouch NIBP™ |
| 10.1088/1361-6579/aac9a9 | PulseOn optical heart rate (OHR) monitor prototype developed by PulseOn Ltd, Espoo, Finland |
| 10.2196/mhealth.6893 | Philips Health Watch |
| 10.1055/s-0043-120195 | Apple Watch Sport devices (Series 0) |
| 10.2196/jmir.6025 | Fitbit Charge HR |
| 10.1111/psyp.13441 | E4 (Empatica) |
| 10.2196/14120 | Tempo HR, and the Polar A370 |
| 10.3138/ptc.2018-25 | Fitbit Charge |
| 10.3390/children5030038 | Fitbit Charge HR |
| 10.1007/978-981-10-5122-7_37 | PulseOn, Empatica E4 |
| 10.1186/s13102-018-0098-0 | Philips Health |
| 10.1016/j.gaitpost.2016.10.014 | ActiGraph GT3X+ |
| 10.1080/09638288.2017.1288764 | ActivPAL TM |
| 10.1152/japplphysiol.00374.2009 | The device used to measure physical activity was the Actigraph GT1M accelerometer |
| 10.1177/0269215518788116 | The devices used in the study were Actigraph W, Actigraph H, Actigraph a, and Metamax 3B |
| 10.1682/jrrd.2006.06.0058 | Actiwatch Score (AW S) |
| 10.1186/s12889-016-3059-0 | Withings Pulse, Misfit Shine, Jawbone Up24, and Fitbit Flex |
| 10.1123/jsr.2013-0140 | The device used in the study was ActiGraph GT3X+ |
| 10.1186/s12884-018-1941-8 | The watch used in the study was referred to as Optical Heart Rate Monitor (OHRM) |
| 10.1371/journal.pone.0190753 | ADAMO Care Watch |
| 10.1177/2047487317738593 | The 2 devices used in the study were Armband Sensewear and ONStep 400 pedometer |
| 10.1186/s12874-019-0668-1 | ActiGraph GT3X+ |
| 10.1002/acr2.11099 | Fitbit Flex and ActiGraph GT3X+ |
| 10.1249/MSS.0b013e31829ba765 | GENEActiv (ActivInsights Ltd, Cambridgeshire, UK) and ActiGraph GT3X+ (ActiGraph LLC, Pensacola, FL) |
| 10.1249/MSS.0b013e31825e19fd | GENEA |
| 10.1002/gps.5064 | GENEactiv Original |
| 10.1089/tmj.2015.0026 | The device name was AX3 data logger |
| 10.1089/tmj.2014.0105 | Fitbit Zip and Nike FuelBand |
| 10.1093/jamia/ocy067 | Fitbit Charge 2 |
| 10.1161/jaha.118.009351 | CM3 Generation-3 |
| 10.1088/1361-6579/aa8830 | HeartSensor HRS-07UE earlobe PPG sensor |
| 10.2196/18050 | 1st generation Apple Watch (model A1554) |
| 10.2196/14857 | Apple Watch Series 3, Fitbit Charge HR |
| 10.2196/16443 | The 2 devices used in the study were CART (Sky Labs Inc, Seongnam, Republic of Korea) and a conventional medical grade pulse oximeter (iDAQ-400 with PPG AMP and P400, PhysioLab Inc, Busan, Republic of Korea) |
| 10.1109/embc.2016.7591456 | Samsung Simband |
| 10.1038/s41598-019-48267-1 | The device used in the study was a wearable ECG that is a wireless single lead device with an electrode embedded into a T shirt for community screening to identify AF |
| 10.1186/s12933-020-01128-y | The device used for ECG screening was the iRhythm Zio XT |
| 10.1016/j.amjcard.2018.07.003 | Polar H7 (PH7), Firstbeat Bodyguard 2 (BG2), AliveCor, and WatchBP |
| 10.1111/pace.13800 | RhythmPad |
| 10.1016/j.jelectrocard.2014.08.012 | ECG patches |
| 10.1002/clc.22387 | The device used for monitoring was the Zio wearable patch based device by iRhythm Technologies, Inc |
| 10.1016/j.hrthm.2018.08.012 | The device name was Zio Patch |
| 10.1177/1741826711406060 | The device used for recording and telephone transmission of 12-lead ECG was called CardioVox P12 heart line receiving system by Aerotel, Holon, Israel |
| 10.1016/j.jdiacomp.2020.107711 | The device used for non invasive home monitoring was an external event triggered ECG loop recorder device (ELR) called R.Test Evolution 4® made by NorDiaTech, Paris, France |
| 10.1007/s00380-016-0866-2 | - |
| 10.1016/j.ijcard.2003.12.005 | The device name was Card Guard 7100 |
| 10.1089/tmj.2011.0121 | either a 12-lead electrocardiogram (ECG) recording device (CG-7100, Card Guard Scientific Survival Ltd., Rishon Le Zion, Israel) or a single lead device (CG-2100, Card Guard Scientific Survival Ltd.) |
| 10.1007/s10072-015-2231-0 | BP3MQ1-2D: AFib |
| 10.1136/bmjopen-2013-004565 | The device name was Omron monitor (model HCG-801) |
| 10.1136/bmjopen-2015-010745 | The name of the device used for BP measurement and identification of pulse irregularities compatible with AF was Microlife WatchBP Office AFIB |
| 10.1038/jhh.2009.5 | Microlife BPA100 Plus |
| 10.1038/ajh.2009.98 | The device used in the study was an oscillometric automatic blood pressure monitor with an irregular heartbeat detection feature, model BP3MQ1-2D, manufactured by Microlife USA |
| 10.1111/j.1540-8159.2004.00499.x | The device name was Omron 712C automatic sphygmomanometer |
| 10.1186/s13104-015-1837-x | - |
| 10.1080/02640414.2018.1481723 | Basis watch, Fitbit Flex, Polar FT7, Jawbone, Omron, and Actigraph |
| 10.1136/bjsports-2013-093154 | accelerometer |
| 10.1249/01.mss.0000538181.87422.7a | Fitbit Charge HR2, Apple Watch |
| 10.1161/jaha.118.008585 | The smartphone application used in the study was Cardiio Rhythm |
| 10.1016/j.hrthm.2012.12.001 | The device name was iPhone 4S |
| 10.1109/embc.2012.6346146 | iPhone 4s |
| 10.1016/j.amjcard.2018.01.035 | Cardiio Rhythm Mobile Application (CRMA) |
| 10.1111/jce.12842 | smart phone app |
| 10.1109/EMBC.2017.8036773 | The device names were iPhone 5s, iPhone 6 Plus and iPhone 6s Plus |
| 10.2196/12284 | The ECGs were taken using digital machines CardiMax FCP-7101 (Fukuda Denshi), CP 50 (Welch Allyn), Universal ECG (QRS Diagnostic), and ECG-1150 (Nihon Kohden Corporation) |
| 10.1111/jce.12634 | AliveCor |
| 10.1093/europace/euw025 | The 2 handheld ECG devices used in the study were MyDiagnostick and AliveCor |
| 10.1186/1471-2296-15-7 | - |
| 10.1249/01.mss.0000135794.01507.48 | The device used in the study was Polar S410 Heart Rate Monitor |
| 10.1177/20552076221132127 | Apple Watch Series 6 |
| 10.1007/s00246-022-02987-w | Apple Watch Series 6 |
| 10.1371/journal.pdig.0000051 | Apple Watch Series 6 |
| 10.3390/jcm11061467 | Apple Watch 6 |
| 10.1136/jnnp-2021-EHDN.78 | Fitbit Charge 4 |
| 10.1136/jnnp-2022-ehdn.154 | Fitbit Charge 4 |
| 10.1371/journal.pone.0275287 | Fitbit Charge 4 |
| 10.1080/02640414.2020.1797448 | Whoop 2.0 |
| 10.2147/NSS.S270705 | Apple Watch Series 3 (Apple, Cupertino, California, United States), Beddit Sleep Monitor 3.0 (previously Beddit, Espoo, Finland, manufacturing now owned by Apple), Fatigue Science Readiband (Fatigue Science, Vancouver, British Columbia), Fitbit Ionic (Fitbit, San Francisco, California, United States), Garmin Vivosmart 4 (Garmin, Olathe, Kansas, United States), 2nd generation Oura smart ring (ŌURA, Oulu, Finland), Polar A370 (Polar, Kempele, Finland), and the WHOOP Strap 2.0 (WHOOP, Boston, Massachusetts, United States). |
| 10.3390/s22166317 | Apple Watch S6, Garmin Forerunner 245, Polar Vantage V, Oura Ring Generation 2, WHOOP 3.0 and Somfit |
| 10.3390/bios11060185 | Whoop 2.0 |
| 10.1371/journal.pone.0243214 | Garmin Vivosmart 4 |
| 10.1055/s-0041-1740236 | Apple Watch Series 4, Apple Watch Series 1 |
| 10.3205/mibe000208 | Apple Watch Series 4, Fitbit Flex 2 |
| 10.1016/j.rehab.2020.03.007 | Fitbit Charge 2, Garmin vivosmart HR+ |
| 10.1016/j.apmr.2017.09.095 | Apple Watch (model not provided) |
| 10.1080/10790268.2019.1576444 | Apple Watch (series 1) |
| 10.1371/journal.pone.0191556 | Garmin Vivofit, Fitbit Flex, and Jawbone UP24 |
| 10.1371/journal.pone.0194864 | Activ8 Professional Activity Monitors |

**REFERENCES**

1. Belani S, Wahood W, Hardigan P, Placzek AN, Ely S. Accuracy of Detecting Atrial Fibrillation: A Systematic Review and Meta-Analysis of Wrist-Worn Wearable Technology. Cureus. 2021 Dec;13(12):e20362.

2. Board EM, Ispoglou T, Ingle L. Validity of Telemetric-Derived Measures of Heart Rate Variability: A Systematic Review. Journal of Exercise Physiology Online. 2016;19(6):64-84.

3. Chevance G, Golaszewski NM, Tipton E, Hekler EB, Buman M, Welk GJ, et al. Accuracy and Precision of Energy Expenditure, Heart Rate, and Steps Measured by Combined-Sensing Fitbits Against Reference Measures: Systematic Review and Meta-analysis. JMIR mHealth and uHealth. 2022;10(4):e35626.

4. Evenson KR, Goto MM, Furberg RD. Systematic review of the validity and reliability of consumer-wearable activity trackers. International Journal of Behavioral Nutrition & Physical Activity. 2015;12:1-22.

5. Feehan LM, Geldman J, Sayre EC, Park C, Ezzat AM, Yoo JY, et al. Accuracy of Fitbit Devices: Systematic Review and Narrative Syntheses of Quantitative Data. JMIR Mhealth Uhealth. 2018 Aug 9;6(8):e10527.

6. Fuller D, Colwell E, Low J, Orychock K, Tobin MA, Simango B, et al. Reliability and Validity of Commercially Available Wearable Devices for Measuring Steps, Energy Expenditure, and Heart Rate: Systematic Review. JMIR mHealth and uHealth. 2020;8(9):e18694.

7. Georgiou K, Larentzakis AV, Khamis NN, Alsuhaibani GI, Alaska YA, Giallafos EJ. Can Wearable Devices Accurately Measure Heart Rate Variability? A Systematic Review. Folia medica. 2018;60(1):7-20.

8. Germini F, Noronha N, Debono VB, Philip BA, Pete D, Navarro T, et al. Accuracy and Acceptability of Wrist-Wearable Activity-Tracking Devices: Systematic Review of the Literature. Journal of Medical Internet Research. 2022;24(1).

9. Giebel GD, Gissel C. Accuracy of mHealth Devices for Atrial Fibrillation Screening: Systematic Review. JMIR Mhealth Uhealth. 2019 Jun 16;7(6):e13641.

10. Haghayegh S, Khoshnevis S, Smolensky MH, Diller KR, Castriotta RJ. Accuracy of Wristband Fitbit Models in Assessing Sleep: Systematic Review and Meta-Analysis. Journal of Medical Internet Research. 2019;21(11):N.PAG-N.PAG.

11. Henriksen A, Johansson J, Hartvigsen G, Grimsgaard S, Hopstock L. Measuring Physical Activity Using Triaxial Wrist Worn Polar Activity Trackers: A Systematic Review. Int J Exerc Sci. 2020;13(4):438-54.

12. Hermans ANL, Gawalko M, Dohmen L, van der Velden RMJ, Betz K, Duncker D, et al. Mobile health solutions for atrial fibrillation detection and management: a systematic review. Clinical Research in Cardiology. 2022;111(5):479-91.

13. Irwin C, Gary R. Systematic Review of Fitbit Charge 2 Validation Studies for Exercise Tracking. Transl J Am Coll Sports Med. 2022 Fall;7(4):1-7.

14. Kenyon A, McEvoy M, Sprod J, Maher C. Validity of Pedometers in People With Physical Disabilities: A Systematic Review. Archives of Physical Medicine & Rehabilitation. 2013;94(6):1161-70.

15. Koerber D, Khan S, Shamsheri T, Kirubarajan A, Mehta S. Accuracy of Heart Rate Measurement with Wrist-Worn Wearable Devices in Various Skin Tones: a Systematic Review. Journal of racial and ethnic health disparities. 2022.

16. Leung W, Case L, Sung MC, Jung J. A meta-analysis of Fitbit devices: same company, different models, different validity evidence. Journal of Medical Engineering and Technology. 2022;46(2):102-15.

17. Lopez Perales CR, Van Spall HGC, Maeda S, Jimenez A, Laţcu DG, Milman A, et al. Mobile health applications for the detection of atrial fibrillation: A systematic review. Europace. 2021;23(1):11-28.

18. Molina-Garcia P, Notbohm HL, Schumann M, Argent R, Hetherington-Rauth M, Stang J, et al. Validity of Estimating the Maximal Oxygen Consumption by Consumer Wearables: A Systematic Review with Meta-analysis and Expert Statement of the INTERLIVE Network. Sports Medicine. 2022;52(7):1577-97.

19. Nazarian S, Lam K, Darzi A, Ashrafian H. Diagnostic Accuracy of Smartwatches for the Detection of Cardiac Arrhythmia: Systematic Review and Meta-analysis. Journal of Medical Internet Research. 2021;23(8):N.PAG-N.PAG.

20. O'Driscoll R, Turicchi J, Beaulieu K, Scott S, Matu J, Deighton K, et al. How well do activity monitors estimate energy expenditure? A systematic review and meta-analysis of the validity of current technologies. British journal of sports medicine. 2020;54(6):332-40.

21. Windisch P, Schröder C, Förster R, Cihoric N, Zwahlen DR. Accuracy of the Apple Watch Oxygen Saturation Measurement in Adults: A Systematic Review. Cureus. 2023 Feb;15(2):e35355.

22. Zhang Y, Weaver RG, Armstrong B, Burkart S, Zhang S, Beets MW. Validity of Wrist-Worn photoplethysmography devices to measure heart rate: A systematic review and meta-analysis. Journal of Sports Sciences. 2020;38(17):2021-34.

23. Byrne J, Lynch S, Shipp A, Tran B, Mohan S, Reindel K. Investigating the Accuracy of Wheelchair Push Counts Measured by Fitness Watches: A Systematic Review. Cureus. 2023 Sep;15(9):e45322.

24. Schyvens AM, Van Oost NC, Aerts JM, Masci F, Peters B, Neven A, et al. Accuracy of Fitbit Charge 4, Garmin Vivosmart 4, and WHOOP Versus Polysomnography: Systematic Review. JMIR Mhealth Uhealth. 2024 Mar 27;12:e52192.

25. Abt G, Bray J, Benson AC. The validity and inter-device variability of the Apple Watch for measuring maximal heart rate. J Sports Sci. 2018 Jul;36(13):1447-52.

26. Adam Noah J, Spierer DK, Gu J, Bronner S. Comparison of steps and energy expenditure assessment in adults of Fitbit Tracker and Ultra to the Actical and indirect calorimetry. J Med Eng Technol. 2013 Oct;37(7):456-62.

27. Al-Kaisey AM, Koshy AN, Ha FJ, Spencer R, Toner L, Sajeev JK, et al. Accuracy of wrist-worn heart rate monitors for rate control assessment in atrial fibrillation. Int J Cardiol. 2020 Feb 1;300:161-4.

28. Alharbi M, Bauman A, Neubeck L, Gallagher R. Validation of Fitbit-Flex as a measure of free-living physical activity in a community-based phase III cardiac rehabilitation population. Eur J Prev Cardiol. 2016 Sep;23(14):1476-85.

29. Aliarzadeh B, Greiver M, Moineddin R, Meaney C, White D, Moazzam A, et al. Association between socio-economic status and hemoglobin A1c levels in a Canadian primary care adult population without diabetes. BMC Fam Pract. 2014 Jan 10;15:7.

30. Alinia P, Cain C, Fallahzadeh R, Shahrokni A, Cook D, Ghasemzadeh H. How Accurate Is Your Activity Tracker? A Comparative Study of Step Counts in Low-Intensity Physical Activities. JMIR Mhealth Uhealth. 2017 Aug 11;5(8):e106.

31. Alsubheen SA, George AM, Baker A, Rohr LE, Basset FA. Accuracy of the vivofit activity tracker. J Med Eng Technol. 2016 Aug;40(6):298-306.

32. An HS, Jones GC, Kang SK, Welk GJ, Lee JM. How valid are wearable physical activity trackers for measuring steps? Eur J Sport Sci. 2017 Apr;17(3):360-8.

33. An HS, Kim Y, Lee JM. Accuracy of inclinometer functions of the activPAL and ActiGraph GT3X+: A focus on physical activity. Gait Posture. 2017 Jan;51:174-80.

34. Anderson JC, Chisenall T, Tolbert B, Ruffner J, Whitehead PN, Conners RT. Validating the Commercially Available Garmin Fenix 5x Wrist-Worn Optical Sensor for Aerobic Capacity. International Journal for Innovation Education and Research. 2019 01/01;7(1):147-58.

35. Apple. Using Apple Watch for Arrhythmia Detection; 2020.

36. Ashok K, Bhargava CN, Asokan R, Pradeep C, Pradhan SK, Kennedy JS, et al. CRISPR/Cas9 mediated editing of pheromone biosynthesis activating neuropeptide (PBAN) gene disrupts mating in the Fall armyworm, Spodoptera frugiperda (J. E. Smith) (Lepidoptera: Noctuidae). 3 Biotech. 2023 Nov;13(11):370.

37. Atarashi H, Ogawa S, Inoue H, Hamada C. Dose-response effect of flecainide in patients with symptomatic paroxysmal atrial fibrillation and/or flutter monitored with trans-telephonic electrocardiography: a multicenter, placebo-controlled, double-blind trial. Circ J. 2007 Mar;71(3):294-300.

38. Ay U, Yildirim Z, Erdogdu E, Kicik A, Ozturk-Isik E, Demiralp T, et al. Shrinkage of olfactory amygdala connotes cognitive impairment in patients with Parkinson's disease. Cogn Neurodyn. 2023 Oct;17(5):1309-20.

39. Baek S, Ha Y, Park HW. Accuracy of Wearable Devices for Measuring Heart Rate During Conventional and Nordic Walking. Pm r. 2021 Apr;13(4):379-86.

40. Bai Y, Hibbing P, Mantis C, Welk GJ. Comparative evaluation of heart rate-based monitors: Apple Watch vs Fitbit Charge HR. J Sports Sci. 2018 Aug;36(15):1734-41.

41. Bai Y, Tompkins C, Gell N, Dione D, Zhang T, Byun W. Comprehensive comparison of Apple Watch and Fitbit monitors in a free-living setting. PLoS One. 2021;16(5):e0251975.

42. Bai Y, Welk GJ, Nam YH, Lee JA, Lee JM, Kim Y, et al. Comparison of Consumer and Research Monitors under Semistructured Settings. Med Sci Sports Exerc. 2016 Jan;48(1):151-8.

43. Bai Z, Lyu X, Tang Y, Wang M. Pediatric Tui Na for Feeding Intolerance in Premature Infants: Protocol for a Systematic Review and Meta-Analysis. JMIR Res Protoc. 2023 Oct 16;12:e46375.

44. Balto JM, Kinnett-Hopkins DL, Motl RW. Accuracy and precision of smartphone applications and commercially available motion sensors in multiple sclerosis. Mult Scler J Exp Transl Clin. 2016 Jan-Dec;2:2055217316634754.

45. Bashar SK, Han D, Ding E, Whitcomb C, McManus DD, Chon KH. Smartwatch Based Atrial Fibrillation Detection from Photoplethysmography Signals. Annu Int Conf IEEE Eng Med Biol Soc. 2019 Jul;2019:4306-9.

46. Bashar SK, Han D, Hajeb-Mohammadalipour S, Ding E, Whitcomb C, McManus DD, et al. Atrial Fibrillation Detection from Wrist Photoplethysmography Signals Using Smartwatches. Sci Rep. 2019 Oct 21;9(1):15054.

47. Beattie Z, Oyang Y, Statan A, Ghoreyshi A, Pantelopoulos A, Russell A, et al. Estimation of sleep stages in a healthy adult population from optical plethysmography and accelerometer signals. Physiol Meas. 2017 Oct 31;38(11):1968-79.

48. Beets MW, Combs C, Pitetti KH, Morgan M, Bryan RR, Foley JT. Accuracy of pedometer steps and time for youth with disabilities. Adapt Phys Activ Q. 2007 Jul;24(3):228-44.

49. Beevi FH, Miranda J, Pedersen CF, Wagner S. An Evaluation of Commercial Pedometers for Monitoring Slow Walking Speed Populations. Telemed J E Health. 2016 May;22(5):441-9.

50. Benedetto S, Caldato C, Bazzan E, Greenwood DC, Pensabene V, Actis P. Assessment of the Fitbit Charge 2 for monitoring heart rate. PLoS One. 2018;13(2):e0192691.

51. Benito PJ, Neiva C, Gonzalez-Quijano PS, Cupeiro R, Morencos E, Peinado AB. Validation of the SenseWear armband in circuit resistance training with different loads. Eur J Appl Physiol. 2012 Aug;112(8):3155-9.

52. Benning N-H, Knaup P, Rupp R. Comparison of accuracy of activity measurements with wearable activity trackers in wheelchair users: a preliminary evaluation. GMS Med Inf Biom Epidemiol. 2020;16(02):Doc05.

53. Benning NH, Knaup P, Rupp R. Measurement Performance of Activity Measurements with Newer Generation of Apple Watch in Wheelchair Users with Spinal Cord Injury. Methods Inf Med. 2021 Dec;60(S 02):e103-e10.

54. Bent B, Goldstein BA, Kibbe WA, Dunn JP. Investigating sources of inaccuracy in wearable optical heart rate sensors. NPJ Digit Med. 2020;3:18.

55. Berntsen S, Hageberg R, Aandstad A, Mowinckel P, Anderssen SA, Carlsen KH, et al. Validity of physical activity monitors in adults participating in free-living activities. Br J Sports Med. 2010 Jul;44(9):657-64.

56. Berntsen S, Stafne SN, Morkved S. Physical activity monitor for recording energy expenditure in pregnancy. Acta Obstet Gynecol Scand. 2011 Aug;90(8):903-7.

57. Bhammar DM, Sawyer BJ, Tucker WJ, Lee JM, Gaesser GA. Validity of SenseWear(R) Armband v5.2 and v2.2 for estimating energy expenditure. J Sports Sci. 2016 Oct;34(19):1830-8.

58. Boeselt T, Spielmanns M, Nell C, Storre JH, Windisch W, Magerhans L, et al. Validity and Usability of Physical Activity Monitoring in Patients with Chronic Obstructive Pulmonary Disease (COPD). PLoS One. 2016;11(6):e0157229.

59. Bonomi AG, Schipper F, Eerikainen LM, Margarito J, van Dinther R, Muesch G, et al. Atrial Fibrillation Detection Using a Novel Cardiac Ambulatory Monitor Based on Photo-Plethysmography at the Wrist. J Am Heart Assoc. 2018 Aug 7;7(15):e009351.

60. Boudreaux BD, Hebert EP, Hollander DB, Williams BM, Cormier CL, Naquin MR, et al. Validity of Wearable Activity Monitors during Cycling and Resistance Exercise. Med Sci Sports Exerc. 2018 Mar;50(3):624-33.

61. Brasier N, Raichle CJ, Dorr M, Becke A, Nohturfft V, Weber S, et al. Detection of atrial fibrillation with a smartphone camera: first prospective, international, two-centre, clinical validation study (DETECT AF PRO). Europace. 2019 Jan 1;21(1):41-7.

62. Brazeau AS, Beaudoin N, Belisle V, Messier V, Karelis AD, Rabasa-Lhoret R. Validation and reliability of two activity monitors for energy expenditure assessment. J Sci Med Sport. 2016 Jan;19(1):46-50.

63. Brazeau AS, Karelis AD, Mignault D, Lacroix MJ, Prud'homme D, Rabasa-Lhoret R. Accuracy of the SenseWear Armband during ergocycling. Int J Sports Med. 2011 Oct;32(10):761-4.

64. Brazeau AS, Suppere C, Strychar I, Belisle V, Demers SP, Rabasa-Lhoret R. Accuracy of energy expenditure estimation by activity monitors differs with ethnicity. Int J Sports Med. 2014 Sep;35(10):847-50.

65. Brazendale K, Beets MW, Weaver RG, Perry MW, Tyler EB, Hunt ET, et al. Comparing measures of free-living sleep in school-aged children. Sleep Med. 2019 Aug;60:197-201.

66. Brewer W, Swanson BT, Ortiz A. Validity of Fitbit's active minutes as compared with a research-grade accelerometer and self-reported measures. BMJ Open Sport Exerc Med. 2017;3(1):e000254.

67. Brooke SM, An HS, Kang SK, Noble JM, Berg KE, Lee JM. Concurrent Validity of Wearable Activity Trackers Under Free-Living Conditions. J Strength Cond Res. 2017 Apr;31(4):1097-106.

68. Bruder AM, McClelland JA, Shields N, Dodd KJ, Hau R, van de Water ATM, et al. Validity and reliability of an activity monitor to quantify arm movements and activity in adults following distal radius fracture. Disabil Rehabil. 2018 Jun;40(11):1318-25.

69. Brugniaux JV, Niva A, Pulkkinen I, Laukkanen RM, Richalet JP, Pichon AP. Polar Activity Watch 200: a new device to accurately assess energy expenditure. Br J Sports Med. 2010 Mar;44(4):245-9.

70. Brunetti ND, De Gennaro L, Pellegrino PL, Dellegrottaglie G, Antonelli G, Di Biase M. Atrial fibrillation with symptoms other than palpitations: incremental diagnostic sensitivity with at-home tele-cardiology assessment for emergency medical service. Eur J Prev Cardiol. 2012 Jun;19(3):306-13.

71. Bumgarner JM, Lambert CT, Hussein AA, Cantillon DJ, Baranowski B, Wolski K, et al. Smartwatch Algorithm for Automated Detection of Atrial Fibrillation. J Am Coll Cardiol. 2018 May 29;71(21):2381-8.

72. Bunn J, Wells E, Manor J, Webster M. Evaluation of Earbud and Wristwatch Heart Rate Monitors during Aerobic and Resistance Training. Int J Exerc Sci. 2019;12(4):374-84.

73. Bunn JA, Jones C, Oliviera A, Webster MJ. Assessment of step accuracy using the Consumer Technology Association standard. J Sports Sci. 2019 Feb;37(3):244-8.

74. Burton E, Hill KD, Lautenschlager NT, Thogersen-Ntoumani C, Lewin G, Boyle E, et al. Reliability and validity of two fitness tracker devices in the laboratory and home environment for older community-dwelling people. BMC Geriatr. 2018 May 3;18(1):103.

75. Cadmus-Bertram L, Gangnon R, Wirkus EJ, Thraen-Borowski KM, Gorzelitz-Liebhauser J. The Accuracy of Heart Rate Monitoring by Some Wrist-Worn Activity Trackers. Ann Intern Med. 2017 Apr 18;166(8):610-2.

76. Caillol T, Strik M, Ramirez FD, Abu-Alrub S, Marchand H, Buliard S, et al. Accuracy of a Smartwatch-Derived ECG for Diagnosing Bradyarrhythmias, Tachyarrhythmias, and Cardiac Ischemia. Circ Arrhythm Electrophysiol. 2021 Jan;14(1):e009260.

77. Calabro MA, Kim Y, Franke WD, Stewart JM, Welk GJ. Objective and subjective measurement of energy expenditure in older adults: a doubly labeled water study. Eur J Clin Nutr. 2015 Jul;69(7):850-5.

78. Calabro MA, Lee JM, Saint-Maurice PF, Yoo H, Welk GJ. Validity of physical activity monitors for assessing lower intensity activity in adults. Int J Behav Nutr Phys Act. 2014 Sep 28;11:119.

79. Case MA, Burwick HA, Volpp KG, Patel MS. Accuracy of smartphone applications and wearable devices for tracking physical activity data. Jama. 2015 Feb 10;313(6):625-6.

80. Casiraghi F, Lertwattanarak R, Luzi L, Chavez AO, Davalli AM, Naegelin T, et al. Energy expenditure evaluation in humans and non-human primates by SenseWear Armband. Validation of energy expenditure evaluation by SenseWear Armband by direct comparison with indirect calorimetry. PLoS One. 2013;8(9):e73651.

81. Chan PH, Wong CK, Pun L, Wong YF, Wong MM, Chu DW, et al. Head-to-Head Comparison of the AliveCor Heart Monitor and Microlife WatchBP Office AFIB for Atrial Fibrillation Screening in a Primary Care Setting. Circulation. 2017 Jan 3;135(1):110-2.

82. Chandrasekar A, Hensor EMA, Mackie SL, Backhouse MR, Harris E. Preliminary concurrent validity of the Fitbit-Zip and ActiGraph activity monitors for measuring steps in people with polymyalgia rheumatica. Gait Posture. 2018 Mar;61:339-45.

83. Chen E, Jiang J, Su R, Gao M, Zhu S, Zhou J, et al. A new smart wristband equipped with an artificial intelligence algorithm to detect atrial fibrillation. Heart Rhythm. 2020 May;17(5 Pt B):847-53.

84. Chen MD, Kuo CC, Pellegrini CA, Hsu MJ. Accuracy of Wristband Activity Monitors during Ambulation and Activities. Med Sci Sports Exerc. 2016 Oct;48(10):1942-9.

85. Choi L, Chen KY, Acra SA, Buchowski MS. Distributed lag and spline modeling for predicting energy expenditure from accelerometry in youth. J Appl Physiol (1985). 2010 Feb;108(2):314-27.

86. Chow JJ, Thom JM, Wewege MA, Ward RE, Parmenter BJ. Accuracy of step count measured by physical activity monitors: The effect of gait speed and anatomical placement site. Gait Posture. 2017 Sep;57:199-203.

87. Chowdhury EA, Western MJ, Nightingale TE, Peacock OJ, Thompson D. Assessment of laboratory and daily energy expenditure estimates from consumer multi-sensor physical activity monitors. PLoS One. 2017;12(2):e0171720.

88. Chu AH, Ng SH, Paknezhad M, Gauterin A, Koh D, Brown MS, et al. Comparison of wrist-worn Fitbit Flex and waist-worn ActiGraph for measuring steps in free-living adults. PLoS One. 2017;12(2):e0172535.

89. Claes J, Buys R, Avila A, Finlay D, Kennedy A, Guldenring D, et al. Validity of heart rate measurements by the Garmin Forerunner 225 at different walking intensities. J Med Eng Technol. 2017 Aug;41(6):480-5.

90. Cohen MD, Cutaia M. A novel approach to measuring activity in chronic obstructive pulmonary disease: using 2 activity monitors to classify daily activity. J Cardiopulm Rehabil Prev. 2010 May-Jun;30(3):186-94.

91. Compagnat M, Mandigout S, Chaparro D, Daviet JC, Salle JY. Validity of the Actigraph GT3x and influence of the sensor positioning for the assessment of active energy expenditure during four activities of daily living in stroke subjects. Clin Rehabil. 2018 Dec;32(12):1696-704.

92. Conroy T, Guzman JH, Hall B, Tsouri G, Couderc JP. Detection of atrial fibrillation using an earlobe photoplethysmographic sensor. Physiol Meas. 2017 Sep 26;38(10):1906-18.

93. Cook JD, Eftekari SC, Dallmann E, Sippy M, Plante DT. Ability of the Fitbit Alta HR to quantify and classify sleep in patients with suspected central disorders of hypersomnolence: A comparison against polysomnography. J Sleep Res. 2019 Aug;28(4):e12789.

94. Cook JD, Prairie ML, Plante DT. Utility of the Fitbit Flex to evaluate sleep in major depressive disorder: A comparison against polysomnography and wrist-worn actigraphy. J Affect Disord. 2017 Aug 1;217:299-305.

95. Cooper KD, Shafer AB. Validity and Reliability of the Polar A300's Fitness Test Feature to Predict VO2max. Int J Exerc Sci. 2019;12(4):393-401.

96. Corino VDA, Laureanti R, Ferranti L, Scarpini G, Lombardi F, Mainardi LT. Detection of atrial fibrillation episodes using a wristband device. Physiol Meas. 2017 May;38(5):787-99.

97. Correa JB, Apolzan JW, Shepard DN, Heil DP, Rood JC, Martin CK. Evaluation of the ability of three physical activity monitors to predict weight change and estimate energy expenditure. Appl Physiol Nutr Metab. 2016 Jul;41(7):758-66.

98. Crouter SE, Albright C, Bassett DR, Jr. Accuracy of polar S410 heart rate monitor to estimate energy cost of exercise. Med Sci Sports Exerc. 2004 Aug;36(8):1433-9.

99. Dannecker KL, Sazonova NA, Melanson EL, Sazonov ES, Browning RC. A comparison of energy expenditure estimation of several physical activity monitors. Med Sci Sports Exerc. 2013 Nov;45(11):2105-12.

100. de Rezende Barbosa MPC, Junior JN, Cassemiro BM, Bernardo AFB, Franca da Silva AK, Vanderlei FM, et al. Effects of functional training on geometric indices of heart rate variability. J Sport Health Sci. 2016 Jun;5(2):183-9.

101. de Zambotti M, Baker FC, Colrain IM. Validation of Sleep-Tracking Technology Compared with Polysomnography in Adolescents. Sleep. 2015 Sep 1;38(9):1461-8.

102. de Zambotti M, Baker FC, Willoughby AR, Godino JG, Wing D, Patrick K, et al. Measures of sleep and cardiac functioning during sleep using a multi-sensory commercially-available wristband in adolescents. Physiol Behav. 2016 May 1;158:143-9.

103. de Zambotti M, Claudatos S, Inkelis S, Colrain IM, Baker FC. Evaluation of a consumer fitness-tracking device to assess sleep in adults. Chronobiol Int. 2015;32(7):1024-8.

104. de Zambotti M, Goldstone A, Claudatos S, Colrain IM, Baker FC. A validation study of Fitbit Charge 2 compared with polysomnography in adults. Chronobiol Int. 2018 Apr;35(4):465-76.

105. Degroote L, De Bourdeaudhuij I, Verloigne M, Poppe L, Crombez G. The Accuracy of Smart Devices for Measuring Physical Activity in Daily Life: Validation Study. JMIR Mhealth Uhealth. 2018 Dec 13;6(12):e10972.

106. Deka P, Pozehl B, Norman JF, Khazanchi D. Feasibility of using the Fitbit((R)) Charge HR in validating self-reported exercise diaries in a community setting in patients with heart failure. Eur J Cardiovasc Nurs. 2018 Oct;17(7):605-11.

107. Desteghe L, Raymaekers Z, Lutin M, Vijgen J, Dilling-Boer D, Koopman P, et al. Performance of handheld electrocardiogram devices to detect atrial fibrillation in a cardiology and geriatric ward setting. Europace. 2017 Jan;19(1):29-39.

108. Diaz KM, Krupka DJ, Chang MJ, Peacock J, Ma Y, Goldsmith J, et al. Fitbit(R): An accurate and reliable device for wireless physical activity tracking. Int J Cardiol. 2015 Apr 15;185:138-40.

109. Diaz KM, Krupka DJ, Chang MJ, Shaffer JA, Ma Y, Goldsmith J, et al. Validation of the Fitbit One(R) for physical activity measurement at an upper torso attachment site. BMC Res Notes. 2016 Apr 12;9:213.

110. Dickinson DL, Cazier J, Cech T. A practical validation study of a commercial accelerometer using good and poor sleepers. Health Psychol Open. 2016 Jul;3(2):2055102916679012.

111. Dijkstra B, Zijlstra W, Scherder E, Kamsma Y. Detection of walking periods and number of steps in older adults and patients with Parkinson's disease: accuracy of a pedometer and an accelerometry-based method. Age Ageing. 2008 Jul;37(4):436-41.

112. Ding EY, Han D, Whitcomb C, Bashar SK, Adaramola O, Soni A, et al. Accuracy and Usability of a Novel Algorithm for Detection of Irregular Pulse Using a Smartwatch Among Older Adults: Observational Study. JMIR Cardio. 2019 May 15;3(1):e13850.

113. Doheny EP, Renerts K, Baumann C, Morgan-Jones P, Busse M, Lowery MM, et al. F35 Sleep monitoring in huntington’s disease using fitbit compared to polysomnography. Journal of Neurology, Neurosurgery &amp; Psychiatry. 2021;92(Suppl 1):A33-A4.

114. Dominick GM, Winfree KN, Pohlig RT, Papas MA. Physical Activity Assessment Between Consumer- and Research-Grade Accelerometers: A Comparative Study in Free-Living Conditions. JMIR Mhealth Uhealth. 2016 Sep 19;4(3):e110.

115. Dondzila C, Garner D. Comparative accuracy of fitness tracking modalities in quantifying energy expenditure. J Med Eng Technol. 2016 Aug;40(6):325-9.

116. Dondzila CJ, Lewis C, López JR, Parker TM. Congruent Accuracy of Wrist-worn Activity Trackers during Controlled and Free-living Conditions. International journal of exercise science. 2018;11:575-84.

117. Dong X, Yang S, Guo Y, Lv P, Wang M, Li Y. Validation of Fitbit Charge 4 for assessing sleep in Chinese patients with chronic insomnia: A comparison against polysomnography and actigraphy. PLoS One. 2022;17(10):e0275287.

118. Dontje ML, de Groot M, Lengton RR, van der Schans CP, Krijnen WP. Measuring steps with the Fitbit activity tracker: an inter-device reliability study. J Med Eng Technol. 2015;39(5):286-90.

119. Dooley EE, Golaszewski NM, Bartholomew JB. Estimating Accuracy at Exercise Intensities: A Comparative Study of Self-Monitoring Heart Rate and Physical Activity Wearable Devices. JMIR Mhealth Uhealth. 2017 Mar 16;5(3):e34.

120. Dorr M, Nohturfft V, Brasier N, Bosshard E, Djurdjevic A, Gross S, et al. The WATCH AF Trial: SmartWATCHes for Detection of Atrial Fibrillation. JACC Clin Electrophysiol. 2019 Feb;5(2):199-208.

121. Dudek NL, Khan OD, Lemaire ED, Marks MB, Saville L. Ambulation monitoring of transtibial amputation subjects with patient activity monitor versus pedometer. J Rehabil Res Dev. 2008;45(4):577-85.

122. Duking P, Giessing L, Frenkel MO, Koehler K, Holmberg HC, Sperlich B. Wrist-Worn Wearables for Monitoring Heart Rate and Energy Expenditure While Sitting or Performing Light-to-Vigorous Physical Activity: Validation Study. JMIR Mhealth Uhealth. 2020 May 6;8(5):e16716.

123. Dur O, Rhoades C, Ng MS, Elsayed R, van Mourik R, Majmudar MD. Design Rationale and Performance Evaluation of the Wavelet Health Wristband: Benchtop Validation of a Wrist-Worn Physiological Signal Recorder. JMIR Mhealth Uhealth. 2018 Oct 16;6(10):e11040.

124. Eerikäinen LM, Dekker L, Bonomi AG, Vullings R, Schipper F, Margarito J, et al. Validating features for atrial fibrillation detection from photoplethysmogram under hospital and free-living conditions. 2017 Computing in Cardiology (CinC); 2017 24-27 Sept. 2017; 2017. p. 1-4.

125. Ehrler F, Weber C, Lovis C. Influence of Pedometer Position on Pedometer Accuracy at Various Walking Speeds: A Comparative Study. J Med Internet Res. 2016 Oct 6;18(10):e268.

126. Elsworth C, Dawes H, Winward C, Howells K, Collett J, Dennis A, et al. Pedometer step counts in individuals with neurological conditions. Clin Rehabil. 2009 Feb;23(2):171-5.

127. Eriksson MJ, Sonnenberg B, Woo A, Rakowski P, Parker TG, Wigle ED, et al. Long-term outcome in patients with apical hypertrophic cardiomyopathy. J Am Coll Cardiol. 2002 Feb 20;39(4):638-45.

128. Esco M, Mugu E, Williford H, McHugh A, Bloomquist B. Cross-Validation of the Polar Fitness TestTM via the Polar F11 Heart Rate Monitor in Predicting VO2max. Journal of Exercise Physiology. 2011 10/01;14:43-52.

129. Esco MR, Flatt AA, Nakamura FY. Agreement Between a Smartphone Pulse Sensor Application and Electrocardiography for Determining lnRMSSD. J Strength Cond Res. 2017 Feb;31(2):380-5.

130. Esco MR, Snarr RL, Williford HN. Monitoring changes in VO2max via the Polar FT40 in female collegiate soccer players. J Sports Sci. 2014;32(11):1084-90.

131. Etiwy M, Akhrass Z, Gillinov L, Alashi A, Wang R, Blackburn G, et al. Accuracy of wearable heart rate monitors in cardiac rehabilitation. Cardiovasc Diagn Ther. 2019 Jun;9(3):262-71.

132. Falter M, Budts W, Goetschalckx K, Cornelissen V, Buys R. Accuracy of Apple Watch Measurements for Heart Rate and Energy Expenditure in Patients With Cardiovascular Disease: Cross-Sectional Study. JMIR Mhealth Uhealth. 2019 Mar 19;7(3):e11889.

133. Fan YY, Li YG, Li J, Cheng WK, Shan ZL, Wang YT, et al. Diagnostic Performance of a Smart Device With Photoplethysmography Technology for Atrial Fibrillation Detection: Pilot Study (Pre-mAFA II Registry). JMIR Mhealth Uhealth. 2019 Mar 5;7(3):e11437.

134. Farina N, Lowry RG. The Validity of Consumer-Level Activity Monitors in Healthy Older Adults in Free-Living Conditions. J Aging Phys Act. 2018 Jan 1;26(1):128-35.

135. Farina N, Sherlock G, Thomas S, Lowry RG, Banerjee S. Acceptability and feasibility of wearing activity monitors in community-dwelling older adults with dementia. Int J Geriatr Psychiatry. 2019 Apr;34(4):617-24.

136. Ferguson T, Rowlands AV, Olds T, Maher C. The validity of consumer-level, activity monitors in healthy adults worn in free-living conditions: a cross-sectional study. Int J Behav Nutr Phys Act. 2015 Mar 27;12:42.

137. Fisher JM, Hammerla NY, Rochester L, Andras P, Walker RW. Body-Worn Sensors in Parkinson's Disease: Evaluating Their Acceptability to Patients. Telemed J E Health. 2016 Jan;22(1):63-9.

138. Flatt AA, Esco MR. Validity of the ithlete Smart Phone Application for Determining Ultra-Short-Term Heart Rate Variability. J Hum Kinet. 2013 Dec 18;39:85-92.

139. Floegel TA, Florez-Pregonero A, Hekler EB, Buman MP. Validation of Consumer-Based Hip and Wrist Activity Monitors in Older Adults With Varied Ambulatory Abilities. J Gerontol A Biol Sci Med Sci. 2017 Feb;72(2):229-36.

140. Fokkema T, Kooiman TJ, Krijnen WP, CP VDS, M DEG. Reliability and Validity of Ten Consumer Activity Trackers Depend on Walking Speed. Med Sci Sports Exerc. 2017 Apr;49(4):793-800.

141. Fournier CN, Glass JD. The importance of understanding minimal important difference for ALSFRS-R: A reply to 'Do we really need to calculate a minimal important difference for ALSFRS-R?' <https://doi.org/10.1080/21678421.2023.2248199>. Amyotroph Lateral Scler Frontotemporal Degener. 2023 Sep 20:1-2.

142. Freeberg KA, Baughman BR, Vickey T, Sullivan JA, Sawyer BJ. Assessing the ability of the Fitbit Charge 2 to accurately predict VO(2max). Mhealth. 2019;5:39.

143. Fruin ML, Rankin JW. Validity of a multi-sensor armband in estimating rest and exercise energy expenditure. Med Sci Sports Exerc. 2004 Jun;36(6):1063-9.

144. Fukuma N, Hasumi E, Fujiu K, Waki K, Toyooka T, Komuro I, et al. Feasibility of a T-Shirt-Type Wearable Electrocardiography Monitor for Detection of Covert Atrial Fibrillation in Young Healthy Adults. Sci Rep. 2019 Aug 13;9(1):11768.

145. Fulk GD, Combs SA, Danks KA, Nirider CD, Raja B, Reisman DS. Accuracy of 2 activity monitors in detecting steps in people with stroke and traumatic brain injury. Phys Ther. 2014 Feb;94(2):222-9.

146. Furlanetto KC, Bisca GW, Oldemberg N, Sant'anna TJ, Morakami FK, Camillo CA, et al. Step counting and energy expenditure estimation in patients with chronic obstructive pulmonary disease and healthy elderly: accuracy of 2 motion sensors. Arch Phys Med Rehabil. 2010 Feb;91(2):261-7.

147. Gamelin FX, Baquet G, Berthoin S, Bosquet L. Validity of the polar S810 to measure R-R intervals in children. Int J Sports Med. 2008 Feb;29(2):134-8.

148. Gamelin FX, Berthoin S, Bosquet L. Validity of the polar S810 heart rate monitor to measure R-R intervals at rest. Med Sci Sports Exerc. 2006 May;38(5):887-93.

149. Gandolfo C, Balestrino M, Bruno C, Finocchi C, Reale N. Validation of a simple method for atrial fibrillation screening in patients with stroke. Neurol Sci. 2015 Sep;36(9):1675-8.

150. Gastin PB, Cayzer C, Dwyer D, Robertson S. Validity of the ActiGraph GT3X+ and BodyMedia SenseWear Armband to estimate energy expenditure during physical activity and sport. J Sci Med Sport. 2018 Mar;21(3):291-5.

151. Gaynor M, Sawyer A, Jenkins S, Wood J. Variable agreement between wearable heart rate monitors during exercise in cystic fibrosis. ERJ Open Res. 2019 Oct;5(4).

152. Gaz DV, Rieck TM, Peterson NW, Ferguson JA, Schroeder DR, Dunfee HA, et al. Determining the Validity and Accuracy of Multiple Activity-Tracking Devices in Controlled and Free-Walking Conditions. Am J Health Promot. 2018 Nov;32(8):1671-8.

153. Giles D, Draper N, Neil W. Validity of the Polar V800 heart rate monitor to measure RR intervals at rest. Eur J Appl Physiol. 2016 Mar;116(3):563-71.

154. Gillinov S, Etiwy M, Wang R, Blackburn G, Phelan D, Gillinov AM, et al. Variable Accuracy of Wearable Heart Rate Monitors during Aerobic Exercise. Med Sci Sports Exerc. 2017 Aug;49(8):1697-703.

155. Gironda RJ, Lloyd J, Clark ME, Walker RL. Preliminary evaluation of reliability and criterion validity of Actiwatch-Score. J Rehabil Res Dev. 2007;44(2):223-30.

156. Glasheen E, Domingo A, Kressler J. Accuracy of Apple Watch fitness tracker for wheelchair use varies according to movement frequency and task. Annals of Physical and Rehabilitation Medicine. 2021 2021/01/01/;64(1):101382.

157. Glasheen E, Domingo A, Moreno D, Kressler J. Validity of Apple Watch Fitness Tracker for Wheelchair Users. Archives of Physical Medicine and Rehabilitation. 2017 2017/12/01/;98(12):e178.

158. Godinho MR, Greco RM, Teixeira MT, Teixeira LR, Guerra MR, Chaoubah A. Work ability and associated factors of Brazilian technical-administrative workers in education. BMC Res Notes. 2016 Jan 2;9:1.

159. Gomersall SR, Ng N, Burton NW, Pavey TG, Gilson ND, Brown WJ. Estimating Physical Activity and Sedentary Behavior in a Free-Living Context: A Pragmatic Comparison of Consumer-Based Activity Trackers and ActiGraph Accelerometry. J Med Internet Res. 2016 Sep 7;18(9):e239.

160. Gorny AW, Liew SJ, Tan CS, Muller-Riemenschneider F. Fitbit Charge HR Wireless Heart Rate Monitor: Validation Study Conducted Under Free-Living Conditions. JMIR Mhealth Uhealth. 2017 Oct 20;5(10):e157.

161. Gruwez A, Libert W, Ameye L, Bruyneel M. Reliability of commercially available sleep and activity trackers with manual switch-to-sleep mode activation in free-living healthy individuals. Int J Med Inform. 2017 Jun;102:87-92.

162. Gunnarsson GL, Lilja C, Thomsen JB. Breast reconstruction with donor tissue from the back-an evolution towards customized reconstructive solutions guided by innovative surgery and patient wishes. Gland Surg. 2023 Sep 25;12(9):1137-40.

163. Guo Y, Wang H, Zhang H, Liu T, Liang Z, Xia Y, et al. Mobile Photoplethysmographic Technology to Detect Atrial Fibrillation. J Am Coll Cardiol. 2019 Nov 12;74(19):2365-75.

164. Haberman ZC, Jahn RT, Bose R, Tun H, Shinbane JS, Doshi RN, et al. Wireless Smartphone ECG Enables Large-Scale Screening in Diverse Populations. J Cardiovasc Electrophysiol. 2015 May;26(5):520-6.

165. Haegele JA, Brian AS, Wolf D. Accuracy of the Fitbit Zip for Measuring Steps for Adolescents With Visual Impairments. Adapt Phys Activ Q. 2017 Apr;34(2):195-200.

166. Haghayegh S, Khoshnevis S, Smolensky MH, Diller KR. Accuracy of PurePulse photoplethysmography technology of Fitbit Charge 2 for assessment of heart rate during sleep. Chronobiol Int. 2019 Jul;36(7):927-33.

167. Hakim M, Miller R, Hakim M, Tumin D, Tobias JD, Jatana KR, et al. Comparison of the Fitbit(R) charge and polysomnography for measuring sleep quality in children with sleep disordered breathing. Minerva Pediatr (Torino). 2022 Jun;74(3):259-63.

168. Han D, Bashar SK, Mohagheghian F, Ding E, Whitcomb C, McManus DD, et al. Premature Atrial and Ventricular Contraction Detection using Photoplethysmographic Data from a Smartwatch. Sensors (Basel). 2020 Oct 5;20(19).

169. Hargens TA, Deyarmin KN, Snyder KM, Mihalik AG, Sharpe LE. Comparison of wrist-worn and hip-worn activity monitors under free living conditions. J Med Eng Technol. 2017 Apr;41(3):200-7.

170. Harju J, Tarniceriu A, Parak J, Vehkaoja A, Yli-Hankala A, Korhonen I. Monitoring of heart rate and inter-beat intervals with wrist plethysmography in patients with atrial fibrillation. Physiol Meas. 2018 Jun 27;39(6):065007.

171. Heathers JA. Smartphone-enabled pulse rate variability: an alternative methodology for the collection of heart rate variability in psychophysiological research. Int J Psychophysiol. 2013 Sep;89(3):297-304.

172. Heiermann S, Khalaj Hedayati K, Muller MJ, Dittmar M. Accuracy of a portable multisensor body monitor for predicting resting energy expenditure in older people: a comparison with indirect calorimetry. Gerontology. 2011;57(5):473-9.

173. Hendrikx J, Ruijs LS, Cox LG, Lemmens PM, Schuijers EG, Goris AH. Clinical Evaluation of the Measurement Performance of the Philips Health Watch: A Within-Person Comparative Study. JMIR Mhealth Uhealth. 2017 Feb 2;5(2):e10.

174. Henriksen A, Grimsgaard S, Horsch A, Hartvigsen G, Hopstock L. Validity of the Polar M430 Activity Monitor in Free-Living Conditions: Validation Study. JMIR Form Res. 2019 Aug 16;3(3):e14438.

175. Heo NJ, Rhee SY, Waalen J, Steinhubl S. Chronic kidney disease and undiagnosed atrial fibrillation in individuals with diabetes. Cardiovasc Diabetol. 2020 Sep 30;19(1):157.

176. Hergenroeder AL, Barone Gibbs B, Kotlarczyk MP, Perera S, Kowalsky RJ, Brach JS. Accuracy and Acceptability of Commercial-Grade Physical Activity Monitors in Older Adults. J Aging Phys Act. 2019 Apr 1;27(2):222-9.

177. Hermand E, Cassirame J, Ennequin G, Hue O. Validation of a Photoplethysmographic Heart Rate Monitor: Polar OH1. Int J Sports Med. 2019 Jul;40(7):462-7.

178. Hernandez-Vicente A, Santos-Lozano A, De Cocker K, Garatachea N. Validation study of Polar V800 accelerometer. Ann Transl Med. 2016 Aug;4(15):278.

179. Hernando D, Garatachea N, Almeida R, Casajus JA, Bailon R. Validation of Heart Rate Monitor Polar RS800 for Heart Rate Variability Analysis During Exercise. J Strength Cond Res. 2018 Mar;32(3):716-25.

180. Hochsmann C, Knaier R, Eymann J, Hintermann J, Infanger D, Schmidt-Trucksass A. Validity of activity trackers, smartphones, and phone applications to measure steps in various walking conditions. Scand J Med Sci Sports. 2018 Jul;28(7):1818-27.

181. Hochstadt A, Chorin E, Viskin S, Schwartz AL, Lubman N, Rosso R. Continuous heart rate monitoring for automatic detection of atrial fibrillation with novel bio-sensing technology. J Electrocardiol. 2019 Jan-Feb;52:23-7.

182. Hochstadt A, Havakuk O, Chorin E, Schwartz AL, Merdler I, Laufer M, et al. Continuous heart rhythm monitoring using mobile photoplethysmography in ambulatory patients. J Electrocardiol. 2020 May-Jun;60:138-41.

183. Hojager A, Tingsgaard JK, Andersen D, Soholm H, Taskiran M, Bock TG, et al. Silent atrial fibrillation detected by home-monitoring: Cardiovascular disease and stroke prevention in patients with diabetes. J Diabetes Complications. 2020 Dec;34(12):107711.

184. Hong S, Yang Y, Kim S, Shin S, Lee I, Jang Y, et al. Performance study of the wearable one-lead wireless electrocardiographic monitoring system. Telemed J E Health. 2009 Mar;15(2):166-75.

185. Horton JF, Stergiou P, Fung TS, Katz L. Comparison of Polar M600 Optical Heart Rate and ECG Heart Rate during Exercise. Med Sci Sports Exerc. 2017 Dec;49(12):2600-7.

186. Hough P, Glaister M, Pledger A. The Accuracy of Wrist-worn Heart Rate Monitors across a Range of Exercise Intensities. Journal of Physical Activity Research. 2017;2(2):112-6.

187. Huang Y, Xu J, Yu B, Shull PB. Validity of FitBit, Jawbone UP, Nike+ and other wearable devices for level and stair walking. Gait Posture. 2016 Jul;48:36-41.

188. Hui J, Heyden R, Bao T, Accettone N, McBay C, Richardson J, et al. Validity of the Fitbit One for Measuring Activity in Community-Dwelling Stroke Survivors. Physiother Can. 2018;70(1):81-9.

189. Huynh P, Shan R, Osuji N, Ding J, Isakadze N, Marvel FA, et al. Heart Rate Measurements in Patients with Obstructive Sleep Apnea and Atrial Fibrillation: Prospective Pilot Study Assessing Apple Watch's Agreement With Telemetry Data. JMIR Cardio. 2021 Feb 8;5(1):e18050.

190. Hwang J, Kim J, Choi KJ, Cho MS, Nam GB, Kim YH. Assessing Accuracy of Wrist-Worn Wearable Devices in Measurement of Paroxysmal Supraventricular Tachycardia Heart Rate. Korean Circ J. 2019 May;49(5):437-45.

191. Imboden MT, Nelson MB, Kaminsky LA, Montoye AH. Comparison of four Fitbit and Jawbone activity monitors with a research-grade ActiGraph accelerometer for estimating physical activity and energy expenditure. Br J Sports Med. 2018 Jul;52(13):844-50.

192. Inui T, Kohno H, Kawasaki Y, Matsuura K, Ueda H, Tamura Y, et al. Use of a Smart Watch for Early Detection of Paroxysmal Atrial Fibrillation: Validation Study. JMIR Cardio. 2020 Jan 22;4(1):e14857.

193. Jaakkola J, Jaakkola S, Lahdenoja O, Hurnanen T, Koivisto T, Pankaala M, et al. Mobile Phone Detection of Atrial Fibrillation With Mechanocardiography: The MODE-AF Study (Mobile Phone Detection of Atrial Fibrillation). Circulation. 2018 Apr 3;137(14):1524-7.

194. Jacobsen M, Dembek TA, Ziakos AP, Gholamipoor R, Kobbe G, Kollmann M, et al. Reliable Detection of Atrial Fibrillation with a Medical Wearable during Inpatient Conditions. Sensors (Basel). 2020 Sep 26;20(19).

195. Jagim AR, Koch-Gallup N, Camic CL, Kroening L, Nolte C, Schroeder C, et al. The accuracy of fitness watches for the measurement of heart rate and energy expenditure during moderate intensity exercise. J Sports Med Phys Fitness. 2021 Feb;61(2):205-11.

196. Jakicic JM, Marcus M, Gallagher KI, Randall C, Thomas E, Goss FL, et al. Evaluation of the SenseWear Pro Armband to assess energy expenditure during exercise. Med Sci Sports Exerc. 2004 May;36(5):897-904.

197. Johannsen DL, Calabro MA, Stewart J, Franke W, Rood JC, Welk GJ. Accuracy of armband monitors for measuring daily energy expenditure in healthy adults. Med Sci Sports Exerc. 2010 Nov;42(11):2134-40.

198. Jubeen F, Jabeen I, Aftab U, Noor S, Hareem ME, Sultan M, et al. Synthesis, Characterization, Theoretical and Experimental Anticancer Evaluation of Novel Cocrystals of 5-Fluorouracil and Schiff Bases against SW480 Colorectal Carcinoma. Pharmaceutics. 2023 Jul 11;15(7).

199. Kaewkannate K, Kim S. A comparison of wearable fitness devices. BMC Public Health. 2016 May 24;16:433.

200. Kang SG, Kang JM, Ko KP, Park SC, Mariani S, Weng J. Validity of a commercial wearable sleep tracker in adult insomnia disorder patients and good sleepers. J Psychosom Res. 2017 Jun;97:38-44.

201. Karinharju KS, Boughey AM, Tweedy SM, Clanchy KM, Trost SG, Gomersall SR. Validity of the Apple Watch® for monitoring push counts in people using manual wheelchairs. The Journal of Spinal Cord Medicine. 2021 2021/03/04;44(2):212-20.

202. Kearley K, Selwood M, Van den Bruel A, Thompson M, Mant D, Hobbs FR, et al. Triage tests for identifying atrial fibrillation in primary care: a diagnostic accuracy study comparing single-lead ECG and modified BP monitors. BMJ Open. 2014 May 2;4(5):e004565.

203. Kendall B, Bellovary B, Gothe NP. Validity of wearable activity monitors for tracking steps and estimating energy expenditure during a graded maximal treadmill test. J Sports Sci. 2019 Jan;37(1):42-9.

204. Khan R, Naseem I. Antiglycation and antioxidant potential of coumaric acid isomers: a comparative in-vitro study. J Biomol Struct Dyn. 2023 Oct 18:1-15.

205. Khushhal A, Nichols S, Evans W, Gleadall-Siddall DO, Page R, O'Doherty AF, et al. Validity and Reliability of the Apple Watch for Measuring Heart Rate During Exercise. Sports Med Int Open. 2017 Oct;1(6):E206-e11.

206. Kim Y, Welk GJ. Criterion Validity of Competing Accelerometry-Based Activity Monitoring Devices. Med Sci Sports Exerc. 2015 Nov;47(11):2456-63.

207. Kimura T, Aizawa Y, Kurata N, Nakajima K, Kashimura S, Kunitomi A, et al. Assessment of atrial fibrillation ablation outcomes with clinic ECG, monthly 24-h Holter ECG, and twice-daily telemonitoring ECG. Heart Vessels. 2017 Mar;32(3):317-25.

208. King GA, Torres N, Potter C, Brooks TJ, Coleman KJ. Comparison of activity monitors to estimate energy cost of treadmill exercise. Med Sci Sports Exerc. 2004 Jul;36(7):1244-51.

209. Kingsley M, Lewis MJ, Marson RE. Comparison of Polar 810s and an ambulatory ECG system for RR interval measurement during progressive exercise. Int J Sports Med. 2005 Jan-Feb;26(1):39-44.

210. Klepin K, Wing D, Higgins M, Nichols J, Godino JG. Validity of Cardiorespiratory Fitness Measured with Fitbit Compared to V O2max. Med Sci Sports Exerc. 2019 Nov;51(11):2251-6.

211. Koehler K, Braun H, de Marees M, Fusch G, Fusch C, Schaenzer W. Assessing energy expenditure in male endurance athletes: validity of the SenseWear Armband. Med Sci Sports Exerc. 2011 Jul;43(7):1328-33.

212. Kooiman TJ, Dontje ML, Sprenger SR, Krijnen WP, van der Schans CP, de Groot M. Reliability and validity of ten consumer activity trackers. BMC Sports Sci Med Rehabil. 2015;7:24.

213. Koshy AN, Sajeev JK, Nerlekar N, Brown AJ, Rajakariar K, Zureik M, et al. Smart watches for heart rate assessment in atrial arrhythmias. Int J Cardiol. 2018 Sep 1;266:124-7.

214. Kraft GL, Dow M. Validation of the Polar Fitness Test. International Journal for Innovation Education and Research. 2018 01/01;6(1):27-34.

215. Kraft GL, Roberts RA. Validation of the Garmin Forerunner 920XT Fitness Watch VO2peak Test. International Journal for Innovation Education and Research. 2017 02/01;5(2):63-9.

216. Kressler J, Koeplin-Day J, Muendle B, Rosby B, Santo E, Domingo A. Accuracy and precision of consumer-level activity monitors for stroke detection during wheelchair propulsion and arm ergometry. PLoS One. 2018;13(2):e0191556.

217. Kroll RR, Boyd JG, Maslove DM. Accuracy of a Wrist-Worn Wearable Device for Monitoring Heart Rates in Hospital Inpatients: A Prospective Observational Study. J Med Internet Res. 2016 Sep 20;18(9):e253.

218. Kubala AG, Barone Gibbs B, Buysse DJ, Patel SR, Hall MH, Kline CE. Field-based Measurement of Sleep: Agreement between Six Commercial Activity Monitors and a Validated Accelerometer. Behav Sleep Med. 2020 Sep-Oct;18(5):637-52.

219. Kwon S, Hong J, Choi EK, Lee B, Baik C, Lee E, et al. Detection of Atrial Fibrillation Using a Ring-Type Wearable Device (CardioTracker) and Deep Learning Analysis of Photoplethysmography Signals: Prospective Observational Proof-of-Concept Study. J Med Internet Res. 2020 May 21;22(5):e16443.

220. Lahdenoja O, Hurnanen T, Iftikhar Z, Nieminen S, Knuutila T, Saraste A, et al. Atrial Fibrillation Detection via Accelerometer and Gyroscope of a Smartphone. IEEE J Biomed Health Inform. 2018 Jan;22(1):108-18.

221. Lai B, Sasaki JE, Jeng B, Cederberg KL, Bamman MM, Motl RW. Accuracy and Precision of Three Consumer-Grade Motion Sensors During Overground and Treadmill Walking in People With Parkinson Disease: Cross-Sectional Comparative Study. JMIR Rehabil Assist Technol. 2020 Jan 16;7(1):e14059.

222. Lamont RM, Daniel HL, Payne CL, Brauer SG. Accuracy of wearable physical activity trackers in people with Parkinson's disease. Gait Posture. 2018 Jun;63:104-8.

223. Lau JK, Lowres N, Neubeck L, Brieger DB, Sy RW, Galloway CD, et al. iPhone ECG application for community screening to detect silent atrial fibrillation: a novel technology to prevent stroke. Int J Cardiol. 2013 Apr 30;165(1):193-4.

224. Lawinger E, Uhl TL, Abel M, Kamineni S. Assessment of Accelerometers for Measuring Upper-Extremity Physical Activity. J Sport Rehabil. 2015 Aug;24(3):236-43.

225. Lee CM, Gorelick M, Mendoza A. Accuracy of an infrared LED device to measure heart rate and energy expenditure during rest and exercise. J Sports Sci. 2011 Dec;29(15):1645-53.

226. Lee HA, Lee HJ, Moon JH, Lee T, Kim MG, In H, et al. Comparison of Wearable Activity Tracker with Actigraphy for Sleep Evaluation and Circadian Rest-Activity Rhythm Measurement in Healthy Young Adults. Psychiatry Investig. 2017 Mar;14(2):179-85.

227. Lee IM, Shiroma EJ. Using accelerometers to measure physical activity in large-scale epidemiological studies: issues and challenges. Br J Sports Med. 2014 Feb;48(3):197-201.

228. Lee J, Reyes BA, McManus DD, Mathias O, Chon KH. Atrial fibrillation detection using a smart phone. Annu Int Conf IEEE Eng Med Biol Soc. 2012;2012:1177-80.

229. Lee JM, Byun W, Keill A, Dinkel D, Seo Y. Comparison of Wearable Trackers' Ability to Estimate Sleep. Int J Environ Res Public Health. 2018 Jun 15;15(6).

230. Lee JM, Kim Y, Welk GJ. Validity of consumer-based physical activity monitors. Med Sci Sports Exerc. 2014 Sep;46(9):1840-8.

231. Lee M, Park S, Lee H. Accuracy of swimming wearable watches for estimating energy expenditure. IJASS(International Journal of Applied Sports Sciences). 2018 06/01;30:80-90.

232. Lemmens PMC, Sartor F, Cox LGE, den Boer SV, Westerink J. Evaluation of an activity monitor for use in pregnancy to help reduce excessive gestational weight gain. BMC Pregnancy Childbirth. 2018 Jul 31;18(1):312.

233. Leth S, Hansen J, Nielsen OW, Dinesen B. Evaluation of Commercial Self-Monitoring Devices for Clinical Purposes: Results from the Future Patient Trial, Phase I. Sensors (Basel). 2017 Jan 22;17(1).

234. Leving MT, Horemans HLD, Vegter RJK, de Groot S, Bussmann JBJ, van der Woude LHV. Validity of consumer-grade activity monitor to identify manual wheelchair propulsion in standardized activities of daily living. PLoS One. 2018;13(4):e0194864.

235. Li S, Tong J, Li H, Mao C, Shen W, Lei Y, et al. L. pneumophila Infection Diagnosed by tNGS in a Lady with Lymphadenopathy. Infect Drug Resist. 2023;16:4435-42.

236. Liang Z, Chapa Martell MA. Validity of Consumer Activity Wristbands and Wearable EEG for Measuring Overall Sleep Parameters and Sleep Structure in Free-Living Conditions. J Healthc Inform Res. 2018 Jun;2(1-2):152-78.

237. Lin CT, Chang KC, Lin CL, Chiang CC, Lu SW, Chang SS, et al. An intelligent telecardiology system using a wearable and wireless ECG to detect atrial fibrillation. IEEE Trans Inf Technol Biomed. 2010 May;14(3):726-33.

238. Littell L, Roelle L, Dalal A, Van Hare GF, Orr WB, Miller N, et al. Assessment of Apple Watch Series 6 pulse oximetry and electrocardiograms in a pediatric population. PLOS Digit Health. 2022 Aug;1(8):e0000051.

239. Liu J, Wong WT, Zwetsloot IM, Hsu YC, Tsui KL. Preliminary Agreement on Tracking Sleep Between a Wrist-Worn Device Fitbit Alta and Consensus Sleep Diary. Telemed J E Health. 2019 Dec;25(12):1189-97.

240. Lopez GA, Brond JC, Andersen LB, Dencker M, Arvidsson D. Validation of SenseWear Armband in children, adolescents, and adults. Scand J Med Sci Sports. 2018 Feb;28(2):487-95.

241. Lown M, Yue AM, Shah BN, Corbett SJ, Lewith G, Stuart B, et al. Screening for Atrial Fibrillation Using Economical and Accurate Technology (From the SAFETY Study). Am J Cardiol. 2018 Oct 15;122(8):1339-44.

242. Mackey DC, Manini TM, Schoeller DA, Koster A, Glynn NW, Goodpaster BH, et al. Validation of an armband to measure daily energy expenditure in older adults. J Gerontol A Biol Sci Med Sci. 2011 Oct;66(10):1108-13.

243. Macko RF, Haeuber E, Shaughnessy M, Coleman KL, Boone DA, Smith GV, et al. Microprocessor-based ambulatory activity monitoring in stroke patients. Med Sci Sports Exerc. 2002 Mar;34(3):394-9.

244. Madigan EA. Fitness band accuracy in older community dwelling adults. Health Informatics J. 2019 Sep;25(3):676-82.

245. Magistro D, Brustio PR, Ivaldi M, Esliger DW, Zecca M, Rainoldi A, et al. Validation of the ADAMO Care Watch for step counting in older adults. PLoS One. 2018;13(2):e0190753.

246. Mammen G, Gardiner S, Senthinathan A, McClemont L, Stone M, Faulkner G. Is this Bit Fit? Measuring the Quality of the Fitbit Step-Counter. . **The Health & Fitness Journal of Canada**. 2012;5(4):30-9.

247. Mandigout S, Lacroix J, Ferry B, Vuillerme N, Compagnat M, Daviet JC. Can energy expenditure be accurately assessed using accelerometry-based wearable motion detectors for physical activity monitoring in post-stroke patients in the subacute phase? Eur J Prev Cardiol. 2017 Dec;24(18):2009-16.

248. Manns P, Orchard J, Warren S. Accuracy of Pedometry for Ambulatory Adults with Neurological Disabilities. Physiotherpy Canada. 2007.

249. Mantua J, Gravel N, Spencer RM. Reliability of Sleep Measures from Four Personal Health Monitoring Devices Compared to Research-Based Actigraphy and Polysomnography. Sensors (Basel). 2016 May 5;16(5).

250. Marazzi G, Iellamo F, Volterrani M, Lombardo M, Pelliccia F, Righi D, et al. Comparison of Microlife BP A200 Plus and Omron M6 blood pressure monitors to detect atrial fibrillation in hypertensive patients. Adv Ther. 2012 Jan;29(1):64-70.

251. Marques HO, Swersky L, Sander J, Campello R, Zimek A. On the evaluation of outlier detection and one-class classification: a comparative study of algorithms, model selection, and ensembles. Data Min Knowl Discov. 2023;37(4):1473-517.

252. Martien S, Seghers J, Boen F, Delecluse C. Energy Expenditure in Institutionalized Older Adults: Validation of SenseWear Mini. Med Sci Sports Exerc. 2015 Jun;47(6):1265-71.

253. Maskevich S, Jumabhoy R, Dao PDM, Stout JC, Drummond SPA. Pilot Validation of Ambulatory Activity Monitors for Sleep Measurement in Huntington's Disease Gene Carriers. J Huntingtons Dis. 2017;6(3):249-53.

254. Mc MD, Chong JW, Soni A, Saczynski JS, Esa N, Napolitano C, et al. PULSE-SMART: Pulse-Based Arrhythmia Discrimination Using a Novel Smartphone Application. J Cardiovasc Electrophysiol. 2016 Jan;27(1):51-7.

255. McManus DD, Lee J, Maitas O, Esa N, Pidikiti R, Carlucci A, et al. A novel application for the detection of an irregular pulse using an iPhone 4S in patients with atrial fibrillation. Heart Rhythm. 2013 Mar;10(3):315-9.

256. McMinn D, Rowe DA, Murtagh S, Nelson NM. The effect of a school-based active commuting intervention on children's commuting physical activity and daily physical activity. Prev Med. 2012 May;54(5):316-8.

257. Melanson EL, Dykstra JC, Szuminsky N. A novel approach for measuring energy expenditure in free-living humans. Annu Int Conf IEEE Eng Med Biol Soc. 2009;2009:6873-7.

258. Meltzer LJ, Hiruma LS, Avis K, Montgomery-Downs H, Valentin J. Comparison of a Commercial Accelerometer with Polysomnography and Actigraphy in Children and Adolescents. Sleep. 2015 Aug 1;38(8):1323-30.

259. Menghini L, Gianfranchi E, Cellini N, Patron E, Tagliabue M, Sarlo M. Stressing the accuracy: Wrist-worn wearable sensor validation over different conditions. Psychophysiology. 2019 Nov;56(11):e13441.

260. Miller DJ, Lastella M, Scanlan AT, Bellenger C, Halson SL, Roach GD, et al. A validation study of the WHOOP strap against polysomnography to assess sleep. Journal of Sports Sciences. 2020 2020/11/16;38(22):2631-6.

261. Miller DJ, Roach GD, Lastella M, Scanlan AT, Bellenger CR, Halson SL, et al. A Validation Study of a Commercial Wearable Device to Automatically Detect and Estimate Sleep. Biosensors (Basel). 2021 Jun 8;11(6).

262. Miller DJ, Sargent C, Roach GD. A Validation of Six Wearable Devices for Estimating Sleep, Heart Rate and Heart Rate Variability in Healthy Adults. Sensors (Basel). 2022 Aug 22;22(16).

263. Modave F, Guo Y, Bian J, Gurka MJ, Parish A, Smith MD, et al. Mobile Device Accuracy for Step Counting Across Age Groups. JMIR Mhealth Uhealth. 2017 Jun 28;5(6):e88.

264. Montano A, Brown F, Credeur DP, Williams MA, Stoner L. Telemetry-derived heart rate variability responses to a physical stressor. Clin Physiol Funct Imaging. 2017 Jul;37(4):421-7.

265. Montes J, Young JC, Tandy RD, Navalta JW. Fitbit Flex Energy Expenditure and Step Count Evaluation. Journal of exercise physiology. 2017;20:134.

266. Montgomery-Downs HE, Insana SP, Bond JA. Movement toward a novel activity monitoring device. Sleep Breath. 2012 Sep;16(3):913-7.

267. Morris CE, Wessel PA, Tinius RA, Schafer MA, Maples JM. Validity of Activity Trackers in Estimating Energy Expenditure During High-Intensity Functional Training. Res Q Exerc Sport. 2019 Sep;90(3):377-84.

268. Motl RW, McAuley E, Snook EM, Scott JA. Accuracy of two electronic pedometers for measuring steps taken under controlled conditions among ambulatory individuals with multiple sclerosis. Mult Scler. 2005 Jun;11(3):343-5.

269. Mouritzen NJ, Larsen LH, Lauritzen MH, Kjær TW. Assessing the performance of a commercial multisensory sleep tracker. PLoS One. 2020;15(12):e0243214.

270. Muggeridge DJ, Hickson K, Davies AV, Giggins OM, Megson IL, Gorely T, et al. Measurement of Heart Rate Using the Polar OH1 and Fitbit Charge 3 Wearable Devices in Healthy Adults During Light, Moderate, Vigorous, and Sprint-Based Exercise: Validation Study. JMIR Mhealth Uhealth. 2021 Mar 25;9(3):e25313.

271. Muller AM, Wang NX, Yao J, Tan CS, Low ICC, Lim N, et al. Heart Rate Measures From Wrist-Worn Activity Trackers in a Laboratory and Free-Living Setting: Validation Study. JMIR Mhealth Uhealth. 2019 Oct 2;7(10):e14120.

272. Murakami H, Kawakami R, Nakae S, Nakata Y, Ishikawa-Takata K, Tanaka S, et al. Accuracy of Wearable Devices for Estimating Total Energy Expenditure: Comparison With Metabolic Chamber and Doubly Labeled Water Method. JAMA Intern Med. 2016 May 1;176(5):702-3.

273. Naslund JA, Aschbrenner KA, Barre LK, Bartels SJ. Feasibility of popular m-health technologies for activity tracking among individuals with serious mental illness. Telemed J E Health. 2015 Mar;21(3):213-6.

274. Nazari G, MacDermid JC, Sinden KE, Richardson J, Tang A. Inter-Instrument Reliability and Agreement of Fitbit Charge Measurements of Heart Rate and Activity at Rest, during the Modified Canadian Aerobic Fitness Test, and in Recovery. Physiother Can. 2019 Summer;71(3):197-206.

275. Nelson BW, Allen NB. Accuracy of Consumer Wearable Heart Rate Measurement During an Ecologically Valid 24-Hour Period: Intraindividual Validation Study. JMIR Mhealth Uhealth. 2019 Mar 11;7(3):e10828.

276. Nelson MB, Kaminsky LA, Dickin DC, Montoye AH. Validity of Consumer-Based Physical Activity Monitors for Specific Activity Types. Med Sci Sports Exerc. 2016 Aug;48(8):1619-28.

277. Nemati S, Ghassemi MM, Ambai V, Isakadze N, Levantsevych O, Shah A, et al. Monitoring and detecting atrial fibrillation using wearable technology. Annu Int Conf IEEE Eng Med Biol Soc. 2016 Aug;2016:3394-7.

278. Nunan D, Donovan G, Jakovljevic DG, Hodges LD, Sandercock GR, Brodie DA. Validity and reliability of short-term heart-rate variability from the Polar S810. Med Sci Sports Exerc. 2009 Jan;41(1):243-50.

279. Nunan D, Jakovljevic DG, Donovan G, Hodges LD, Sandercock GR, Brodie DA. Levels of agreement for RR intervals and short-term heart rate variability obtained from the Polar S810 and an alternative system. Eur J Appl Physiol. 2008 Jul;103(5):529-37.

280. Nuss KJ, Thomson EA, Courtney JB, Comstock A, Reinwald S, Blake S, et al. Assessment of Accuracy of Overall Energy Expenditure Measurements for the Fitbit Charge HR 2 and Apple Watch. Am J Health Behav. 2019 May 1;43(3):498-505.

281. O'Connell S, G OL, Kelly L, Murphy E, Beirne S, Burke N, et al. These Shoes Are Made for Walking: Sensitivity Performance Evaluation of Commercial Activity Monitors under the Expected Conditions and Circumstances Required to Achieve the International Daily Step Goal of 10,000 Steps. PLoS One. 2016;11(5):e0154956.

282. O'Connell S, G OL, Quinlan LR. When a Step Is Not a Step! Specificity Analysis of Five Physical Activity Monitors. PLoS One. 2017;12(1):e0169616.

283. Omboni S, Verberk WJ. Opportunistic screening of atrial fibrillation by automatic blood pressure measurement in the community. BMJ Open. 2016 Apr 12;6(4):e010745.

284. Osterbauer B, Koempel J, Ward S, Fisher L, Don D. A Comparison Study Of The Fitbit Activity Monitor And PSG For Assessing Sleep Patterns And Movement In Children. Journal of Otolaryngology Advances. 2016 02/01;1:24.

285. Papazoglou D, Augello G, Tagliaferri M, Savia G, Marzullo P, Maltezos E, et al. Evaluation of a multisensor armband in estimating energy expenditure in obese individuals. Obesity (Silver Spring). 2006 Dec;14(12):2217-23.

286. Pasadyn SR, Soudan M, Gillinov M, Houghtaling P, Phelan D, Gillinov N, et al. Accuracy of commercially available heart rate monitors in athletes: a prospective study. Cardiovasc Diagn Ther. 2019 Aug;9(4):379-85.

287. Passler S, Bohrer J, Blochinger L, Senner V. Validity of Wrist-Worn Activity Trackers for Estimating VO(2max) and Energy Expenditure. Int J Environ Res Public Health. 2019 Aug 22;16(17).

288. Patz C, Michaelis A, Markel F, Loffelbein F, Dahnert I, Gebauer RA, et al. Accuracy of the Apple Watch Oxygen Saturation Measurement in Adults and Children with Congenital Heart Disease. Pediatr Cardiol. 2023 Feb;44(2):333-43.

289. Pelizzo G, Guddo A, Puglisi A, De Silvestri A, Comparato C, Valenza M, et al. Accuracy of a Wrist-Worn Heart Rate Sensing Device during Elective Pediatric Surgical Procedures. Children (Basel). 2018 Mar 8;5(3).

290. Perez MV, Mahaffey KW, Hedlin H, Rumsfeld JS, Garcia A, Ferris T, et al. Large-Scale Assessment of a Smartwatch to Identify Atrial Fibrillation. N Engl J Med. 2019 Nov 14;381(20):1909-17.

291. Phillips LJ, Petroski GF, Markis NE. A Comparison of Accelerometer Accuracy in Older Adults. Res Gerontol Nurs. 2015 Sep-Oct;8(5):213-9.

292. Pike K, Moller CI, Bryant C, Farrow M, Dao DP, Ellis KA. Examination of the Feasibility, Acceptability, and Efficacy of the Online Personalised Training in Memory Strategies for Everyday Program for Older Adults: Single-Arm Pre-Post Trial. J Med Internet Res. 2023 Apr 20;25:e41712.

293. Pipek LZ, Nascimento RFV, Acencio MMP, Teixeira LR. Comparison of SpO(2) and heart rate values on Apple Watch and conventional commercial oximeters devices in patients with lung disease. Sci Rep. 2021 Sep 23;11(1):18901.

294. Pitrez PM, Nanthapisal S, Castro A, Teli C, P GA. Managing moderate-to-severe paediatric asthma: a scoping review of the efficacy and safety of fluticasone propionate/salmeterol. BMJ Open Respir Res. 2023 Aug;10(1).

295. Plews DJ, Scott B, Altini M, Wood M, Kilding AE, Laursen PB. Comparison of Heart-Rate-Variability Recording With Smartphone Photoplethysmography, Polar H7 Chest Strap, and Electrocardiography. Int J Sports Physiol Perform. 2017 Nov 1;12(10):1324-8.

296. Poh MZ, Poh YC, Chan PH, Wong CK, Pun L, Leung WW, et al. Diagnostic assessment of a deep learning system for detecting atrial fibrillation in pulse waveforms. Heart. 2018 Dec;104(23):1921-8.

297. Poojary J, Arora E, Britto A, Polen Z, Arena R, Babu AS. Validity of Mobile-Based Technology vs Direct Observation in Measuring Number of Steps and Distance Walked in 6 Minutes. Mayo Clin Proc. 2018 Dec;93(12):1873-4.

298. Pope ZC, Lee JE, Zeng N, Gao Z. Validation of Four Smartwatches in Energy Expenditure and Heart Rate Assessment During Exergaming. Games Health J. 2019 Jun;8(3):205-12.

299. Porto LG, Junqueira LF, Jr. Comparison of time-domain short-term heart interval variability analysis using a wrist-worn heart rate monitor and the conventional electrocardiogram. Pacing Clin Electrophysiol. 2009 Jan;32(1):43-51.

300. Powierza CS, Clark MD, Hughes JM, Carneiro KA, Mihalik JP. Validation of a Self-Monitoring Tool for Use in Exercise Therapy. Pm r. 2017 Nov;9(11):1077-84.

301. Pribyslavska V, Caputo JL, Coons JM, Barry VW. Impact of EPOC adjustment on estimation of energy expenditure using activity monitors. J Med Eng Technol. 2018 May;42(4):265-73.

302. Price K, Bird SR, Lythgo N, Raj IS, Wong JY, Lynch C. Validation of the Fitbit One, Garmin Vivofit and Jawbone UP activity tracker in estimation of energy expenditure during treadmill walking and running. J Med Eng Technol. 2017 Apr;41(3):208-15.

303. Prieto-Centurion V, Bracken N, Norwick L, Zaidi F, Mutso AA, Morken V, et al. Can Commercially Available Pedometers Be Used For Physical Activity Monitoring In Patients With COPD Following Exacerbations? Chronic Obstr Pulm Dis. 2016;3(3):636-42.

304. Proesmans T, Mortelmans C, Van Haelst R, Verbrugge F, Vandervoort P, Vaes B. Mobile Phone-Based Use of the Photoplethysmography Technique to Detect Atrial Fibrillation in Primary Care: Diagnostic Accuracy Study of the FibriCheck App. JMIR Mhealth Uhealth. 2019 Mar 27;7(3):e12284.

305. Quer G, Nikzad N, Lanka S, van Mourik R, Dur O, SR. S. Preliminary Evaluation of a Wrist Wearable Heart Rate Sensor for the Detection of Undiagnosed Atrial Fibrillation in a Real-World Setting. Circulation. 2017.

306. Rafl J, Bachman TE, Rafl-Huttova V, Walzel S, Rozanek M. Commercial smartwatch with pulse oximeter detects short-time hypoxemia as well as standard medical-grade device: Validation study. Digit Health. 2022 Jan-Dec;8:20552076221132127.

307. Rajakariar K, Koshy AN, Sajeev JK, Nair S, Roberts L, Teh AW. Accuracy of a smartwatch based single-lead electrocardiogram device in detection of atrial fibrillation. Heart. 2020 May;106(9):665-70.

308. Reddy RK, Pooni R, Zaharieva DP, Senf B, El Youssef J, Dassau E, et al. Accuracy of Wrist-Worn Activity Monitors During Common Daily Physical Activities and Types of Structured Exercise: Evaluation Study. JMIR Mhealth Uhealth. 2018 Dec 10;6(12):e10338.

309. Redenius N, Kim Y, Byun W. Concurrent validity of the Fitbit for assessing sedentary behavior and moderate-to-vigorous physical activity. BMC Med Res Methodol. 2019 Feb 7;19(1):29.

310. Reece JD, Barry V, Fuller DK, Caputo J. Validation of the SenseWear Armband as a Measure of Sedentary Behavior and Light Activity. J Phys Act Health. 2015 Sep;12(9):1229-37.

311. Reeve MD, Pumpa KL, Ball N. Accuracy of the SenseWear Armband Mini and the BodyMedia FIT in resistance training. J Sci Med Sport. 2014 Nov;17(6):630-4.

312. Reid RER, Insogna JA, Carver TE, Comptour AM, Bewski NA, Sciortino C, et al. Validity and reliability of Fitbit activity monitors compared to ActiGraph GT3X+ with female adults in a free-living environment. J Sci Med Sport. 2017 Jun;20(6):578-82.

313. Renerts K, Doheny EP, Baumann C, Werth E, Baumgartner P, Busse M, et al. F63 Validation of fitbit charge 4 for sleep monitoring in participants with Huntington’s disease. Journal of Neurology, Neurosurgery &amp; Psychiatry. 2022;93(Suppl 1):A59-A.

314. Reverberi C, Rabia G, De Rosa F, Bosi D, Botti A, Benatti G. The RITMIA Smartphone App for Automated Detection of Atrial Fibrillation: Accuracy in Consecutive Patients Undergoing Elective Electrical Cardioversion. Biomed Res Int. 2019;2019:4861951.

315. Romagnoli M, Alis R, Guillen J, Basterra J, Villacastin JP, Guillen S. A novel device based on smart textile to control heart's activity during exercise. Australas Phys Eng Sci Med. 2014 Jun;37(2):377-84.

316. Roos L, Taube W, Beeler N, Wyss T. Validity of sports watches when estimating energy expenditure during running. BMC Sports Sci Med Rehabil. 2017;9:22.

317. Rosenberger ME, Buman MP, Haskell WL, McConnell MV, Carstensen LL. Twenty-four Hours of Sleep, Sedentary Behavior, and Physical Activity with Nine Wearable Devices. Med Sci Sports Exerc. 2016 Mar;48(3):457-65.

318. Rousset S, Fardet A, Lacomme P, Normand S, Montaurier C, Boirie Y, et al. Comparison of total energy expenditure assessed by two devices in controlled and free-living conditions. Eur J Sport Sci. 2015;15(5):391-9.

319. Rowlands AV, Harrington DM, Bodicoat DH, Davies MJ, Sherar LB, Gorely T, et al. Compliance of Adolescent Girls to Repeated Deployments of Wrist-Worn Accelerometers. Med Sci Sports Exerc. 2018 Jul;50(7):1508-17.

320. Rozanski GM, Aqui A, Sivakumaran S, Mansfield A. Consumer Wearable Devices for Activity Monitoring Among Individuals After a Stroke: A Prospective Comparison. JMIR Cardio. 2018 Jan 4;2(1):e1.

321. Rozen G, Vaid J, Hosseini SM, Kaadan MI, Rafael A, Roka A, et al. Diagnostic Accuracy of a Novel Mobile Phone Application for the Detection and Monitoring of Atrial Fibrillation. Am J Cardiol. 2018 May 15;121(10):1187-91.

322. Sabar MI, Ara F, Henderson A, Ahmed O, Potter C, John I, et al. A study to assess a novel automated electrocardiogram technology in screening for atrial fibrillation. Pacing Clin Electrophysiol. 2019 Oct;42(10):1383-9.

323. Sala DA, Grissom HE, Delsole EM, Chu ML, Godfried DH, Bhattacharyya S, et al. Measuring ambulation with wrist-based and hip-based activity trackers for children with cerebral palsy. Dev Med Child Neurol. 2019 Nov;61(11):1309-13.

324. Samadi M, Momtazi S. Fake news detection: deep semantic representation with enhanced feature engineering. Int J Data Sci Anal. 2023 Mar 9:1-12.

325. Sanudo B, De Hoyo M, Munoz-Lopez A, Perry J, Abt G. Pilot Study Assessing the Influence of Skin Type on the Heart Rate Measurements Obtained by Photoplethysmography with the Apple Watch. J Med Syst. 2019 May 22;43(7):195.

326. Sargent C, Lastella M, Romyn G, Versey N, Miller DJ, Roach GD. How well does a commercially available wearable device measure sleep in young athletes? Chronobiol Int. 2018 Jun;35(6):754-8.

327. Sartor F, Gelissen J, van Dinther R, Roovers D, Papini GB, Coppola G. Wrist-worn optical and chest strap heart rate comparison in a heterogeneous sample of healthy individuals and in coronary artery disease patients. BMC Sports Sci Med Rehabil. 2018;10:10.

328. Sasaki JE, Hickey A, Mavilia M, Tedesco J, John D, Kozey Keadle S, et al. Validation of the Fitbit wireless activity tracker for prediction of energy expenditure. J Phys Act Health. 2015 Feb;12(2):149-54.

329. Scalvini S, Piepoli M, Zanelli E, Volterrani M, Giordano A, Glisenti F. Incidence of atrial fibrillation in an Italian population followed by their GPs through a telecardiology service. Int J Cardiol. 2005 Feb 15;98(2):215-20.

330. Schack T, Safi Harb Y, Muma M, Zoubir AM. Computationally efficient algorithm for photoplethysmography-based atrial fibrillation detection using smartphones. Annu Int Conf IEEE Eng Med Biol Soc. 2017 Jul;2017:104-8.

331. Schaffer SD, Holzapfel SD, Fulk G, Bosch PR. Step count accuracy and reliability of two activity tracking devices in people after stroke. Physiother Theory Pract. 2017 Oct;33(10):788-96.

332. Schmal H, Holsgaard-Larsen A, Izadpanah K, Brond JC, Madsen CF, Lauritsen J. Validation of Activity Tracking Procedures in Elderly Patients after Operative Treatment of Proximal Femur Fractures. Rehabil Res Pract. 2018;2018:3521271.

333. Scott JJ, Rowlands AV, Cliff DP, Morgan PJ, Plotnikoff RC, Lubans DR. Comparability and feasibility of wrist- and hip-worn accelerometers in free-living adolescents. J Sci Med Sport. 2017 Dec;20(12):1101-6.

334. Sears T, Avalos E, Lawson S, McAlister I, ‡ C, Bunn J. Wrist-worn Physical Activity Trackers Tend to Underestimate Steps During Walking. 2017 08/01;10:764-73.

335. Selder JL, Proesmans T, Breukel L, Dur O, Gielen W, van Rossum AC, et al. Assessment of a standalone photoplethysmography (PPG) algorithm for detection of atrial fibrillation on wristband-derived data. Comput Methods Programs Biomed. 2020 Dec;197:105753.

336. Semanik P, Lee J, Pellegrini CA, Song J, Dunlop DD, Chang RW. Comparison of Physical Activity Measures Derived From the Fitbit Flex and the ActiGraph GT3X+ in an Employee Population With Chronic Knee Symptoms. ACR Open Rheumatol. 2020 Jan;2(1):48-52.

337. Seo K, Yamamoto Y, Kirillova A, Kawana M, Yadav S, Huang Y, et al. Improved Cardiac Performance and Decreased Arrhythmia in Hypertrophic Cardiomyopathy With Non-beta-Blocking R-Enantiomer Carvedilol. Circulation. 2023 Oct 18.

338. Seshadri DR, Bittel B, Browsky D, Houghtaling P, Drummond CK, Desai M, et al. Accuracy of the Apple Watch 4 to Measure Heart Rate in Patients With Atrial Fibrillation. IEEE J Transl Eng Health Med. 2020;8:2700204.

339. Seshadri DR, Bittel B, Browsky D, Houghtaling P, Drummond CK, Desai MY, et al. Accuracy of Apple Watch for Detection of Atrial Fibrillation. Circulation. 2020 Feb 25;141(8):702-3.

340. Shcherbina A, Mattsson CM, Waggott D, Salisbury H, Christle JW, Hastie T, et al. Accuracy in Wrist-Worn, Sensor-Based Measurements of Heart Rate and Energy Expenditure in a Diverse Cohort. J Pers Med. 2017 May 24;7(2).

341. Siddall AG, Powell SD, Needham-Beck SC, Edwards VC, Thompson JES, Kefyalew SS, et al. Validity of energy expenditure estimation methods during 10 days of military training. Scand J Med Sci Sports. 2019 Sep;29(9):1313-21.

342. Simpson LA, Eng JJ, Klassen TD, Lim SB, Louie DR, Parappilly B, et al. Capturing step counts at slow walking speeds in older adults: comparison of ankle and waist placement of measuring device. J Rehabil Med. 2015 Oct 5;47(9):830-5.

343. Simunek A, Dygryn J, Jakubec L, Neuls F, Fromel K, Welk GJ. Validity of Garmin Vivofit 1 and Garmin Vivofit 3 for School-Based Physical Activity Monitoring. Pediatr Exerc Sci. 2019 Feb 1;31(1):130-6.

344. Sirard JR, Masteller B, Freedson PS, Mendoza A, Hickey A. Youth Oriented Activity Trackers: Comprehensive Laboratory- and Field-Based Validation. J Med Internet Res. 2017 Jul 19;19(7):e250.

345. Sjoberg V, Westergren J, Monnier A, Lo Martire R, Hagstromer M, Ang BO, et al. Wrist-Worn Activity Trackers in Laboratory and Free-Living Settings for Patients With Chronic Pain: Criterion Validity Study. JMIR Mhealth Uhealth. 2021 Jan 12;9(1):e24806.

346. Slinde F, Bertz F, Winkvist A, Ellegard L, Olausson H, Brekke HK. Energy expenditure by multisensor armband in overweight and obese lactating women validated by doubly labeled water. Obesity (Silver Spring). 2013 Nov;21(11):2231-5.

347. Smith JD, Guerra G, Burkholder BG. The validity and accuracy of wrist-worn activity monitors in lower-limb prosthesis users. Disabil Rehabil. 2020 Nov;42(22):3182-8.

348. Smith KM, Lanningham-Foster LM, Welk GJ, Campbell CG. Validity of the SenseWear(R) Armband to predict energy expenditure in pregnant women. Med Sci Sports Exerc. 2012 Oct;44(10):2001-8.

349. Snyder NC, Willoughby CA, Smith BK. Comparison of the Polar V800 and the Garmin Forerunner 230 to Predict V̇o2max. J Strength Cond Res. 2021 May 1;35(5):1403-9.

350. Spaccarotella C, Polimeni A, Mancuso C, Pelaia G, Esposito G, Indolfi C. Assessment of Non-Invasive Measurements of Oxygen Saturation and Heart Rate with an Apple Smartwatch: Comparison with a Standard Pulse Oximeter. J Clin Med. 2022 Mar 8;11(6).

351. Speier W, Dzubur E, Zide M, Shufelt C, Joung S, Van Eyk JE, et al. Evaluating utility and compliance in a patient-based eHealth study using continuous-time heart rate and activity trackers. J Am Med Inform Assoc. 2018 Oct 1;25(10):1386-91.

352. Spierer DK, Rosen Z, Litman LL, Fujii K. Validation of photoplethysmography as a method to detect heart rate during rest and exercise. J Med Eng Technol. 2015;39(5):264-71.

353. St-Laurent A, Mony MM, Mathieu ME, Ruchat SM. Validation of the Fitbit Zip and Fitbit Flex with pregnant women in free-living conditions. J Med Eng Technol. 2018 May;42(4):259-64.

354. St-Onge M, Mignault D, Allison DB, Rabasa-Lhoret R. Evaluation of a portable device to measure daily energy expenditure in free-living adults. Am J Clin Nutr. 2007 Mar;85(3):742-9.

355. Stackpool C. Accuracy of various activity trackers in estimating steps taken and energy expenditure; 2013.

356. Stahl SE, An HS, Dinkel DM, Noble JM, Lee JM. How accurate are the wrist-based heart rate monitors during walking and running activities? Are they accurate enough? BMJ Open Sport Exerc Med. 2016;2(1):e000106.

357. Stahl ST, Insana SP. Caloric expenditure assessment among older adults: criterion validity of a novel accelerometry device. J Health Psychol. 2014 Nov;19(11):1382-7.

358. Steinhubl SR, Waalen J, Edwards AM, Ariniello LM, Mehta RR, Ebner GS, et al. Effect of a Home-Based Wearable Continuous ECG Monitoring Patch on Detection of Undiagnosed Atrial Fibrillation: The mSToPS Randomized Clinical Trial. Jama. 2018 Jul 10;320(2):146-55.

359. Stergiou GS, Karpettas N, Protogerou A, Nasothimiou EG, Kyriakidis M. Diagnostic accuracy of a home blood pressure monitor to detect atrial fibrillation. J Hum Hypertens. 2009 Oct;23(10):654-8.

360. Stiles VH, Griew PJ, Rowlands AV. Use of accelerometry to classify activity beneficial to bone in premenopausal women. Med Sci Sports Exerc. 2013 Dec;45(12):2353-61.

361. Stone JD, Rentz LE, Forsey J, Ramadan J, Markwald RR, Finomore VS, et al. Evaluations of Commercial Sleep Technologies for Objective Monitoring During Routine Sleeping Conditions. Nat Sci Sleep. 2020;12:821-42.

362. Storm FA, Heller BW, Mazza C. Step detection and activity recognition accuracy of seven physical activity monitors. PLoS One. 2015;10(3):e0118723.

363. Stove MP, Haucke E, Nymann ML, Sigurdsson T, Larsen BT. Accuracy of the wearable activity tracker Garmin Forerunner 235 for the assessment of heart rate during rest and activity. J Sports Sci. 2019 Apr;37(8):895-901.

364. Stratford PW. On "Responsiveness of the Balance Evaluation Systems Test (BESTest) in people with subacute stroke." Chinsongkram B, Chaikeeree N, Saengsirisuwan V, et al. Phys Ther. doi: 10.2522/ptj.20150621. Phys Ther. 2016 Aug;96(8):1300-1.

365. Sushames A, Edwards A, Thompson F, McDermott R, Gebel K. Validity and Reliability of Fitbit Flex for Step Count, Moderate to Vigorous Physical Activity and Activity Energy Expenditure. PLoS One. 2016;11(9):e0161224.

366. Svensson T, Chung UI, Tokuno S, Nakamura M, Svensson AK. A validation study of a consumer wearable sleep tracker compared to a portable EEG system in naturalistic conditions. J Psychosom Res. 2019 Nov;126:109822.

367. Takacs J, Pollock CL, Guenther JR, Bahar M, Napier C, Hunt MA. Validation of the Fitbit One activity monitor device during treadmill walking. J Sci Med Sport. 2014 Sep;17(5):496-500.

368. Tam KM, Cheung SY. Validation of Electronic Activity Monitor Devices During Treadmill Walking. Telemed J E Health. 2018 Oct;24(10):782-9.

369. Tedesco S, Sica M, Ancillao A, Timmons S, Barton J, O'Flynn B. Validity Evaluation of the Fitbit Charge2 and the Garmin vivosmart HR+ in Free-Living Environments in an Older Adult Cohort. JMIR Mhealth Uhealth. 2019 Jun 19;7(6):e13084.

370. Tedesco S, Sica M, Ancillao A, Timmons S, Barton J, O'Flynn B. Accuracy of consumer-level and research-grade activity trackers in ambulatory settings in older adults. PLoS One. 2019;14(5):e0216891.

371. Thiebaud RS, Funk MD, Patton JC, Massey BL, Shay TE, Schmidt MG, et al. Validity of wrist-worn consumer products to measure heart rate and energy expenditure. Digit Health. 2018 Jan-Dec;4:2055207618770322.

372. Thomson EA, Nuss K, Comstock A, Reinwald S, Blake S, Pimentel RE, et al. Heart rate measures from the Apple Watch, Fitbit Charge HR 2, and electrocardiogram across different exercise intensities. J Sports Sci. 2019 Jun;37(12):1411-9.

373. Thorup CB, Andreasen JJ, Sorensen EE, Gronkjaer M, Dinesen BI, Hansen J. Accuracy of a step counter during treadmill and daily life walking by healthy adults and patients with cardiac disease. BMJ Open. 2017 Mar 31;7(3):e011742.

374. Tison GH, Sanchez JM, Ballinger B, Singh A, Olgin JE, Pletcher MJ, et al. Passive Detection of Atrial Fibrillation Using a Commercially Available Smartwatch. JAMA Cardiol. 2018 May 1;3(5):409-16.

375. Tophoj KH, Petersen MG, Saebye C, Baad-Hansen T, Wagner S. Validity and Reliability Evaluation of Four Commercial Activity Trackers' Step Counting Performance. Telemed J E Health. 2018 Sep;24(9):669-77.

376. Torfs T, Smeets CJ, Geng D, Berset T, Van der Auwera J, Vandervoort P, et al. Clinical validation of a low-power and wearable ECG patch for long term full-disclosure monitoring. J Electrocardiol. 2014 Nov-Dec;47(6):881-9.

377. Toth LP, Park S, Springer CM, Feyerabend MD, Steeves JA, Bassett DR. Video-Recorded Validation of Wearable Step Counters under Free-living Conditions. Med Sci Sports Exerc. 2018 Jun;50(6):1315-22.

378. Treacy D, Hassett L, Schurr K, Chagpar S, Paul SS, Sherrington C. Validity of Different Activity Monitors to Count Steps in an Inpatient Rehabilitation Setting. Phys Ther. 2017 May 1;97(5):581-8.

379. Tully MA, McBride C, Heron L, Hunter RF. The validation of Fibit Zip physical activity monitor as a measure of free-living physical activity. BMC Res Notes. 2014 Dec 23;7:952.

380. Turakhia MP, Ullal AJ, Hoang DD, Than CT, Miller JD, Friday KJ, et al. Feasibility of extended ambulatory electrocardiogram monitoring to identify silent atrial fibrillation in high-risk patients: the Screening Study for Undiagnosed Atrial Fibrillation (STUDY-AF). Clin Cardiol. 2015 May;38(5):285-92.

381. Turki AF, Jani MB, Ding K, Zhang R, Behbehani K. An Investigation of Heartrate Sensing Accuracy by Wrist-Worn Fitness Tracking Devices. Annu Int Conf IEEE Eng Med Biol Soc. 2019 Jul;2019:3337-40.

382. Ummels D, Beekman E, Theunissen K, Braun S, Beurskens AJ. Counting Steps in Activities of Daily Living in People With a Chronic Disease Using Nine Commercially Available Fitness Trackers: Cross-Sectional Validity Study. JMIR Mhealth Uhealth. 2018 Apr 2;6(4):e70.

383. Valiaho ES, Kuoppa P, Lipponen JA, Martikainen TJ, Jantti H, Rissanen TT, et al. Wrist band photoplethysmography in detection of individual pulses in atrial fibrillation and algorithm-based detection of atrial fibrillation. Europace. 2019 Jul 1;21(7):1031-8.

384. Van Blarigan EL, Kenfield SA, Tantum L, Cadmus-Bertram LA, Carroll PR, Chan JM. The Fitbit One Physical Activity Tracker in Men With Prostate Cancer: Validation Study. JMIR Cancer. 2017 Apr 18;3(1):e5.

385. Van Hoye K, Boen F, Lefevre J. Validation of the SenseWear Armband in different ambient temperatures. J Sports Sci. 2015;33(10):1007-18.

386. van Hoye K, Mortelmans P, Lefevre J. Validation of the SenseWear Pro3 Armband using an incremental exercise test. J Strength Cond Res. 2014 Oct;28(10):2806-14.

387. Vanderlei LC, Silva RA, Pastre CM, Azevedo FM, Godoy MF. Comparison of the Polar S810i monitor and the ECG for the analysis of heart rate variability in the time and frequency domains. Braz J Med Biol Res. 2008 Oct;41(10):854-9.

388. Vasconcellos FV, Seabra A, Cunha FA, Montenegro RA, Bouskela E, Farinatti P. Heart rate variability assessment with fingertip photoplethysmography and polar RS800cx as compared with electrocardiography in obese adolescents. Blood Press Monit. 2015 Dec;20(6):351-60.

389. Vernillo G, Savoldelli A, Pellegrini B, Schena F. Validity of the SenseWear Armband to assess energy expenditure in graded walking. J Phys Act Health. 2015 Feb;12(2):178-83.

390. Vetrovsky T, Siranec M, Marencakova J, Tufano JJ, Capek V, Bunc V, et al. Validity of six consumer-level activity monitors for measuring steps in patients with chronic heart failure. PLoS One. 2019;14(9):e0222569.

391. Wahl Y, Duking P, Droszez A, Wahl P, Mester J. Criterion-Validity of Commercially Available Physical Activity Tracker to Estimate Step Count, Covered Distance and Energy Expenditure during Sports Conditions. Front Physiol. 2017;8:725.

392. Wallen MB, Hasson D, Theorell T, Canlon B, Osika W. Possibilities and limitations of the Polar RS800 in measuring heart rate variability at rest. Eur J Appl Physiol. 2012 Mar;112(3):1153-65.

393. Wallen MP, Gomersall SR, Keating SE, Wisloff U, Coombes JS. Accuracy of Heart Rate Watches: Implications for Weight Management. PLoS One. 2016;11(5):e0154420.

394. Wang M, Pan W, Xu Y, Zhang J, Wan J, Jiang H. Microglia-Mediated Neuroinflammation: A Potential Target for the Treatment of Cardiovascular Diseases. J Inflamm Res. 2022;15:3083-94.

395. Wang R, Blackburn G, Desai M, Phelan D, Gillinov L, Houghtaling P, et al. Accuracy of Wrist-Worn Heart Rate Monitors. JAMA Cardiol. 2017 Jan 1;2(1):104-6.

396. Wasserlauf J, You C, Patel R, Valys A, Albert D, Passman R. Smartwatch Performance for the Detection and Quantification of Atrial Fibrillation. Circ Arrhythm Electrophysiol. 2019 Jun;12(6):e006834.

397. Weippert M, Kumar M, Kreuzfeld S, Arndt D, Rieger A, Stoll R. Comparison of three mobile devices for measuring R-R intervals and heart rate variability: Polar S810i, Suunto t6 and an ambulatory ECG system. Eur J Appl Physiol. 2010 Jul;109(4):779-86.

398. Wiesel J, Abraham S, Messineo FC. Screening for asymptomatic atrial fibrillation while monitoring the blood pressure at home: trial of regular versus irregular pulse for prevention of stroke (TRIPPS 2.0). Am J Cardiol. 2013 Jun 1;111(11):1598-601.

399. Wiesel J, Fitzig L, Herschman Y, Messineo FC. Detection of atrial fibrillation using a modified microlife blood pressure monitor. Am J Hypertens. 2009 Aug;22(8):848-52.

400. Wiesel J, Wiesel D, Suri R, Messineo FC. The use of a modified sphygmomanometer to detect atrial fibrillation in outpatients. Pacing Clin Electrophysiol. 2004 May;27(5):639-43.

401. William AD, Kanbour M, Callahan T, Bhargava M, Varma N, Rickard J, et al. Assessing the accuracy of an automated atrial fibrillation detection algorithm using smartphone technology: The iREAD Study. Heart Rhythm. 2018 Oct;15(10):1561-5.

402. Wineinger NE, Barrett PM, Zhang Y, Irfanullah I, Muse ED, Steinhubl SR, et al. Identification of paroxysmal atrial fibrillation subtypes in over 13,000 individuals. Heart Rhythm. 2019 Jan;16(1):26-30.

403. Wong CK, Mentis HM, Kuber R. The bit doesn't fit: Evaluation of a commercial activity-tracker at slower walking speeds. Gait Posture. 2018 Jan;59:177-81.

404. Woodman JA, Crouter SE, Bassett DR, Jr., Fitzhugh EC, Boyer WR. Accuracy of Consumer Monitors for Estimating Energy Expenditure and Activity Type. Med Sci Sports Exerc. 2017 Feb;49(2):371-7.

405. Wu CF, Yang CY, Li AH, Chuang WP, Chen KC, Liu YH, et al. Detection of asymptomatic paroxysmal atrial fibrillation with the trans-telephonic electrocardiograph system. Telemed J E Health. 2012 Apr;18(3):193-7.

406. Xia T, Picco L, Lalic S, Buchbinder R, Bell JS, Andrew NE, et al. Determining the Impact of Opioid Policy on Substance Use and Mental Health-Related Harms: Protocol for a Data Linkage Study. JMIR Res Protoc. 2023 Oct 17;12:e51825.

407. Xie J, Wen D, Liang L, Jia Y, Gao L, Lei J. Evaluating the Validity of Current Mainstream Wearable Devices in Fitness Tracking Under Various Physical Activities: Comparative Study. JMIR Mhealth Uhealth. 2018 Apr 12;6(4):e94.

408. Xie M, Xu Y, Song L, Wang J, Lv X, Zhang Y. Corrigendum to Tissue-Engineered Buccal Mucosa Using Silk Fibroin Matrices for Urethral Reconstruction in a Canine Model, J. Surg. Res. 2014 May 1; 188 (1): 1-7. Doi: 10.1016/j.jss.2013.11.1102. J Surg Res. 2023 Oct 11;293:578-9.

409. Yan BP, Lai WHS, Chan CKY, Chan SC, Chan LH, Lam KM, et al. Contact-Free Screening of Atrial Fibrillation by a Smartphone Using Facial Pulsatile Photoplethysmographic Signals. J Am Heart Assoc. 2018 Apr 5;7(8).

410. Yin S, Chang Y, Yan X, Feng X, Wu N. Effect of acupuncture for patients with knee osteoarthritis: study protocol for a double-dummy randomized controlled trial. J Orthop Surg Res. 2023 Oct 17;18(1):779.

411. Yuan ZM, Li M, Ji CY, Li L, Jia L, Incecik A. Steady hydrodynamic interaction between human swimmers. J R Soc Interface. 2019 Jan 31;16(150):20180768.

412. Zhang H, Zhang J, Li HB, Chen YX, Yang B, Guo YT, et al. Validation of Single Centre Pre-Mobile Atrial Fibrillation Apps for Continuous Monitoring of Atrial Fibrillation in a Real-World Setting: Pilot Cohort Study. J Med Internet Res. 2019 Dec 3;21(12):e14909.

413. Zhang P, Burns RD, Fu Y, Godin S, Byun W. Agreement between the Apple Series 1, LifeTrak Core C200, and Fitbit Charge HR with Indirect Calorimetry for Assessing Treadmill Energy Expenditure. Int J Environ Res Public Health. 2019 Oct 10;16(20).

414. Zhang S, Murray P, Zillmer R, Eston RG, Catt M, Rowlands AV. Activity classification using the GENEA: optimum sampling frequency and number of axes. Med Sci Sports Exerc. 2012 Nov;44(11):2228-34.
